# Changes in reasons for visits to primary care after the start of the COVID-19 pandemic: An international comparative study by the *Int**ernational Conso**r**tium of* *P**rimary Care B**i**g* *D**ata Researchers* (INTRePID)

**DOI:** 10.1371/journal.pgph.0003406

**Published:** 2024-08-22

**Authors:** Karen Tu, María C. Lapadula, Jemisha Apajee, Angela Ortigoza Bonilla, Valborg Baste, María S. Cuba-Fuentes, Simon de Lusignan, Signe Flottorp, Gabriela Gaona, Lay Hoon Goh, Christine M. Hallinan, Robert S. Kristiansson, Adrian Laughlin, Zhuo Li, Zheng J. Ling, Jo-Anne Manski-Nankervis, Amy P. P. Ng, Luciano F. Scattini, Javier Silva-Valencia, Wilson D. Pace, Knut-Arne Wensaas, William C. W. Wong, Paula L. Zingoni, John M. Westfall, on behalf of INTRePID

**Affiliations:** 1 Department of Family and Community Medicine, University of Toronto, Toronto, Canada; 2 North York General Hospital, Toronto, Canada; 3 Toronto Western Hospital, Family Health Team, University Health Network, Toronto, Canada; 4 National Centre for Emergency Primary Health Care, NORCE Norwegian Research Centre, Bergen, Norway; 5 Center for Research in Primary Health Care (CINAPS), Universidad Peruana Cayetano Heredia, Lima, Peru; 6 Nuffield Department of Primary Care Health Sciences, University of Oxford, Oxford, United Kingdom; 7 Norwegian Institute of Public Health, Oslo, Norway; 8 Department of General Practice, University of Oslo, Oslo, Norway; 9 DARTNet Institute, Aurora, Colorado, United States of America; 10 Division of Family Medicine, Yong Loo Lin School of Medicine, National University of Singapore, Singapore, Singapore; 11 Department of General Practice and Primary Care, University of Melbourne, Melbourne, Australia; 12 Department of Public Health and Caring Sciences, Uppsala University, Uppsala, Sweden; 13 Department of Family Medicine and Primary Care, University of Hong Kong, Shenzhen Hospital, Shenzhen, China; 14 Primary Care and Family Medicine, Lee Kong Chian School of Medicine, Nanyang Technological University, Singapore, Singapore; 15 Department of Family Medicine and Primary Care, School of Clinical Medicine, Li Ka Shing Faculty of Medicine, University of Hong Kong, Hong Kong, China; 16 Ministry of Health of the Autonomous City of Buenos Aires, Autonomous City of Buenos Aires, Argentina; 17 Research Unit for General Practice, NORCE Norwegian Research Centre AS, Bergen, Norway; University of California San Francisco, UNITED STATES OF AMERICA

## Abstract

**Background:**

The COVID-19 pandemic has reshaped healthcare delivery worldwide.

**Objective:**

To explore potential changes in the reasons for visits and modality of care in primary care settings through the International Consortium of Primary Care Big Data Researchers (INTRePID).

**Methods:**

We conducted a cross-sectional, retrospective study from 2018–2021. We examined visit volume, modality, and reasons for visits to primary care in Argentina, Australia, Canada, China, Peru, Norway, Singapore, Sweden, and the USA. The analysis involved a comparison between the pre-pandemic and pandemic periods.

**Results:**

There were more than 215 million visits from over 38 million patients during the study period in INTRePID primary care settings. Most INTRePID countries experienced a decline in monthly visit rates during the first year of the pandemic, with rate ratios (RR) and 95% confidence intervals (CI) ranging from RR:0.57 (95%CI:0.49–0.66) to RR:0.90 (95%CI:0.83–0.98), except for in Canada (RR:0.99, 95%CI:0.94–1.05) and Norway (RR:1.00, 95%CI:0.92–1.10), where rates remained stable and in Australia where rates increased (RR:1.19, 95%CI:1.11–1.28). Argentina, China, and Singapore had limited or no adoption of virtual care, whereas the remaining INTRePID countries varied in the extent of virtual care utilization. In Peru, virtual visits accounted for 7.34% (95%CI:7.33%-7.35%) of all interactions in the initial year of the pandemic, dipping to 5.22% (95%CI:5.21%-5.23%) in the subsequent year. However, in Canada 75.30% (95%CI:75.20%-75.40%) of the visits in the first year were virtual, decreasing to 62.77% (95%CI:62.66%-62.88%) in the second year. Diabetes, hypertension and/or hyperlipidemia and general health exams were in the top 10 reasons for visits in 2019 for all countries. Anxiety, depression and/or other mental health related reasons were among the top 10 reasons for virtual visits in all countries that had virtual care.

**Conclusions:**

The pandemic resulted in changes in reasons for visits to primary care, with virtual care mitigating visit volume disruptions in many countries.

## Introduction

The COVID-19 worldwide pandemic has been an unprecedented event that is something that we have never seen in our lifetimes. It impacted not only health but also all facets and disciplines of healthcare delivery. Primary care is the cornerstone of many healthcare systems around the world. Initiated with the onset of the pandemic we developed the International Consortium of Primary Care Big Data Researchers (INTRePID-www.intrepidprimarycare.org) to perform international comparative studies in primary care using existing big data sources in several different countries currently spanning 5 of the 7 continents of the world [1]. INTRePID collects primary care data from electronic medical records (EMRs) or administrative databases. Primary care providers and/or researchers from participating countries extract, curate and verify the data within their respective countries and are actively involved in the interpretation of the findings within the local context [2,3]. Data from each country is transferred to the University of Toronto for central data harmonization, standardization of definitions across participating nations and central analysis.

Prior to the pandemic, a number of studies have looked at reasons for visits to primary care in various countries [4]. The COVID-19 pandemic led to an abrupt decline in the use of healthcare services worldwide [5,6] and a few studies have found a reduction in the assessment of cardiovascular risk factors [7], children’s immunization rates [8], and health screening [9,10]. Moreover, some studies suggested a discontinuity of chronic disease monitoring [11,12] and an increase in non-COVID mortality related to delays in care and management [8].

In the first year of the COVID-19 pandemic, a decline in in-person visits to primary care was seen across all the INTRePID countries [13]. While each INTRePID country had its own strategies to deal with the pandemic, several introduced or expanded virtual care to mitigate the drop in in-person visits to primary care physicians (PCPs) [13]. A scoping review that included 32 studies from 18 countries across 6 continents on the impact of COVID-19 on primary care identified that the rapid transition or expansion to virtual care in primary care was an important COVID-19 mitigation strategy [14]. They found that the transition to telemedicine in primary care was reported to have both positive and negative aspects. Amongst the positive effects reported in several studies were that telemedicine enhanced access to primary care while ensuring patient and provider safety (decreasing exposure risk), while other studies found it increased inequity of health care by reducing access, in particular for those with barriers in the use of technology and the severe mentally ill [14]. Although the introduction or expansion of virtual visits in primary care has met with patient satisfaction [15], it has been acknowledged that telemedicine can present challenges in developing countries [5]. These challenges arise from disparities in medical digital infrastructure across regions, unequal internet access, and a lack of comprehensive legislation governing virtual care practices. A recent study has highlighted the complexities of virtual care adoption in Latin American countries [16]. The study underscores the urgent need for targeted interventions to address infrastructure gaps and policy deficiencies, highlighting the importance of equitable access to technology and robust regulatory frameworks for the successful integration of virtual care into healthcare systems.

While the decline in healthcare utilization in primary care for the first year of the pandemic has been well documented [10,13,17–19], the pandemic spread, and public policy measures continued into the second year of the pandemic and beyond [8]. We assessed if the decline in primary care in-person visits and the uptake of virtual care initially seen with the onset of the pandemic continued through the second year, as there are some aspects of primary care that require in-person care and cannot be delivered virtually [20,21]. We identified the most common conditions presenting to primary care prior to the pandemic and examined the changes in reasons for visits to identify potential gaps in necessary healthcare that might have resulted from pandemic related changes in care delivery.

## Materials and methods

### Study design and population

A retrospective analysis was conducted on primary care visits in nine countries from January 2018 to December 2021. Participant countries in this study included Argentina, Australia, Canada, China, Norway, Peru, Singapore, Sweden, and the USA. The study period offered a baseline of approximately two years prior to the COVID pandemic and the first two years of the pandemic.

### Data source and procedures

Data on visit volume, modality and reasons for visits to PCPs or primary care clinics were obtained through the INTRePID Consortium. Data was accessed in INTRePID countries at varying times and ran from throughout 2022 through to Aug 2023. The extent of data coverage varied among countries, with some providing national data and others from specific health subsystems or regions. Data were collected using various coding systems, including ICPC-2, SNOMED CT, ICD-10 and variants (ICD-10 CM and ICD-10 AM) and the Ontario Health Insurance Program (OHIP) in Ontario, Canada, which uses an ICD 8–9 hybrid coding system. (See S1 Data and https://www.intrepidprimarycare.org/resources for detailed descriptions of individual country data).

First, we examined country specific patterns in monthly visit volume and visit modality (in-person and virtual), new COVID-19 cases per million, national Containment and Health Indices and COVID-19 vaccinations per hundred [22]. For comparison purposes across countries, visit volume to primary care was standardized to the country specific 2019 (pre-pandemic) monthly visit rate.

To identify the most frequent reasons for pre-pandemic visits, we reviewed the top ten reasons for visits in 2019. Subsequently, we looked at the top ten reasons for visits during the pandemic by examining the in-person and virtual care visits (for countries that had substantial virtual care) in 2020. Virtual care was defined as a visit made by telephone, video, or in the case of Norway, also included electronic consultations.

Last, we looked at the visit patterns for six common conditions seen in primary care: anxiety/depression, cough/cold/upper respiratory tract infection (URTI), general health exams (also referred to as check-ups, general medical exams, periodic health exams, health maintenance exams or preventive care visits), diabetes, hypertension and hypercholesterolemia. Our choice to focus on diabetes, hypertension, hypercholesterolemia, and anxiety/depression stemmed from their widespread prevalence across all countries, significant health implications and chronic characteristics. Preventive care was crucial, given its fundamental role in primary care. Additionally, cough, cold or URTIs were common across the nine studied countries and showed marked changes with the onset of the pandemic. Monthly visit volumes for each condition were obtained by aggregating visits for all codes that depict these conditions (S1–S6 Tables).

### Statistical analysis

Causality and COVID-19 cases, containment and health indices and vaccination rates.

We conducted the Granger causality test in pairwise combinations to explore whether there was evidence of relation or potential causal direction between standardized monthly visits and COVID-19 new cases, individual countries’ Containment and Health Index, or vaccination rates per hundred. Monthly data for this analysis incorporated a one-month lag.

### Comparing monthly visit rates

We calculated monthly visit rates overall and for the common conditions, in three time periods; 1. pre-pandemic (January 2018 to February 2020), 2. first year of the pandemic (pandemic period 1-April 2020 to February 2021) and, 3. into the second year of the pandemic (pandemic period 2-March 2021 to December 2021), as total visit volume in a time period divided by the number of months in the same time period. To account for a transition period following the WHO’s global pandemic declaration on March 11, 2020, we excluded March 2020 from the pandemic period for all countries except China. China declared a state of emergency on January 23, 2020; thus, we used February 2020 as the start of the pandemic period 1 and February 2021 as the start of pandemic period 2 in China. Since data from Peru was only available from 2019 onwards, the start of the pre-pandemic period for Peru was January 2019.

We used negative binomial regression to obtain rate ratios (RR) and 95% confidence intervals (95% CI) comparing monthly visit rate in: 1. pandemic period 1 versus pre-pandemic, 2. pandemic period 2 versus pandemic period 1 and 3. pandemic period 2 versus pre-pandemic. We calculated the percentage of virtual visits along with their 95% confidence intervals, and then compared RR of the percentages of virtual visits between the studied time periods.

### Using pre-pandemic data to obtain expected visit volume during the pandemic

In this analysis we defined two time periods: pre-pandemic, as described in the previous analysis, and the pandemic period which is a combination of pandemic period 1 and 2 from above. We modelled pre-pandemic monthly visit volume using negative binomial regression with time as the main predictor and modelled residuals as autoregressive AR (1) processes to adjust for autocorrelation in the data. The autocorrelation function (ACF) of visit volume suggested seasonality at lag 6 and 12 for some conditions, therefore, we included sine and cosine functions of period 6 and 12 in the models to adjust for seasonality. Additional predictors were added to the models for Norway, Sweden and China to model changes to the underlying patterns in the data. For Norway and Sweden, an indicator variable (0/1) for the month of July was used to model the strong pattern in that month. For China, an indicator variable for the period from January 2019 to January 2020 was added to the model because of the structural change in the volume of visits for anxiety/depression and hypercholesterolemia. Similarly, two indicator variables for December 2019 and January 2020 were added to model the sharp increase in preventive care in China during December 2019 and January 2020. (See S1 Data China for description of specific structural changes in China)

These fitted models were used to obtain the expected number of visits with the 95% confidence intervals for the different conditions in each month of the pandemic period. We then calculated the absolute difference between the expected and the observed volume of visits and obtained the 95% confidence interval for each month of the pandemic period.

All analyses were performed with SAS version 9.4 (SAS Institute). Figures were created with R version 4.3.1. The data used for the analysis is provided in S2 Data and S3 Data.

### Ethics statement

This study received approval from the University of Toronto Research Ethics Board (REB) (#40943). As with the nature of these types of studies performing retrospective analysis of a large volume of de-identified data, waiver of consent is granted from the REB. The data used and presented in this study are aggregated not individual level and therefore it is impossible to identify individuals, thereby posing no risk or impact to participants. Requiring consent would bias the data making the results to be not generalizable and given the millions of patients involved in this study, it would be both impossible and impracticable to require consent and address the research questions. All data used in this study was de-identified aggregate data and none of the authors had access to information that could identify individual participants during or after data collection.

## Results

### Visit volume and modality

**Fig 1:**
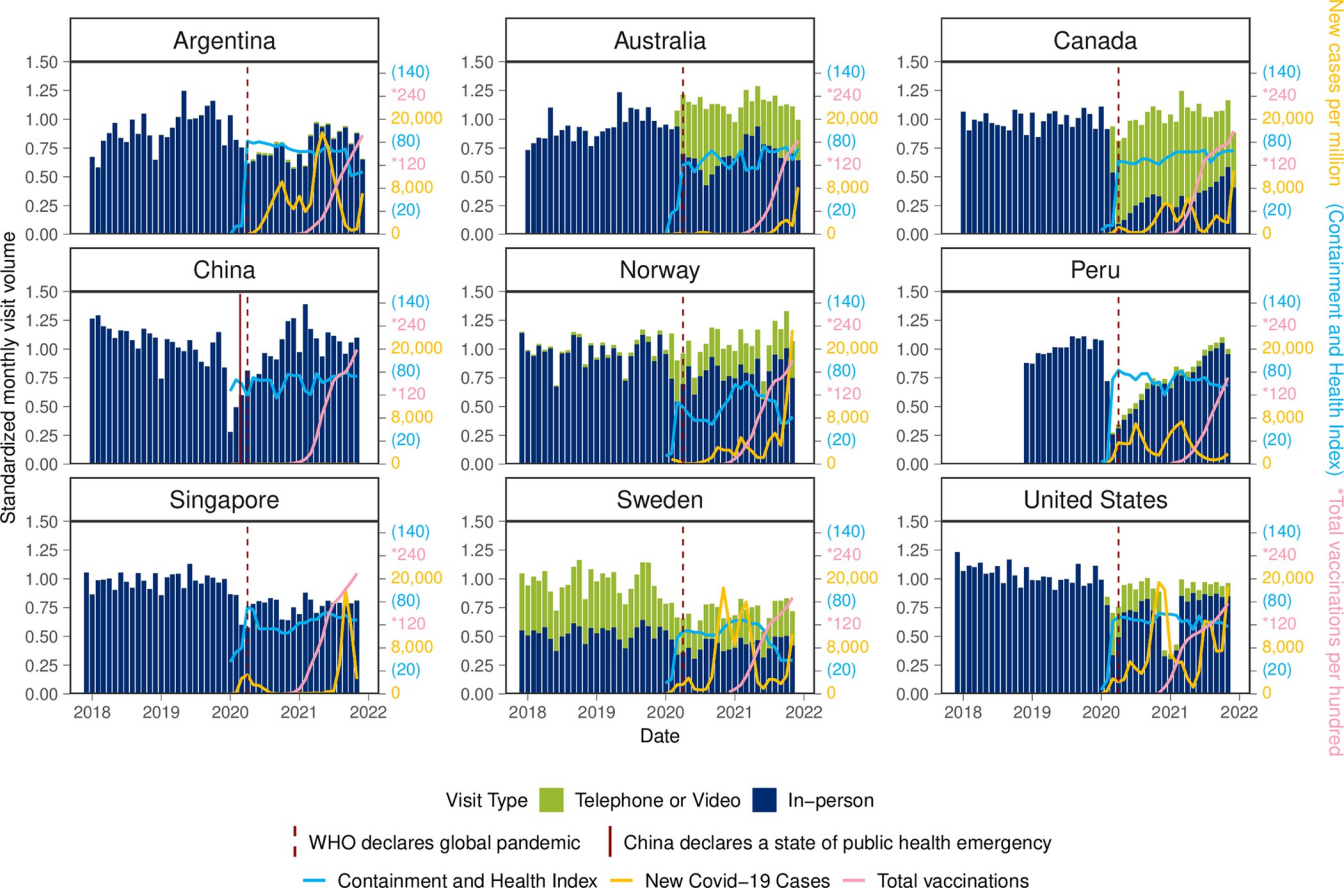
Changes in primary care visits, COVID-19 spread, Containment and Health Index and vaccination uptake in INTRePID countries in 2018–2021. Source: Official data including new COVID cases, Containment and Health Index, and vaccination rates collected by Our World in Data (https://ourworldindata.org/coronavirus).

From 2018 to 2021, there were over 215 million primary care patient visits reported from over 38 million patients among our data sources in primary care settings in the nine INTRePID countries. Argentina, China, Peru, Singapore and the USA had an initial drop in visit volume with the onset of the pandemic while Australia, Canada, Norway and Sweden did have fewer in-person visits but introduced or expanded virtual care which mitigated the decrease in in-person visits (Fig 1). China and Peru had large drops in visits initially but both countries rebounded in 2021. USA had a drop with the onset of the pandemic and an even larger drop at the beginning of 2021 coinciding with a large COVID-19 spike (Fig 1). While the USA had the earliest uptake of COVID vaccinations, the other INTRePID countries soon increased with China and Singapore having the highest number of vaccinations per hundred at the end of 2021. In most countries, the Containment and Health Index, COVID-19 cases and vaccination uptake were not related and were not associated with visit volume in the following month. We only found a significant Granger causality relationship between the Containment and Health Index and visit volume in the USA (p = 0.031) and Singapore (p = 0.011) and between vaccination uptake and visit volume in Australia (p = 0.001).

The monthly visit rate decreased significantly in the first year of the pandemic period compared to the pre-pandemic period in all countries except for Australia, Canada and Norway, where the increase in virtual visits offset the decrease in in-person visits (Table 1). Into the second year of the pandemic, the monthly visit rate increased for all countries compared to the first year (Table 1). While Australia, Canada, Peru and the USA introduced virtual care with the onset of the pandemic, there was negligible uptake in China and Singapore, and minimal uptake in Argentina. In Norway and Sweden, countries that had pre-existing virtual care, the proportion of virtual visits increased during the pandemic. However, virtual care declined in all countries during the second year of the pandemic (Table 2).

**Table 1:** Visit rates and change in monthly visit rates during pre-pandemic and pandemic periods for the INTRePID countries.

Common diseases and total visit volume (2018–2021)		Monthly visit rate (SD)			Change in monthly visits rate		
		Pre-pandemic period^a^	Pandemic period 1^b^	Pandemic period 2^c^	Pandemic period 1 vs pre-pandemic period RR (95% CI)	Pandemic period 2 vs pandemic period 1 RR (95% CI)	Pandemic period 2 vs pre-pandemic period RR (95% CI)
**Argentina**							
Total visits	4,439,955	99,523 (17,001)	74,400 (7,960)	95,187 (10.498)	**0.75 (0.68–0.83)**	**1.28 (1.13–1.45)**	0.96 (0.86–1.06)
Anxiety or depression	89,588	1,956 (266)	1,604 (271)	1,978 (230)	**0.82 (0.74–0.90)**	**1.23 (1.10–1.39)**	1.01 (0.91–1.12)
Cough, cold or URTI	100,011	2,889 (1,338)	389 (63)	1,857 (1,042)	**0.13 (0.10–0.18)**	**4.78 (3.29–6.94)**	**0.64 (0.47–0.88)**
Preventive care	987,912	27,177 (4,253)	8,397 (2,997)	17,283 (1,607)	**0.31 (0.27–0.36)**	**2.06 (1.72–2.47)**	**0.64 (0.55–0.74)**
Diabetes	119,840	2,091 (449)	2,675 (346)	3,388 (383)	**1.28 (1.13–1.45)**	**1.27 (1.09–1.47)**	**1.62 (1.43–1.84)**
Hypertension	95,330	2,000 (393)	1,842 (206)	2,140 (209)	0.92 (0.82–1.03)	**1.16 (1.02–1.33)**	1.07 (0.95–1.20)
Hypercholesterolemia	29,278	585 (140)	505 (147)	807 (60)	0.86 (0.73–1.01)	**1.60 (1.32–1.94)**	**1.38 (1.17–1.63**)
**Australia**							
Total visits	6,604,073	125,346 (15,793)	149,715 (8,823)	155,384 (10,938)	**1.19 (1.11–1.28)**	1.04 (0.95–1.13)	**1.24 (1.15–1.33)**
Anxiety or depression	169,409	3,221 (319)	3,928 (418)	3,878 (336)	**1.22 (1.14–1.30)**	0.99 (0.91–1.07)	**1.20 (1.12–1.29)**
Cough, cold or URTI	203,598	5,216 (1,948)	2,265 (902)	3,427 (1,479)	**0.43 (0.33–0.57)**	**1.51 (1.09–2.10)**	**0.66 (0.50–0.87)**
Preventive care	62,586	1,258 (155)	1,334 (220)	1,380 (118)	1.06 (0.97–1.16)	1.03 (0.93–1.15)	**1.09 (1.00–1.20)**
Diabetes	100,908	1,934 (172)	2,282 (144)	2,332 (132)	**1.18 (1.12–1.24)**	1.02 (0.96–1.09)	**1.21 (1.14–1.27)**
Hypertension	131,477	2,576 (287)	2,883 (247)	2,969 (186)	**1.12 (1.05–1.20)**	1.03 (0.95–1.12)	**1.15 (1.08–1.24)**
Hypercholesterolemia	42,621	726 (77)	1,045 (138)	1,142 (97)	**1.44 (1.33–1.55)**	1.09 (1.00–1.20)	**1.57 (1.46–1.70)**
**Canada**							
Total visits	3,454,707	70,891 (5,378)	70,328 (6,675)	77,085 (6,893)	0.99 (0.94–1.05)	**1.10 (1.02–1.17)**	**1.09 (1.03–1.15)**
Anxiety or depression	301,276	5,318 (387)	7,245 (417)	7,653 (875)	**1.36 (1.29–1.44)**	1.06 (0.99–1.13)	**1.44 (1.36–1.52)**
Cough, cold or URTI	137,110	3,570 (979)	1,774 (512)	1,946 (475)	**0.50 (0.42–0.59)**	1.10 (0.88–1.36)	**0.55 (0.45–0.66)**
Preventive care	148,957	3,726 (343)	2,189 (232)	2,564 (368)	**0.59 (0.55–0.63)**	**1.17 (1.07–1.28)**	**0.69 (0.64–0.74)**
Diabetes	192,037	4,202 (403)	3,525 (450)	3,993 (482)	**0.84 (0.78–0.90)**	**1.13 (1.03–1.24)**	0.95 (0.88–1.03)
Hypertension	182,567	4,094 (414)	3,326 (334)	3,541 (412)	**0.81 (0.76–0.87)**	1.06 (0.98–1.16)	**0.86 (0.80–0.93)**
Hypercholesterolemia	34,298	651 (68)	701 (190)	902 (101)	1.08 (0.96–1.21)	**1.29 (1.12–1.47)**	**1.39 (1.23–1.56)**
**China**							
Total visits	317,971	6,928 (897)	5,515 (1,915)	7,144 (820)	**0.80 (0.69–0.92)**	**1.30 (1.09–1.55)**	1.03 (0.88–1.20)
Anxiety or depression	12,349	269 (150)	372 (130)	106 (21)	1.39 (1.00–1.92)	**0.29 (0.19–0.42)**	**0.40 (0.28–0.55)**
Cough, cold or URTI	5,897	184 (49)	66 (35)	46 (23)	**0.36 (0.28–0.46)**	**0.70 (0.51–0.96)**	**0.25 (0.19–0.33)**
Preventive care	19,491	223 (207)	739 (426)	459 (223)	**3.31 (1.91–5.76)**	0.62 (0.32–1.20)	**2.06 (1.16–3.63)**
Diabetes	11,692	98 (21)	230 (231)	591 (217)	**2.36 (1.71–3.25)**	**2.57 (1.76–3.75)**	**6.06 (4.36–8.42)**
Hypertension	25,135	193 (39)	421 (434)	1,386 (217)	**2.18 (1.59–2.98)**	**3.29 (2.27–4.77)**	**7.17 (5.19–9.89)**
Hypercholesterolemia	6,421	105 (49)	167 (88)	164 (39)	**1.59 (1.16–2.19)**	0.98 (0.68–1.43)	**1.56 (1.13–2.17)**
**Norway**							
Total visits	59,074,998	1,203,158 (140,469)	1,207,084 (145,833)	1,315,599 (198,527)	1.00 (0.92–1.10)	1.09 (0.98–1.21)	1.09 (1.00–1.20)
Anxiety or depression	2,710,131	55,005 (8,290)	58,674 (7,934)	57,573 (9,293)	1.07 (0.96–1.19)	0.98 (0.86–1.12)	1.05 (0.94–1.17)
Cough, cold or URTI	2,119,298	53,489 (14,418)	22,934 (4,130)	39,980 (30,649)	**0.43 (0.33–0.56)**	**1.74 (1.25–2.43)**	**0.75 (0.56–0.99)**
Preventive care	1,135,748	21,922 (5,995)	23,949 (13,487)	28,525 (11,542)	1.09 (0.86–1.39)	1.19 (0.89–1.60)	**1.30 (1.01–1.67)**
Diabetes	1,570,840	31,543 (4,756)	33,426 (5,556)	35,095 (6,959)	1.06 (0.93–1.20)	1.05 (0.90–1.23)	1.11 (0.98–1.27)
Hypertension	2,036,344	43,223 (7,022)	41,481 (7,871)	41,794 (8,561)	0.96 (0.84–1.10)	1.01 (0.85–1.19)	0.97 (0.84–1.11)
Hypercholesterolemia	304,780	6,335 (1,252)	6,197 (1,535)	6,652 (1,604)	0.98 (0.82–1.16)	1.07 (0.87–1.32)	1.05 (0.88–1.25)
**Peru** ^**d**^							
Total visits	133,824,746	4,426,771 (343,719)	2,501,496 (744,077)	4,117,445 (476,266)	**0.57 (0.49–0.66)**	**1.65 (1.40–1.93)**	0.93 (0.80–1.08)
Anxiety or depression	3,113,164	59,231 (8,081)	84,199 (22,628)	131,573 (12,934)	**1.42 (1.22–1.65)**	**1.56 (1.33–1.84)**	**2.22 (1.91–2.59)**
Cough, cold or URTI	4,233,489	151,532 (21.792)	68,310 (17,025)	124,313 (15,013)	**0.45 (0.39–0.52)**	**1.82 (1.56–2.12)**	**0.82 (0.71–0.95)**
Preventive care	12,819,826	424,678 (25,846)	153,622 (72,340)	491,496 (127,660)	**0.36 (0.29–0.46)**	**3.20 (2.48–4.13)**	1.16 (0.91–1.47)
Diabetes	2,203,095	54,782 (4,555)	54,533 (15,668)	79,540 (8,357)	1.00 (0.86–1.15)	**1.46 (1.25–1.71)**	**1.45 (1.25–1.69)**
Hypertension	3,140,624	70,612 (5,717)	86,031 (17,976)	114,561 (7,214)	**1.22 (1.10–1.35)**	**1.33 (1.19–1.49)**	**1.62 (1.46–1.81)**
Hypercholesterolemia	1,534,227	54,172 (5,908)	22,971 (12,929)	48,765 (11,451)	**0.42 (0.32–0.57)**	**2.12 (1.54–2.93)**	0.90 (0.66–1.22)
**Singapore**							
Total visits	4,444,278	103,265 (7,129)	75,821 (9,825)	83,510 (4,670)	**0.73 (0.69–0.78)**	**1.10 (1.03–1.18)**	**0.81 (0.76–0.86)**
Anxiety or depression	16,758	301 (41)	358 (71)	469 (35)	**1.19 (1.08–1.31)**	**1.31 (1.16–1.47)**	**1.56 (1.41–1.72)**
Cough, cold or URTI	648,161	19,019 (2,342)	5,524 (1,374)	7,570 (1,256)	**0.29 (0.26–0.33)**	**1.37 (1.19–1.58)**	**0.40 (0.35–0.45)**
Preventive care	104,426	2,784 (219)	2,165 (948)	547 (81)	**0.78 (0.65–0.93)**	**0.25 (0.20–0.32)**	**0.20 (0.16–0.24)**
Diabetes	652,349	14,029 (997)	13,011 (1,677)	13,090 (762)	**0.93 (0.87–0.98)**	1.01 (0.94–1.08)	**0.93 (0.88–0.99)**
Hypertension	514,274	10,878 (989)	9,865 (1,073)	11,333 (794)	**0.91 (0.85–0.97)**	**1.15 (1.06–1.24)**	1.04 (0.98–1.11)
Hypercholesterolemia	217,611	3,978 (407)	4,624 (827)	5,942 (401)	**1.16 (1.07–1.26)**	**1.28 (1.16–1.42)**	**1.49 (1.37–1.62)**
**Sweden**							
Total visits	6,376,378	136,199 (15,824)	122,923 (12,693)	134,063 (16,128)	**0.90 (0.83–0.98)**	1.09 (0.99–1.20)	0.98 (0.91–1.07)
Anxiety or depression	112,426	2,133 (355)	2,330 (372)	2,889 (467)	1.09 (0.97–1.23)	**1.24 (1.08–1.43)**	**1.35 (1.20–1.53)**
Cough, cold or URTI	52,850	1,371 (384)	727 (346)	749 (422)	**0.53 (0.41–0.69)**	1.03 (0.75–1.42)	**0.55 (0.42–0.72)**
Preventive care	29,375	671 (92)	521 (78)	565 (92)	**0.78 (0.70–0.86)**	1.08 (0.96–1.23)	**0.84 (0.76–0.94)**
Diabetes	86,958	1,728 (470)	1,767 (551)	2,065 (659)	1.02 (0.81–1.29)	1.17 (0.88–1.55)	1.20 (0.94–1.52)
Hypertension	103,909	2,207 (508)	1,941 (474)	2,271 (573)	0.88 (0.73–1.05)	1.17 (0.94–1.46)	1.03 (0.85–1.24)
Hypercholesterolemia	3,256	70 (20)	57 (14)	73 (21)	0.82 (0.66–1.01)	1.28 (0.99–1.65)	1.05 (0.84–1.30)
**United States**							
Total visits	818,877	18,646 (1,475)	14,317 (4,186)	16,148 (3,012)	**0.77 (0.67–0.88)**	1.13 (0.95–1.33)	**0.87 (0.75–1.00)**
Anxiety or depression	82,440	1,838 (185)	1,410 (615)	1,759 (530)	0.77 (0.59–1.00)	1.25 (0.90–1.73)	0.96 (0.72–1.26)
Cough, cold or URTI	24,015	728 (352)	200 (102)	223 (79)	**0.27 (0.20–0.38)**	1.12 (0.75–1.65)	**0.31 (0.22–0.43)**
Preventive care	149,192	3,219 (313)	2,524 (1,406)	3,553 (1,162)	0.78 (0.57–1.08)	1.41 (0.95–2.09)	1.10 (0.79–1.54)
Diabetes	82,521	1,949 (237)	1,280 (620)	1,619 (533)	**0.66 (0.47–0.93)**	1.27 (0.83–1.92)	0.83 (0.58–1.19)
Hypertension	120,012	2,809 (294)	1,872 (913)	2,443 (812)	**0.67 (0.47–0.94)**	1.30 (0.86–1.98)	0.87 (0.61–1.24)
Hypercholesterolemia	118,480	2,729 (286)	1,897 (941)	2,482 (799)	**0.70 (0.51–0.95)**	1.31 (0.89–1.92)	0.91 (0.66–1.26)

^a^Pre-pandemic period was defined as January 1, 2018, to February 28, 2020, except for China, where it extended to January 31, 2020, and Peru, where it spanned from January 1, 2019, to February 28, 2020.

^b^Pandemic period 1 was defined as April 1, 2020-February 28, 2021, except for China, where it extended from February 1, 2020, to January 31, 2021.

^c^Pandemic period 2 was defined as March 1, 2021-December 31, 2021, except for China, where it extended from February 1, 2021, to December 31, 2021.

^d^Peru data was only available from 2019 to 2021.

**Table 2:** Proportion of virtual visits and relative change in the pre-pandemic and pandemic periods for the INTRePID countries.

Country	Number of virtual visits (2018–2021)	Percentage (95% CI) of virtual visits			Change in percentage of virtual visits by periods^d^		
		Pre-pandemic period^a^	Pandemic period 1^b^	Pandemic period 2^c^	Pandemic period 1 vs pre-pandemic period RR (95% CI)	Pandemic period 2 vs pandemic period 1 RR (95% CI)	Pandemic period 2 vs pre-pandemic period RR (95% CI)
**Argentina **	31,677	0.09% (0.09%-0.09%)	2.24% (2.21%-2.27%)	1.16% (1.14%-1.18%)	-	**0.52 (0.51–0.53)**	-
**Australia **	1,274,865	-	44.00% (43.92%-44.08%)	34.21% (34.14%-34.28%)	-	**0.78 (0.78–0.78)**	-
**Canada **	1,095,079	0.01% (0.01%-0.01%)	75.30% (75.20%-75.40%)	62.77% (62.66%-62.88%)	-	**0.83 (0.83–0.84)**	-
**China **	-	-	-	-	-	-	-
**Norway **	7,690,008	2.41 (2.40%-2.42%)	25.05% (25.03%-25.07%)	23.89% (23.87%-23.91%)	**10.41 (10.38–10.43)**	**0.95 (0.95–0.95)**	**9.92 (9.90–9.95)**
**Peru** ^**e**^	4,186,530	0.02% (0.02%-0.02%)	7.34% (7.33%-7.35%)	5.22% (5.21%-5.23%)	-	**0.71 (0.71–0.71)**	-
**Singapore **	3,845	-	0.02% (0.02%-0.02%)	0.43% (0.42%-0.44%)	-	-	-
**Sweden **	2,207,052	29.63% (29.58%-29.68%)	41.84% (41.76%-41.92%)	39.9% (39.82%-39.98%)	**1.41 (1.41–1.42)**	**0.95 (0.95–0.96)**	**1.35 (1.34–1.35)**
**United States**	57,350	-	24.28% (24.07%-24.49%)	11.05% (10.90%-11.20%)	-	**0.46 (0.45–0.46)**	-
**Total **	16,546,406						

### Top 10 reasons for visit

**Fig 2:**
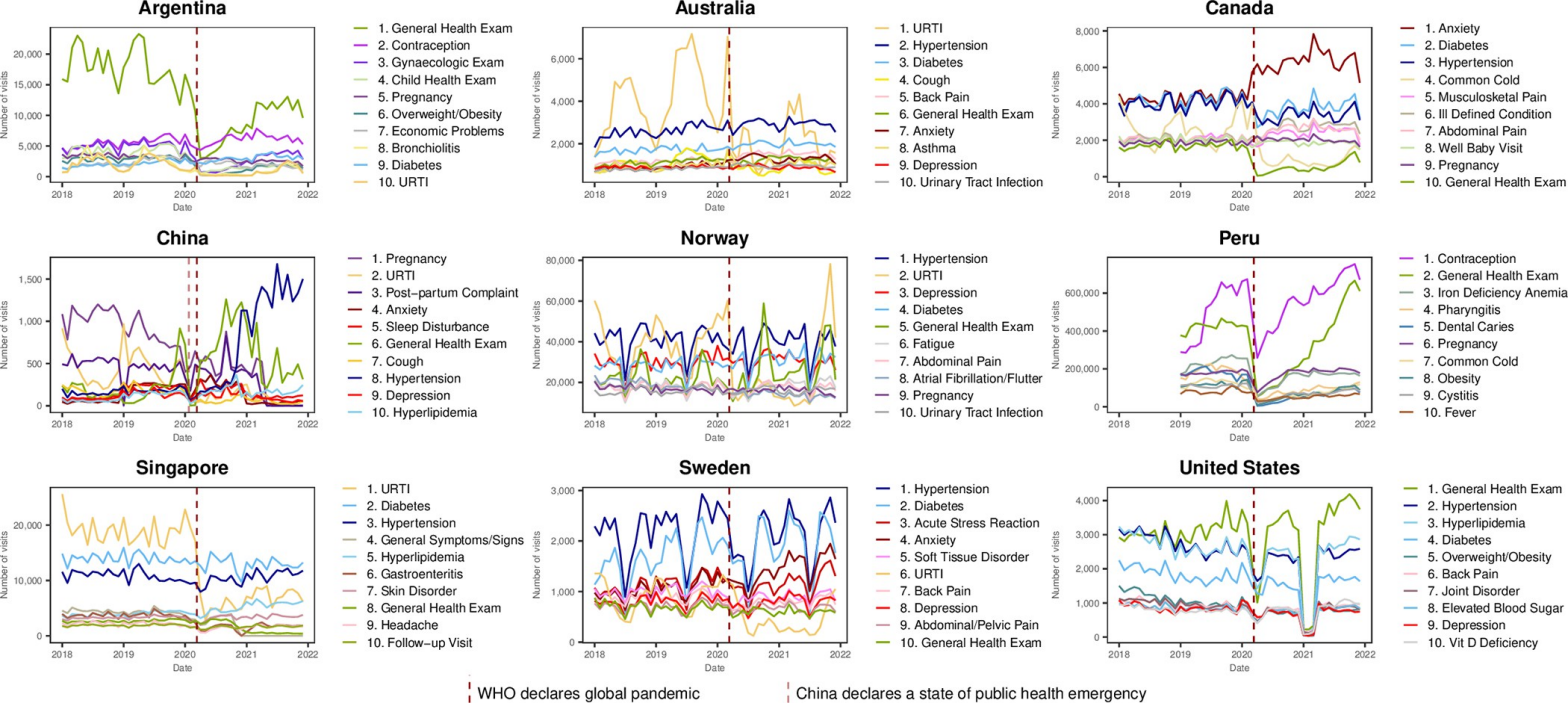
Top 10 reasons for visits in 2019.

Diabetes, hypertension and/or hyperlipidemia and general health exams were in the top 10 reasons for visits in 2019 for all countries. Anxiety and/or depression were in the top 10 reasons for visits in all INTRePID countries except Singapore, Peru, and Argentina whilst, URTI or cold was in the top 10 for all countries but the USA. Pregnancy was in the top 10 reasons for visits in Argentina, Canada, China, Norway, and Peru. It was also interesting to note that overweight/obesity was in the top 10 for Argentina, Peru and the USA, while contraception was in the top 10 in both Argentina and Peru (Fig 2 and S7 Table).

**Fig 3:**
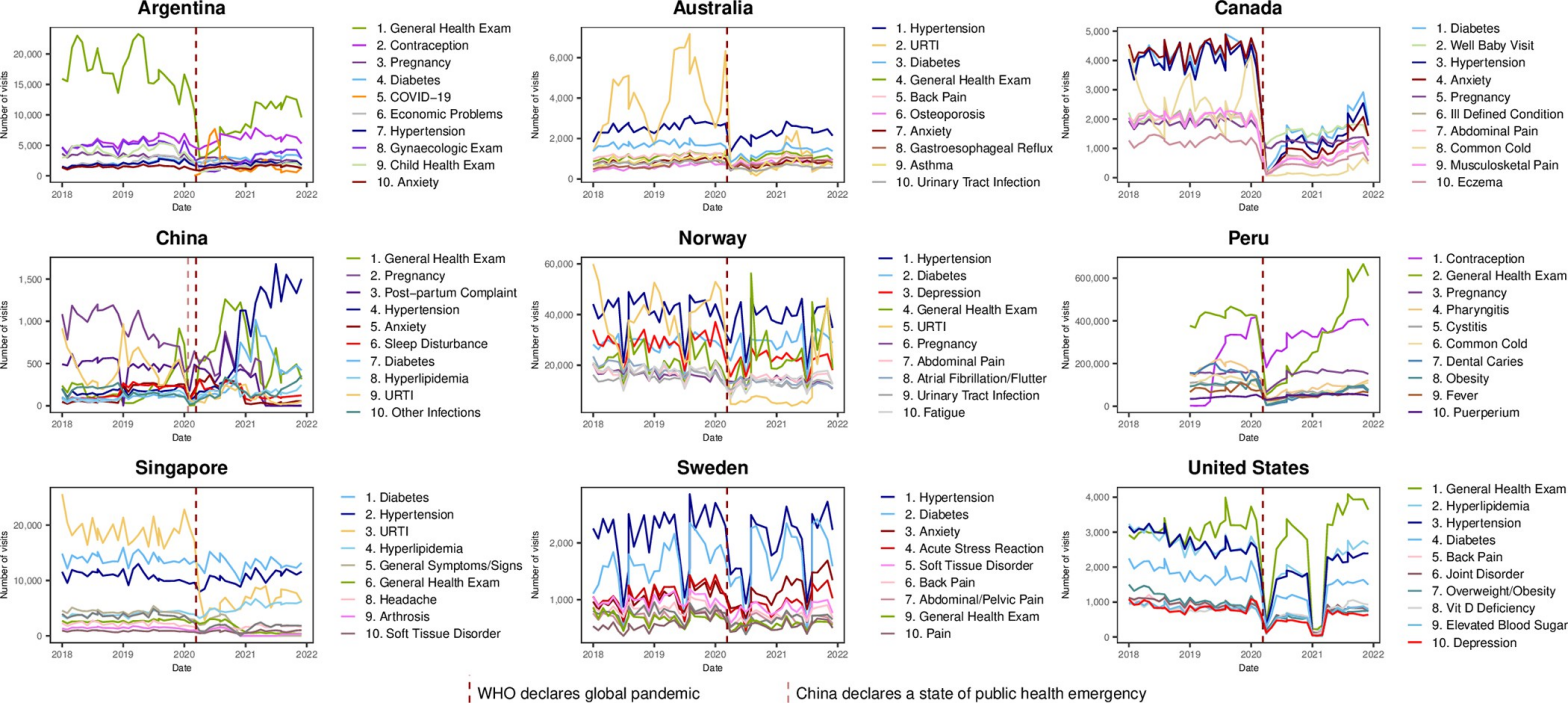
Top 10 reasons for in-person visits in 2020.

**Fig 4:**
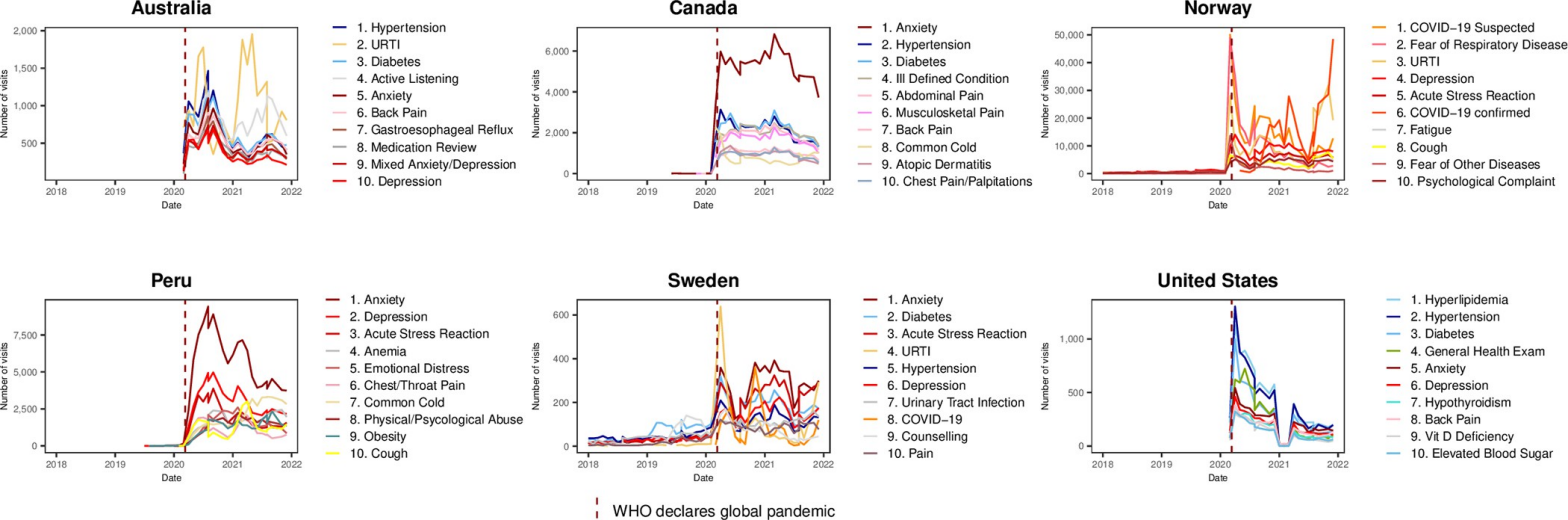
Top 10 reasons for virtual visits in 2020.

Though many countries had an initial drop in general health exams it rebounded and stayed in the top 10 reasons for in-person visits in 2020 for all countries except Canada. (Fig 3 and S8 Table). COVID-19 came into the top 10 reasons for visits virtually in 2020 in Argentina, Norway and Sweden and also in the top 10 reasons for in-person visits in 2020 in Argentina (Fig 3). Anxiety, depression and/or other mental health related reasons were among the top 10 reasons for virtual visits in all countries that had virtual care (Fig 4 and S9 Table).

### Common conditions

**Fig 5:**
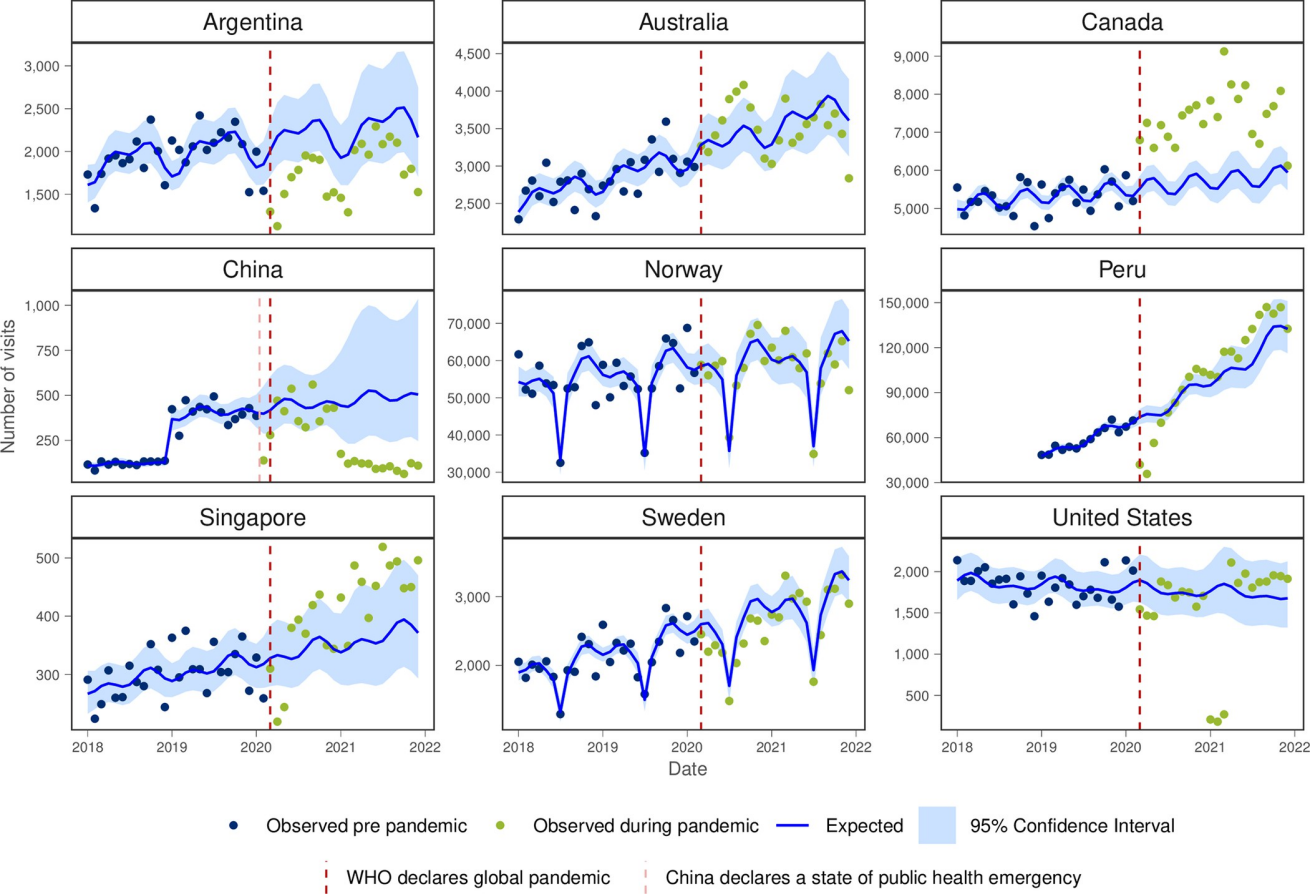
Number of observed and expected visits for anxiety and/or depression in INTRePID countries 2018–2021.

On examining the common conditions presenting to primary care, anxiety and/or depression visits tended to be higher than expected during the pandemic for Peru, Singapore and Canada where there was a large increase in anxiety/depression with the onset of the pandemic that was sustained throughout the study period (Fig 5). Monthly visit rates in the first year of the pandemic were higher than pre-pandemic in Australia, Canada, Peru and Singapore (Table 1).

**Fig 6:**
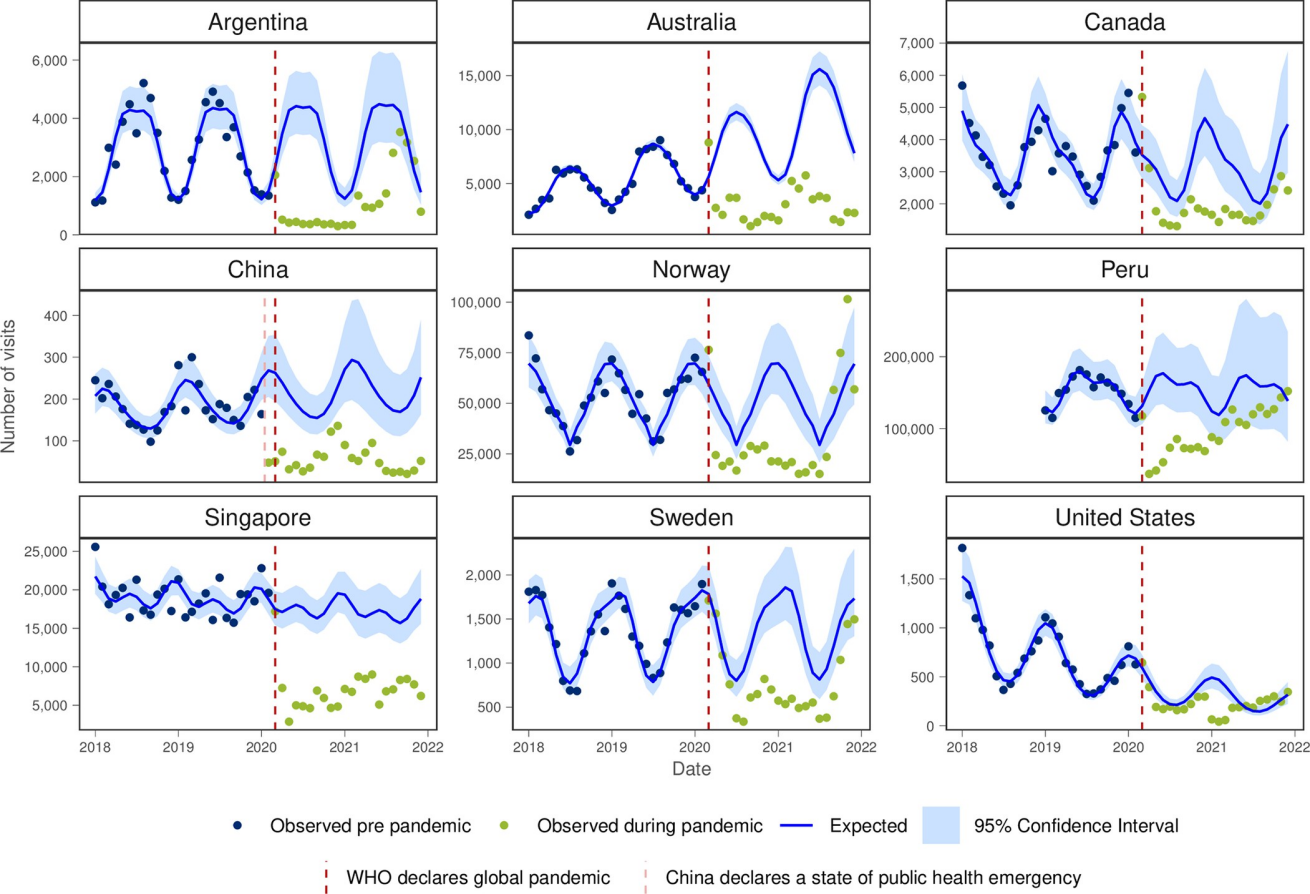
Number of observed and expected visits for cough, cold or URTI in INTRePID countries 2018–2021.

For cough, cold or URTI all INTRePID countries, except in the second half of 2021 in USA, had both lower than expected visit numbers and lower monthly visit rates throughout the pandemic (Fig 6 and Table 1).

**Fig 7:**
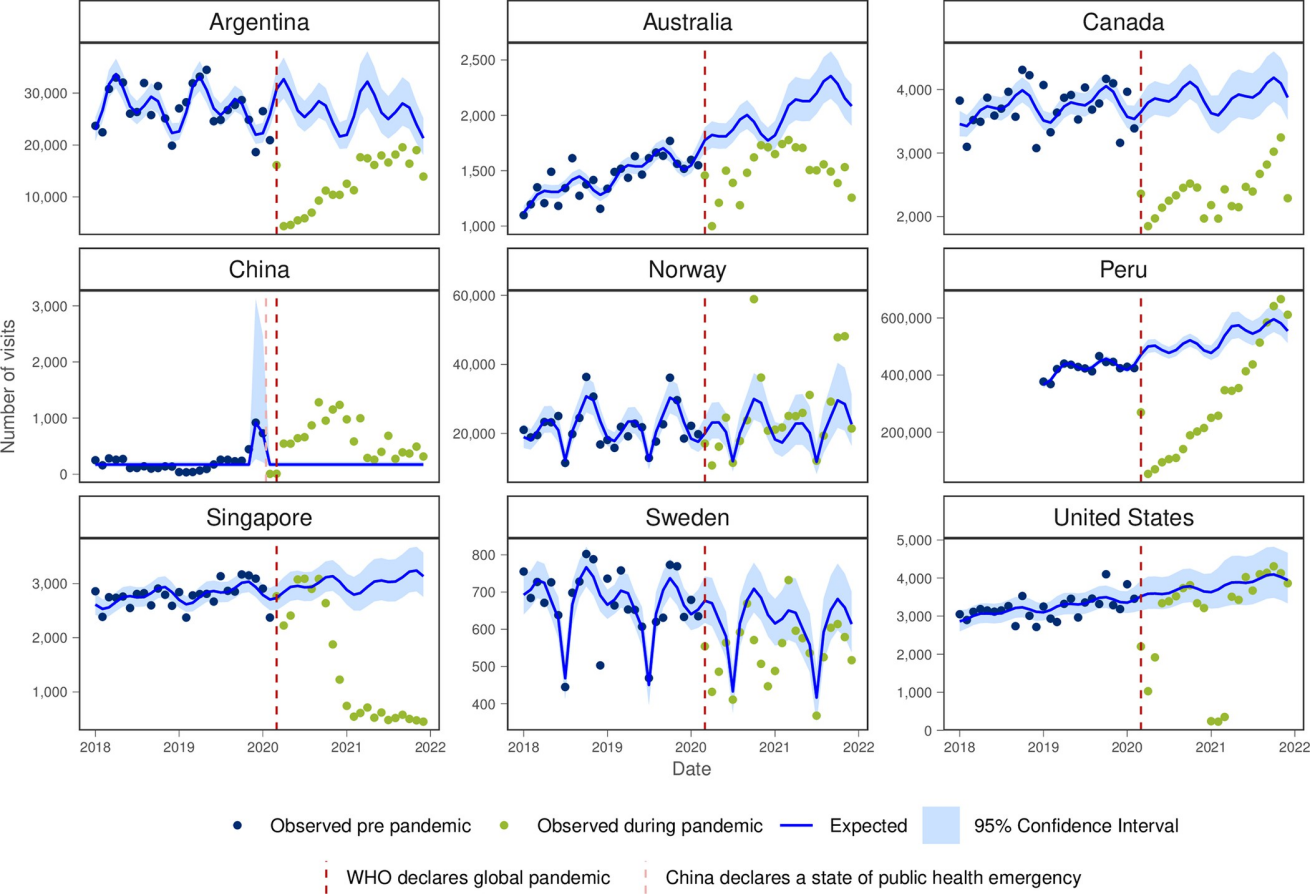
Number of observed and expected visits for preventative care in INTRePID countries 2018–2021.

Visits for preventive health exams were lower than expected for all countries except for Norway, the USA and at the end of 2021 for Peru (Fig 7). The monthly visit rate for preventive health exams decreased during the first year of the pandemic for Argentina, Canada, Peru and Sweden though recovered in the second year of the pandemic for Peru.

**Fig 8:**
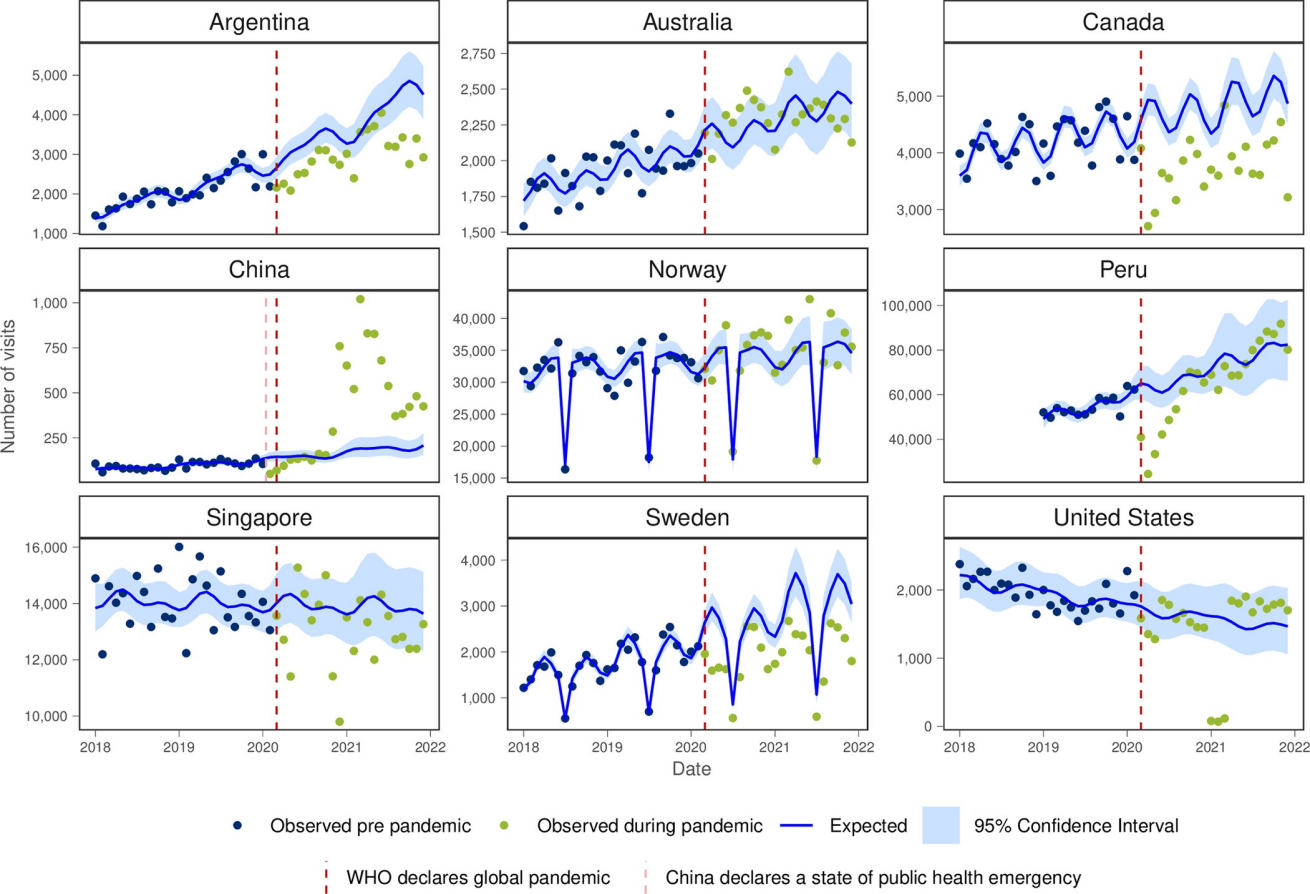
Number of observed and expected visits for diabetes in INTRePID countries 2018–2021.

**Fig 9:**
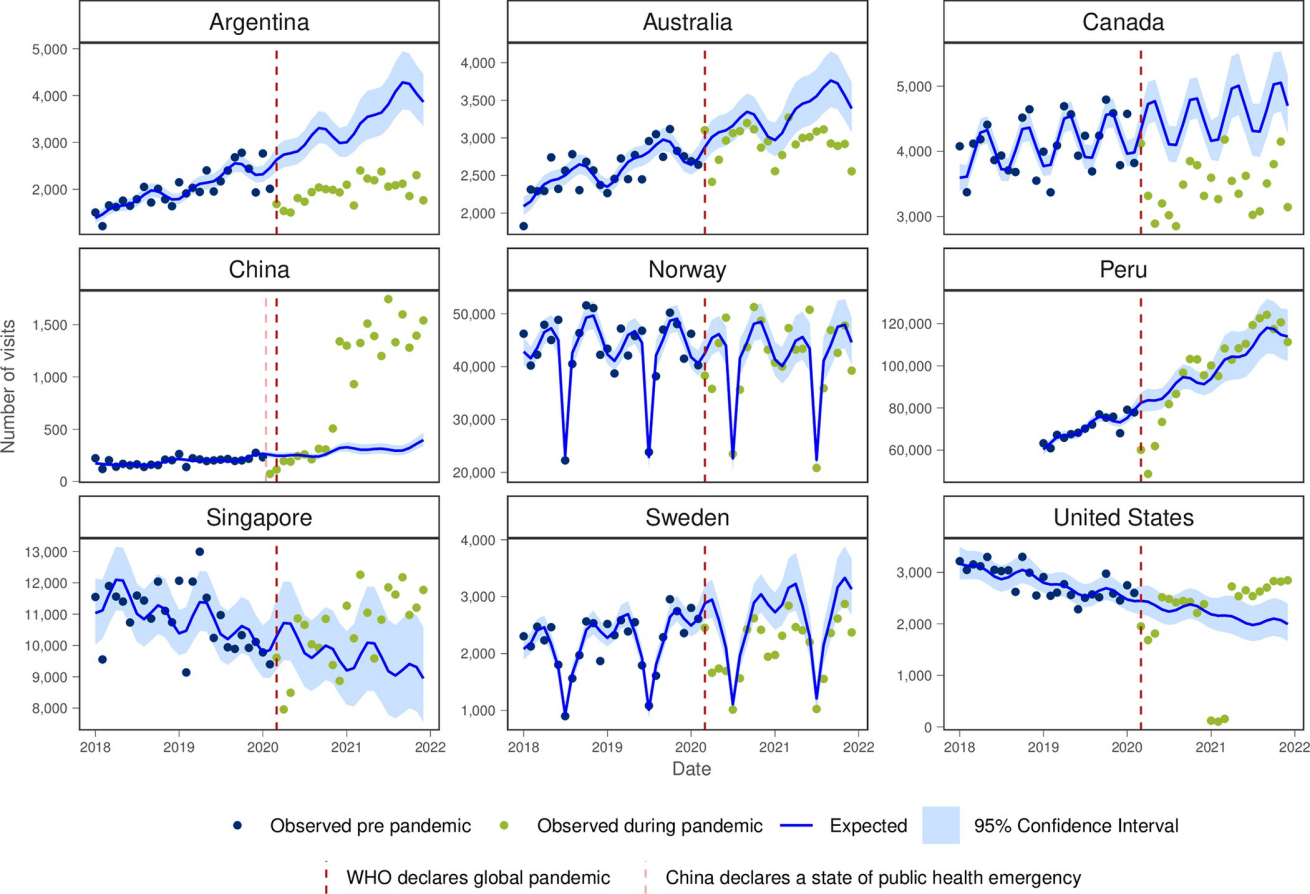
Number of observed and expected visits for hypertension in INTRePID countries 2018–2021.

**Fig 10:**
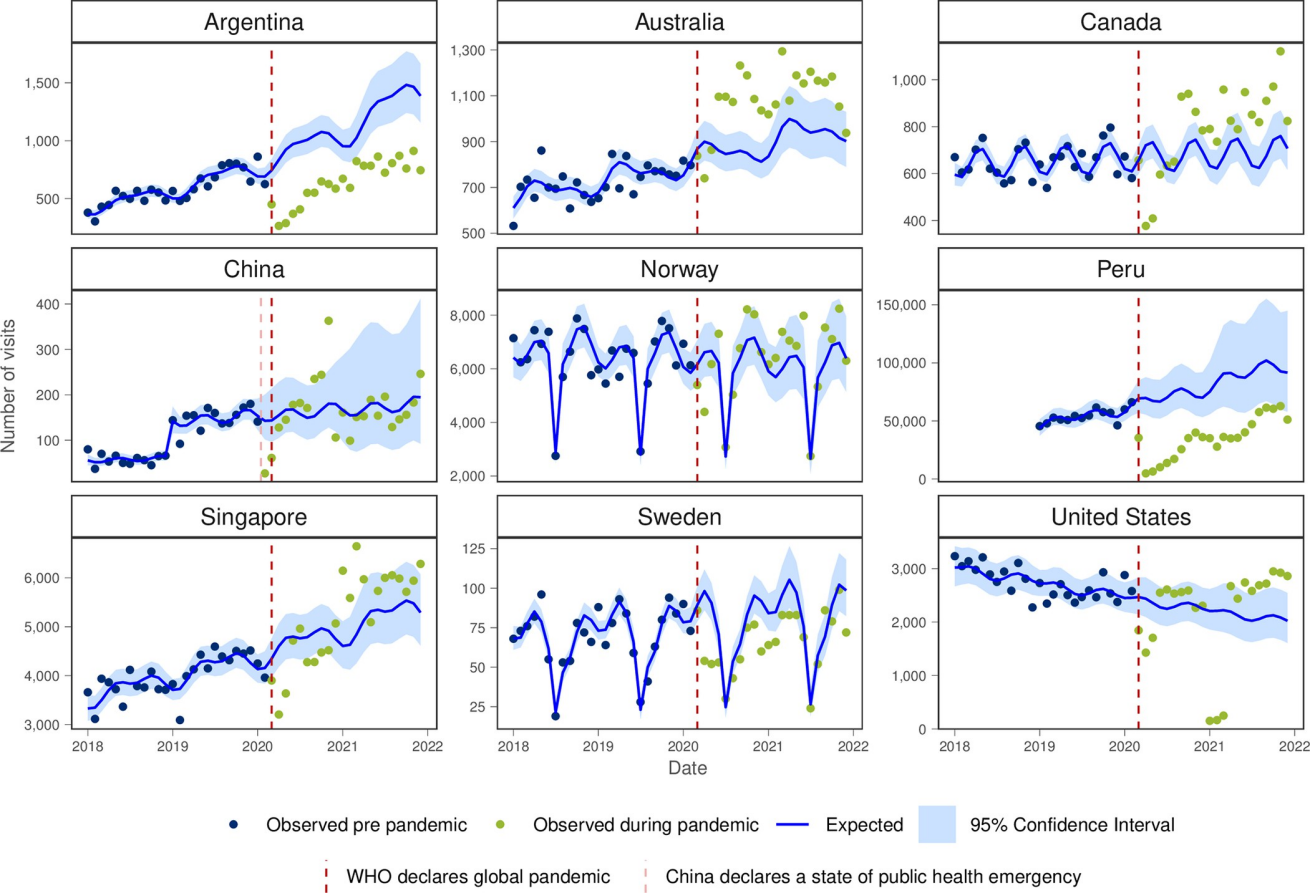
Number of observed and expected visits for hypercholesterolemia in INTRePID countries 2018–2021.

Argentina, Canada and Sweden had lower observed visits than expected for diabetes and hypertension (Figs 8 and 9) and monthly visit rates were lower in the first year of the pandemic in Canada, Singapore and the USA for both conditions (Table 1). For hypercholesterolemia, Australia and Canada had higher observed visits than expected, while Argentina and Peru showed lower visit volume than expected (Fig 10).

## Discussion

Through INTRePID we were able to examine changes to primary care visit volume and modality pre-and during the pandemic, and the most common reasons for visits to primary care in nine different countries. Although overall monthly visit rates decreased for many countries with the onset of the pandemic, they remained similar during the first and second year of the pandemic for all countries except Peru where visit volume increased. The slow upward trend in Peru was likely due to the prolonged lockdown and an initial shortage of healthcare workers. Peru began its lockdown in March 2020 and maintained a focused quarantine until October of the same year, which was considered one of the longest lockdowns worldwide [23]. Another contributing factor was that regular healthcare workers, especially those who were older or had comorbidities, spent extended periods away from work. Those who remained working were primarily reassigned to COVID-19 response efforts [24].

The use of virtual care seen in INTRePID countries in the first year of the pandemic appeared to be maintained in the second year albeit in most countries to a lesser degree. The persistence of virtual care reflects an evolution in healthcare delivery methods, with healthcare providers and patients recognizing the benefits of virtual visits. In the second year of the pandemic, a hybrid model of care emerged in many countries, where virtual visits complemented rather than replaced in-person visits.

The top 10 reasons for visits were similar in the inaugural INTRePID countries (Australia, Canada, China, Norway, Singapore, Sweden, USA) and consistent with a prior systematic review that found the most common reasons for visits to PCPs in developed countries was URTI, hypertension, depression or anxiety and back pain [4]. Our two new INTRePID countries bring developing South American countries to INTRePID. In them, our findings of a predominance of preventative care visits are consistent with prior reports [25], but our findings of a high frequency of gynecologic related visits highlight how primary care delivered in South American countries for contraceptive care differs from most developed countries. In Argentina and Peru, women typically go monthly to primary care clinics to receive birth control (such as oral or intramuscular contraceptives, or condoms), and if using intrauterine devices, surveillance by healthcare providers is performed every 6–12 months. Conversely, in the most affluent countries, women often receive yearly or longer prescriptions for oral contraceptives while intrauterine devices are self-monitored with replacements occurring most commonly at five years or longer. Our findings show that visits to PCPs for gynecological reasons in the USA were rare which suggests that women in the USA are more likely to see gynecologists for these issues. Another regional variation observed in Argentina, Peru and the USA was that overweight and obesity were frequent reasons for visits, which underscores the growing concern about this health condition and its impact on public health in these countries [26,27].

Common reasons for visits to primary care such as diabetes, hypertension, URTI and general health exams were consistently among the top reasons for visits across most countries in 2019. Notably, anxiety, depression, and COVID-19 became prominent reasons for visits during the pandemic period. The rise in mental health-related visits particularly driven by anxiety/depression, has been recently documented by our research group [2].

Furthermore, among the countries that used virtual care, anxiety/depression were amongst the most common reason for virtual visits. Although the increase in anxiety/depression seen in several of the INTRePID countries was not unexpected [28,29], it was most marked in Canada. However, the prevalence of mental health conditions in some countries are likely higher than reported here. Reasons for underreporting here are likely related to both cultural factors in terms of recognition and acceptance of mental health conditions and where mental health services are delivered in relation to our available data in the various primary care settings. In some cultures, people may report somatic rather than emotional symptoms [30,31]. In some countries it is possible that patients present to a psychiatrist or other mental health workers rather than to their PCP. For example, in Argentina, our data is limited to primary care centres and excludes information from public mental health centres where patients can attend without referrals. Additionally in Argentina, a pre-existing program for telephone access to mental health services was reinforced to meet pandemic demands and mental health professionals in clinics and general hospitals were among the first to implement virtual care early in the pandemic. Despite their advanced healthcare systems and technological infrastructure, our study found minimal uptake of virtual care during the pandemic in China and Singapore. It has been reported that virtual care adoption among healthcare professionals in China faces several challenges including infrastructure limitations, complex service processes, cost concerns, and variable popularity across regions [32]. In Singapore, virtual care was used during the pandemic by some hospitals and private general practitioners’ clinics to provide care for high-risk patients such as the elderly and children [33–35]. Some public primary care providers also used virtual care for their patients with chronic conditions [36–37]. However, in the primary care setting where our data from Singapore was from, virtual care was not widely used. Limited access to technology, combined with cultural norms and patient preferences for traditional healthcare interactions, likely contributed to the lower utilization of virtual care in Argentina [38].

Cough, cold or URTI were also among the top 10 reasons for virtual care in all countries that had virtual care except for the USA. This suggests that in most countries virtual visits were perceived as a safe and effective means of triaging and monitoring patients with respiratory tract infections, including COVID-19. In both Australia and Singapore, URTI was the most common reason for visit to PCPs in 2019 but, as with all INTRePID countries, the volume of visits for cough, cold, and URTIs dropped during the pandemic, possibly due in part to less occurrence of these diseases resulting from COVID-19 public health measures and policies adopted worldwide [39,40], and/or the creation of COVID-19 respiratory assessment clinics that were separate from regular PCPs offices.

The predictive analysis enables us to compare the observed trends with the expected trends across the six common reasons for visits that were studied. The notable surge in anxiety/ depression visits in Australia, Canada, Peru, and Singapore suggests a heightened demand for mental health services during this challenging period. Conversely, the significant decline in cough and cold visits across all countries reflects potential changes in healthcare-seeking behaviour and the effects of public health measures such as social distancing and mask-wearing.

The substantial decrease in preventative care visits in Argentina, Australia, Canada, Peru, Singapore, and Sweden raises concerns about the potential long-term implications for preventive health measures and disease management. Similarly, lower-than-expected diabetes and hypertension visits in certain regions highlight potential disruptions in chronic disease management, possibly due to access barriers or shifts in healthcare priorities during the pandemic.

While there have been systematic reviews comparing common reasons for visits to primary care and the impacts of COVID in multiple jurisdictions, our study stands out as the data was extracted locally but analyzed together centrally. This allows for better cross-country comparison using methods for aggregating data and analysis of data that were uniformly applied across countries.

Attempting to compare primary care visits between countries has challenges and limitations. First, different countries use different coding systems, and the timing of the adoption of different versions of the same coding system can vary both between countries and within a country. While SNOMED and ICD-10 have many detailed codes, other coding systems have far fewer codes to choose from. This means that coding systems with fewer code choices may group a broader range of conditions under the same code than those with more choices. To address this issue, for countries using ICD-10 and its variants, we limited codes to a letter and the first 2 digits (parent type categories of disease) as the later digits often refer to variations of the same disease. We also amalgamated codes for common diseases to gain a better view of specific conditions that may have multiple codes within one coding system. Despite these differences, we found that the most common reasons for visits were similar across developed and developing countries.

Second, because primary care clinic workforce and services vary among the INTRePID countries, it was not possible to distinguish visits specifically made to PCPs in Argentina, Peru, and Sweden. Therefore, our results for the top 10 most common conditions observed in those countries may be more reflective of primary care clinic activity than specific PCP activity.

Third, there were several country specific issues that may limit the interpretation of our results. In Argentina and partly in Peru, the text had to be manually coded to ICD-10 and inconsistencies in documenting virtual visits might have led to an underreporting of virtual visits in these countries. In Peru data from 2018 was not available. China had major policy changes (change in coding system in October 2020, restricting mental health visits in favour of chronic disease visits in 2021) that limited our ability to interpret the 2021 data.

Last, our data may not be fully representative of an entire country as availability of data varied by country. Nevertheless, our study´s strength lies in its large number of patient visits across multiple countries in several different continents. This approach provides a glimpse of a global picture and may be less biased than survey-based international comparative studies that assess particular respondents within a region. Additionally, the involvement of primary care physicians and investigators in each country to provide and confirm face validity of the country-specific findings leads us to believe that what is represented here is reasonably accurate and reflective of the trends going on in the individual countries.

The COVID-19 pandemic resulted in changes in how primary care is delivered, with the changes that were set in motion continuing post pandemic. Changes in primary care delivery during the pandemic offer insight into potential gaps in care and preventive services resulting from the pandemic. While virtual visits can provide short-term care for chronic diseases, regular or occasional in-person visits are still necessary for physical examination and laboratory testing in the long term. The suspending of cancer screening programs at the beginning of the pandemic [41] likely contributed to the lack of preventive care visits in Canada. The evidence for virtual care leading to more emergency room visits is conflicting [42,43]. Whether these temporary pauses in care or switch to virtual care result in lower quality of care and negative long-term outcomes has yet to be determined. The impacts of the pandemic and the switch to virtual care appear to be sustained and provide insights into what the ‘new normal’ of primary care may be in the future. Moving forward, INTRePID plans to continue to investigate the sustainability of virtual care post-pandemic to gain deeper insights into the evolving primary care landscape. We aim to expand our research on virtual care trends for prevalent conditions, with a broader scope that includes recently joined countries in our future analysis.

## Supporting information

S1 Table Anxiety and depression diagnosis codes. (PDF)

S2 Table Cough, cold and acute upper respiratory tract infection diagnosis codes. (PDF)

S3 Table Preventative care/general health check diagnosis codes. (PDF)

S4 Table Diabetes diagnosis codes. (PDF)

S5 Table Hypercholesterolemia diagnosis codes. (PDF)

S6 Table Hypertension diagnosis codes. (PDF)

S7 Table Top 10 reasons for total visits to primary care in 2019. (PDF)

S8 Table Top 10 reasons for in-person visits to primary care in 2020. (PDF)

S9 Table Top 10 reasons for virtual visits to primary care in 2020. (PDF)

S1 Data Data sources. (PDF)

S2 Data Monthly visits by modality of care. (PDF)

S3 Data Monthly visits for common conditions. (PDF)
