# Supplementary material for: Changes in reasons for visits to primary care after the start of the COVID-19 pandemic: An international comparative study by the International Consortium of Primary Care Big Data Researchers (INTRePID)
Source: PLOS Glob Public Health. 2024 Aug 22;4(8):e0003406. doi: 10.1371/journal.pgph.0003406 (PMC11341054; doi:10.1371/journal.pgph.0003406)
Supplement: S1 Table — (PDF) [file pgph.0003406.s001.pdf]

S1 Table. Anxiety and depression diagnosis codes

System:	ICD-10/ICD-10 CM/ICD-10 AM	
**Code**	**Description**	**Found**
F32	Depressive episode	X
F32.0	Mild depressive episode	X
F32.1	Moderate depressive episode	X
F32.2	Severe depressive episode without psychotic symptoms	X
F32.3	Severe depressive episode with psychotic symptoms	X
F32.4	Major depressive disorder, single episode, in partial remission	X
F32.8	Other depressive episodes	X
F32.89	Other specified depressive episodes	X
F32.9	Depressive episode, unspecified	X
F32.A	Depression, unspecified	X
F33	Recurrent depressive disorder	X
F33.0	Recurrent depressive disorder, current episode mild	X
F33.1	Recurrent depressive disorder, current episode moderate	X
F33.2	Recurrent depressive disorder, current episode severe without psychotic symptoms	X
F33.3	Recurrent depressive disorder, current episode severe with psychotic symptoms	X
F33.41	Major depressive disorder, recurrent, in partial remission	X
F33.8	Other recurrent depressive disorders	X
F33.9	Recurrent depressive disorder, unspecified	X
F34	Persistent mood affective disorders	X
F34.1	Dysthymic disorder	X
F34.8	Other persistent mood affective disorders	X
F34.81	Disruptive mood dysregulation disorder	X
F34.89	Other specified persistent mood disorders	X
F34.9	Persistent mood affective disorder, unspecified	X
F39	Unspecified mood [affective] disorder	X
F40	Phobic anxiety disorders	X
F40.0	Agoraphobia	X
F40.00	Agoraphobia, unspecified	X
F40.01	Agoraphobia with panic disorder	X
F40.02	Agoraphobia without panic disorder	X
F40.1	Social phobias	X
F40.10	Social phobia, unspecified	
F40.11	Social phobia, generalized	X
F40.2	Specific (isolated) phobias	X
F40.21	Animal type phobia	
F40.210	Arachnophobia	
F40.218	Other animal type phobia	X
F40.22	Natural environment type phobia	
F40.220	Fear of thunderstorms	
F40.228	Other natural environment type phobia	X
F40.23	Blood, injection, injury type phobia	
F40.230	Fear of blood	
F40.231	Fear of injections and transfusions	X
F40.232	Fear of other medical care	X
F40.233	Fear of injury	X
F40.24	Situational type phobia	X
F40.240	Claustrophobia	X

S1 Table. Anxiety and depression diagnosis codes (continued)

System:	ICD-10/ICD-10 CM/ICD-10 AM	
Code	Description	Found
F40.240	Claustrophobia	X
F40.241	Acrophobia	X
F40.242	Fear of bridges	
F40.243	Fear of flying	X
F40.248	Other situational type phobia	X
F40.29	Other specified phobia	
F40.290	Androphobia	
F40.291	Gynophobia	
F40.298	Other specified phobia	X
F40.8	Other phobic anxiety disorders	X
F40.9	Phobic anxiety disorder, unspecified	X
F41	Other anxiety disorders	X
F41.0	Panic disorder episodic paroxysmal anxiety	X
F41.1	Generalized anxiety disorder	X
F41.2	Mixed anxiety and depressive disorder	X
F41.3	Other mixed anxiety disorders	X
F41.8	Other specified anxiety disorders	X
F41.9	Anxiety disorder, unspecified	X
F42	Obsessive-compulsive disorder	X
F42.0	Predominantly obsessional thoughts or ruminations	X
F42.1	Predominantly compulsive acts [obsessional rituals]	X
F42.2	Mixed obsessional thoughts and acts	X
F42.8	Other obsessive-compulsive disorder	X
F42.9	Obsessive-compulsive disorder, unspecified	X

System:	SNOMED CT	
Code	Description	Found
832007	Moderate major depression	
5874002	Anticipatory anxiety, severe	
10358001	Heightened global phobia	
11458009	Anticipatory anxiety, mild	
21897009	Generalised anxiety disorder	X
25501002	Social phobia	
35429005	Anticipatory anxiety	
35489007	Depression	X
36646009	Anticipatory anxiety, moderate	
37868008	Anxiety disorder of adolescence	
38237000	Feeling anxious	
38617005	Dental phobia	
41006004	Depressed, anxious, tearful, miserable	
46206005	Mood disorder	X
48694002	Anxiety	X
52039009	Phobia	
53467004	Anxiety disorder of childhood	
54307006	Zoophobia	
54587008	Simple phobia	
61387006	Moderate anxiety	
62351001	Generalized social phobia	
65673007	Anxiety disorder	

S1 Table. Anxiety and depression diagnosis codes (continued)

System:<br>Code	SNOMED CT<br>Description	Found
66344007	Recurrent major depression	
69479009	Anxiety hyperventilation	
70691001	Agoraphobia	
70997004	Mild anxiety	
73867007	Severe major depression with psychotic features	
79842004	Stuporous depression	
83458005	Agitated depression	
111487009	Dream anxiety disorder	
126943008	Separation anxiety	
154887003	Other phobias	
154919005	Moderate depression	
154965008	Mild depression	
154966009	Moderate depression	
154967000	Severe depression	
154970001	Recurrent depression	
191616006	Recurrent depression	
191703000	Anxiety disorder	
191708009	Chronic anxiety	
191709001	Recurrent anxiety	
191721002	Phobia unspecified	
191724005	Social phobia, fear of eating in public	
191725006	Social phobia, fear of public speaking	
191730005	Cancer phobia	
191731009	Dental phobia	
191736004	Obsessive-compulsive disorder	X
192080009	Chronic depression	
192395002	Social phobia	
192396001	Specific (isolated) phobias (& [acrophobia] or [animal] or [claustrophobia] or [simple phobia])	
192611004	Childhood phobic anxiety disorder	
197480006	Anxiety disorder	X
198288003	Anxiety state	
207363009	Anxiety neurosis	
225624000	Panic attack	X
231500002	Masked depression	
231501003	Needle phobia	
231504006	Mixed anxiety and depressive disorder	X
231506008	Anxiety hysteria	
247854002	Flying phobia	
255339005	Depression - motion	
274948002	Endogenous depression - recurrent	
280947008	Examination phobia	
300706003	Endogenous depression	
300894000	Parental anxiety	
300895004	Anxiety attack	
300930008	Cancer phobia	
307526003	Other phobias	
310495003	Mild depression	
310496002	Moderate depression	
321717001	Involutional depression (disorder)	

S1 Table. Anxiety and depression diagnosis codes (continued)

System: Code	SNOMED CT Description	Found
366979004	Depressed mood	X
370143000	Major depressive disorder	X
370567003	Noise phobia	
386808001	Phobia	
390717003	Mild depression	
392721003	Symptoms of depression	
413973005	Depression interim review	
450714000	Severe major depression	
473126001	Suspected depressive disorder	
712823008	Acute depression	
718636001	Minimal depression	
719593009	Moderately severe depression	
720455008	Minimal major depression	
768835002	Depression care management	
286711000000107	Counselling for depression	
16265701000119107	Illness anxiety disorder	

System: Code	ICPC-2 Description	Found
P01	Feeling anxious/nervous/tense	X
P03	Feeling depressed	X
P74	Anxiety disorder/anxiety state	X
P76	Depressive disorder	X
P79	Phobia/compulsive disorder	X

System: Code	OHIP Description	Found
300	Anxiety/obsessive compulsive neurosis	X
311	Depressive or other non-psychotic disorders	X
