# Supplementary material for: Changes in reasons for visits to primary care after the start of the COVID-19 pandemic: An international comparative study by the International Consortium of Primary Care Big Data Researchers (INTRePID)
Source: PLOS Glob Public Health. 2024 Aug 22;4(8):e0003406. doi: 10.1371/journal.pgph.0003406 (PMC11341054; doi:10.1371/journal.pgph.0003406)
Supplement: S2 Table — (PDF) [file pgph.0003406.s002.pdf]

S2 Table. Cough and cold acute upper respiratory tract infection diagnosis codes

System:	ICD-10/ICD-10 CM/ICD-10 AM	
**Code**	**Description**	**Found**
J00	Acute nasopharyngitis [common cold]	X
J06.8	Other acute upper respiratory infections of multiple sites	X
J06.9	Acute upper respiratory infection, unspecified	X
R05	Cough	X
R05.1	Acute cough	X
R05.2	Subacute cough	X
R05.9	Cough, unspecified	X

System:	SNOMED CT	
**Code**	**Description**	**Found**
49727002	Cough	X
54150009	Upper respiratory tract infection	X
54398005	Upper respiratory tract infection	
82272006	Common cold	X
155516001	Acute upper respiratory infection	
158383001	Cough	
195704001	Other acute upper respiratory infections	
195705000	Acute upper respiratory infection	
196195006	Acute upper respiratory infection	
207066005	Cough	
232342002	Common cold	
263731006	Coughing	
281794004	Viral upper respiratory tract infection	
445241004	Postviral cough	X

System:	ICPC-2	
**Code**	**Description**	**Found**
R05	Cough	X
R74	Upper respiratory infection acute	X

System:	OHIP	
**Code**	**Description**	**Found**
460	Cold, common	X
786	Cough	X
