# Supplementary material for: Changes in reasons for visits to primary care after the start of the COVID-19 pandemic: An international comparative study by the International Consortium of Primary Care Big Data Researchers (INTRePID)
Source: PLOS Glob Public Health. 2024 Aug 22;4(8):e0003406. doi: 10.1371/journal.pgph.0003406 (PMC11341054; doi:10.1371/journal.pgph.0003406)
Supplement: S3 Table — (PDF) [file pgph.0003406.s003.pdf]

S3 Table. Preventative care/general health check diagnosis codes diagnosis codes

System:	ICD-10/ICD-10 CM/ICD-10 AM	
**Code**	**Description**	**Found**
Z00	Encounter for general examination without complaint, suspected or reported diagnosis	X
Z00.0	Encounter for general adult medical examination	X
Z00.00	Encounter for general adult medical examination without abnormal findings	X
Z00.01	Encounter for general adult medical examination with abnormal findings	X
Z00.1	Encounter for newborn, infant and child health examinations	X
Z00.11	Newborn health examination	
Z00.110	Health examination for newborn under 8 days old	X
Z00.111	Health examination for newborn 8 to 28 days old	X
Z00.12	Encounter for routine child health examination	X
Z00.121	Encounter for routine child health examination with abnormal findings	X
Z00.129	Encounter for routine child health examination without abnormal findings	X
Z00.2	Encounter for examination for period of rapid growth in childhood	X
Z00.3	Encounter for examination for adolescent development state	X
Z00.8	Other general examinations	X
Z01.4	Gynaecological examination (general)(routine)	X
Z01.41	Encounter for routine gynecological examination	X
Z01.411	Encounter for gynecological examination (general) (routine) with abnormal findings	X
Z01.419	Encounter for gynecological examination (general) (routine) without abnormal findings	X
Z02.0	Encounter for examination for admission to educational institution	X
Z02.2	Encounter for examination for admission to residential institution	X
Z02.3	Encounter for examination for recruitment to armed forces	X
Z02.5	Encounter for examination for participation in sport	X
Z03	Encounter for medical observation for suspected diseases and conditions ruled out	X
Z04.89	Encounter for examination and observation for other specified reasons	X
Z04.9	Encounter for examination and observation for unspecified reason	X
Z10	Routine general health check-up of defined subpopulation	X
Z10.1	Routine general health check-up of inhabitants of institutions	X
Z10.2	Routine general health check-up of armed forces	
Z10.3	Routine general health check-up of sports teams	X
Z10.8	Routine general health check-up of other defined subpopulations	X

System:	SNOMED CT	
**Code**	**Description**	**Found**
102509001	Healthy adolescent	
102512003	Healthy adult	
134186004	Health check, over 75 yr	X
170114005	Child examination-6 weeks	X
185349003	Check up	X
268481000	Child health care	
268565007	Adult health examination	X
275926002	Screening health check	X
225885004	Health assessment	X
281032009	Women's health check	

S3 Table. Preventative care/general health check diagnosis codes diagnosis codes (continued)

System:	SNOMED CT	
**Code**	**Description**	**Found**
316552009	Routine general health check-up of sports teams	
316559000	Routine general health check-up of inhabitants of institutions	
316560005	Routine general health check-up of armed forces	
316722004	Routine general health check-up of other defined subpopulation	

System:	ICPC-2	
**Code**	**Description**	**Found**
A98	Health maintenance/prevention	X

System:	OHIP	
**Code**	**Description**	**Found**
916	Well baby care	X
917	Annual health examination adolescent/adult well vision care	X
