# Supplementary material for: Changes in reasons for visits to primary care after the start of the COVID-19 pandemic: An international comparative study by the International Consortium of Primary Care Big Data Researchers (INTRePID)
Source: PLOS Glob Public Health. 2024 Aug 22;4(8):e0003406. doi: 10.1371/journal.pgph.0003406 (PMC11341054; doi:10.1371/journal.pgph.0003406)
Supplement: S4 Table — (PDF) [file pgph.0003406.s004.pdf]

S4 Table. Diabetes diagnosis codes

System:	ICD-10/ICD-10 CM/ICD-10 AM	
**Code**	**Description**	**Found**
E10	Type 1 diabetes mellitus	X
E10.0	Type 1 diabetes mellitus, with acidosis	X
E10.1	Type 1 diabetes mellitus, with coma	X
E10.10	Type 1 diabetes mellitus with ketoacidosis without coma	X
E10.11	Type 1 diabetes mellitus with ketoacidosis with coma	X
E10.2	Type 1 diabetes mellitus, with renal complications	X
E10.21	Type 1 diabetes mellitus with diabetic nephropathy	X
E10.22	Type 1 diabetes mellitus with diabetic chronic kidney disease	X
E10.29	Type 1 diabetes mellitus with other diabetic kidney complication	X
E10.3	Type 1 diabetes mellitus, with ophthalmic complications(h36.0*)	X
E10.31	Type 1 diabetes mellitus with unspecified diabetic retinopathy	
E10.311	Type 1 diabetes mellitus with unspecified diabetic retinopathy with macular edema	X
E10.319	Type 1 diabetes mellitus with unspecified diabetic retinopathy without macular edema	X
E10.32	Type 1 diabetes mellitus with mild nonproliferative diabetic retinopathy	
E10.321	Type 1 diabetes mellitus with mild nonproliferative diabetic retinopathy with macular edema	
E10.3211	Type 1 diabetes mellitus with mild nonproliferative diabetic retinopathy with macular edema, right eye	
E10.3212	Type 1 diabetes mellitus with mild nonproliferative diabetic retinopathy with macular edema, left eye	
E10.3213	Type 1 diabetes mellitus with mild nonproliferative diabetic retinopathy with macular edema, bilateral	X
E10.3219	Type 1 diabetes mellitus with mild nonproliferative diabetic retinopathy with macular edema, unspecified eye	X
E10.329	Type 1 diabetes mellitus with mild nonproliferative diabetic retinopathy without macular edema	
E10.3291	Type 1 diabetes mellitus with mild nonproliferative diabetic retinopathy without macular edema, right eye	X
E10.3292	Type 1 diabetes mellitus with mild nonproliferative diabetic retinopathy without macular edema, left eye	X
E10.3293	Type 1 diabetes mellitus with mild nonproliferative diabetic retinopathy without macular edema, bilateral	X
E10.3299	Type 1 diabetes mellitus with mild nonproliferative diabetic retinopathy without macular edema, unspecified eye	X
E10.33	Type 1 diabetes mellitus with moderate nonproliferative diabetic retinopathy	
E10.331	Type 1 diabetes mellitus with moderate nonproliferative diabetic retinopathy with macular edema	
E10.3311	Type 1 diabetes mellitus with moderate nonproliferative diabetic retinopathy with macular edema, right eye	X
E10.3312	Type 1 diabetes mellitus with moderate nonproliferative diabetic retinopathy with macular edema, left eye	X
E10.3313	Type 1 diabetes mellitus with moderate nonproliferative diabetic retinopathy with macular edema, bilateral	
E10.3319	Type 1 diabetes mellitus with moderate nonproliferative diabetic retinopathy with macular edema, unspecified eye	X
E10.339	Type 1 diabetes mellitus with moderate nonproliferative diabetic retinopathy without macular edema	

S4 Table. Diabetes diagnosis codes (continued)

System: Code	ICD-10/ICD-10 CM/ICD-10 AM Description	Found
E10.3391	Type 1 diabetes mellitus with moderate nonproliferative diabetic retinopathy without macular edema, right eye	
E10.3392	Type 1 diabetes mellitus with moderate nonproliferative diabetic retinopathy without macular edema, left eye	
E10.3393	Type 1 diabetes mellitus with moderate nonproliferative diabetic retinopathy without macular edema, bilateral	X
E10.3399	Type 1 diabetes mellitus with moderate nonproliferative diabetic retinopathy without macular edema, unspecified eye	X
E10.34	Type 1 diabetes mellitus with severe nonproliferative diabetic retinopathy	
E10.341	Type 1 diabetes mellitus with severe nonproliferative diabetic retinopathy with macular edema	
E10.3411	Type 1 diabetes mellitus with severe nonproliferative diabetic retinopathy with macular edema, right eye	
E10.3412	Type 1 diabetes mellitus with severe nonproliferative diabetic retinopathy with macular edema, left eye	X
E10.3413	Type 1 diabetes mellitus with severe nonproliferative diabetic retinopathy with macular edema, bilateral	
E10.3419	Type 1 diabetes mellitus with severe nonproliferative diabetic retinopathy with macular edema, unspecified eye	X
E10.349	Type 1 diabetes mellitus with severe nonproliferative diabetic retinopathy without macular edema	
E10.3491	Type 1 diabetes mellitus with severe nonproliferative diabetic retinopathy without macular edema, right eye	
E10.3492	Type 1 diabetes mellitus with severe nonproliferative diabetic retinopathy without macular edema, left eye	
E10.339	Type 1 diabetes mellitus with moderate nonproliferative diabetic retinopathy without macular edema	
E10.3391	Type 1 diabetes mellitus with moderate nonproliferative diabetic retinopathy without macular edema, right eye	
E10.3392	Type 1 diabetes mellitus with moderate nonproliferative diabetic retinopathy without macular edema, left eye	
E10.3393	Type 1 diabetes mellitus with moderate nonproliferative diabetic retinopathy without macular edema, bilateral	
E10.3399	Type 1 diabetes mellitus with moderate nonproliferative diabetic retinopathy without macular edema, unspecified eye	
E10.34	Type 1 diabetes mellitus with severe nonproliferative diabetic retinopathy	
E10.341	Type 1 diabetes mellitus with severe nonproliferative diabetic retinopathy with macular edema	
E10.3411	Type 1 diabetes mellitus with severe nonproliferative diabetic retinopathy with macular edema, right eye	
E10.3412	Type 1 diabetes mellitus with severe nonproliferative diabetic retinopathy with macular edema, left eye	
E10.3413	Type 1 diabetes mellitus with severe nonproliferative diabetic retinopathy with macular edema, bilateral	
E10.3419	Type 1 diabetes mellitus with severe nonproliferative diabetic retinopathy with macular edema, unspecified eye	
E10.349	Type 1 diabetes mellitus with severe nonproliferative diabetic retinopathy without macular edema	
E10.3491	Type 1 diabetes mellitus with severe nonproliferative diabetic retinopathy without macular edema, right eye	

S4 Table. Diabetes diagnosis codes (continued)

System: Code	ICD-10/ICD-10 CM/ICD-10 AM Description	Found
E10.3492	Type 1 diabetes mellitus with severe nonproliferative diabetic retinopathy without macular edema, left eye	
E10.3493	Type 1 diabetes mellitus with severe nonproliferative diabetic retinopathy without macular edema, bilateral	X
E10.3499	Type 1 diabetes mellitus with severe nonproliferative diabetic retinopathy without macular edema, unspecified eye	
E10.35	Type 1 diabetes mellitus with proliferative diabetic retinopathy	
E10.351	Type 1 diabetes mellitus with proliferative diabetic retinopathy with macular edema	
E10.3511	Type 1 diabetes mellitus with proliferative diabetic retinopathy with macular edema, right eye	
E10.3512	Type 1 diabetes mellitus with proliferative diabetic retinopathy with macular edema, left eye	
E10.3513	Type 1 diabetes mellitus with proliferative diabetic retinopathy with macular edema, bilateral	X
E10.3519	Type 1 diabetes mellitus with proliferative diabetic retinopathy with macular edema, unspecified eye	
E10.352	Type 1 diabetes mellitus with proliferative diabetic retinopathy with traction retinal detachment involving the macula	
E10.3521	Type 1 diabetes mellitus with proliferative diabetic retinopathy with traction retinal detachment involving the macula, right eye	
E10.3522	Type 1 diabetes mellitus with proliferative diabetic retinopathy with traction retinal detachment involving the macula, left eye	
E10.3523	Type 1 diabetes mellitus with proliferative diabetic retinopathy with traction retinal detachment involving the macula, bilateral	
E10.3529	Type 1 diabetes mellitus with proliferative diabetic retinopathy with traction retinal detachment involving the macula, unspecified eye	
E10.353	Type 1 diabetes mellitus with proliferative diabetic retinopathy with traction retinal detachment not involving the macula	
E10.3531	Type 1 diabetes mellitus with proliferative diabetic retinopathy with traction retinal detachment not involving the macula, right eye	
E10.3532	Type 1 diabetes mellitus with proliferative diabetic retinopathy with traction retinal detachment not involving the macula, left eye	
E10.3533	Type 1 diabetes mellitus with proliferative diabetic retinopathy with traction retinal detachment not involving the macula, bilateral	
E10.3539	Type 1 diabetes mellitus with proliferative diabetic retinopathy with traction retinal detachment not involving the macula, unspecified eye	
E10.354	Type 1 diabetes mellitus with proliferative diabetic retinopathy with combined traction retinal detachment and rhegmatogenous retinal detachment	
E10.3541	Type 1 diabetes mellitus with proliferative diabetic retinopathy with combined traction retinal detachment and rhegmatogenous, right eye	
E10.3542	Type 1 diabetes mellitus with proliferative diabetic retinopathy with combined traction retinal detachment and rhegmatogenous, left eye	
E10.3543	Type 1 diabetes mellitus with proliferative diabetic retinopathy with combined traction retinal detachment and rhegmatogenous, bilateral	
E10.3549	Type 1 diabetes mellitus with proliferative diabetic retinopathy with combined traction retinal detachment and rhegmatogenous, unspecified eye	
E10.355	Type 1 diabetes mellitus with stable proliferative diabetic retinopathy	

S4 Table. Diabetes diagnosis codes (continued)

System: Code	ICD-10/ICD-10 CM/ICD-10 AM Description	Found
E10.3551	Type 1 diabetes mellitus with stable proliferative diabetic retinopathy, right eye	
E10.3552	Type 1 diabetes mellitus with stable proliferative diabetic retinopathy, left eye	
E10.3553	Type 1 diabetes mellitus with stable proliferative diabetic retinopathy, bilateral	X
E10.3559	Type 1 diabetes mellitus with stable proliferative diabetic retinopathy, unspecified eye	X
E10.359	Type 1 diabetes mellitus with proliferative diabetic retinopathy without macular edema	X
E10.3591	Type 1 diabetes mellitus with proliferative diabetic retinopathy without macular edema, right eye	X
E10.3592	Type 1 diabetes mellitus with proliferative diabetic retinopathy without macular edema, left eye	
E10.3593	Type 1 diabetes mellitus with proliferative diabetic retinopathy without macular edema, bilateral	X
E10.3599	Type 1 diabetes mellitus with proliferative diabetic retinopathy without macular edema, unspecified eye	X
E10.36	Type 1 diabetes mellitus with diabetic cataract	X
E10.37	Type 1 diabetes mellitus with diabetic macular edema, resolved following treatment	
E10.37X1	Type 1 diabetes mellitus with diabetic macular edema, resolved following treatment, right eye	
E10.37X2	Type 1 diabetes mellitus with diabetic macular edema, resolved following treatment, left eye	
E10.37X3	Type 1 diabetes mellitus with diabetic macular edema, resolved following treatment, bilateral	
E10.37X9	Type 1 diabetes mellitus with diabetic macular edema, resolved following treatment, unspecified eye	
E10.39	Type 1 diabetes mellitus with other diabetic ophthalmic complication	X
E10.4	Type 1 diabetes mellitus, with neurological complications	X
E10.40	Type 1 diabetes mellitus with diabetic neuropathy, unspecified	X
E10.41	Type 1 diabetes mellitus with diabetic mononeuropathy	X
E10.42	Type 1 diabetes mellitus with diabetic polyneuropathy	X
E10.43	Type 1 diabetes mellitus with diabetic autonomic (poly)neuropathy	X
E10.44	Type 1 diabetes mellitus with diabetic amyotrophy	X
E10.49	Type 1 diabetes mellitus with other diabetic neurological complication	X
E10.5	Type 1 diabetes mellitus, with peripheral circulatory complications	X
E10.51	Type 1 diabetes mellitus with diabetic peripheral angiopathy without gangrene	X
E10.52	Type 1 diabetes mellitus with diabetic peripheral angiopathy with gangrene	
E10.59	Type 1 diabetes mellitus with other circulatory complications	X
E10.6	Type 1 diabetes mellitus, with other specified complications	X
E10.61	Type 1 diabetes mellitus with diabetic arthropathy	
E10.610	Type 1 diabetes mellitus with diabetic neuropathic arthropathy	X
E10.618	Type 1 diabetes mellitus with other diabetic arthropathy	
E10.62	Type 1 diabetes mellitus with skin complications	
E10.620	Type 1 diabetes mellitus with diabetic dermatitis	
E10.621	Type 1 diabetes mellitus with foot ulcer	X
E10.622	Type 1 diabetes mellitus with other skin ulcer	

S4 Table. Diabetes diagnosis codes (continued)

System:	ICD-10/ICD-10 CM/ICD-10 AM	
Code	Description	Found
E10.628	Type 1 diabetes mellitus with other skin complications	X
E10.63	Type 1 diabetes mellitus with oral complications	
E10.630	Type 1 diabetes mellitus with periodontal disease	
E10.638	Type 1 diabetes mellitus with other oral complications	
E10.64	Type 1 diabetes mellitus with hypoglycemia	
E10.641	Type 1 diabetes mellitus with hypoglycemia with coma	X
E10.649	Type 1 diabetes mellitus with hypoglycemia without coma	X
E10.65	Type 1 diabetes mellitus with hyperglycemia	X
E10.69	Type 1 diabetes mellitus with other specified complication	X
E10.7	Type 1 diabetes mellitus, with multiple complications	X
E10.8	Type 1 diabetes mellitus, with unspecified complications	X
E10.9	Type 1 diabetes mellitus, without complications	X
E11	Type 2 diabetes mellitus	X
E11.0	Type 2 diabetes mellitus, with coma	X
E11.00	Type 2 diabetes mellitus with hyperosmolarity without nonketotic hyperglycemic-hyperosmolar coma (nkhhc)	X
E11.01	Type 2 diabetes mellitus with hyperosmolarity with coma	X
E11.1	Type 2 diabetes mellitus , with acidosis	X
E11.10	Type 2 diabetes mellitus with ketoacidosis without coma	X
E11.11	Type 2 diabetes mellitus with ketoacidosis with coma	X
E11.2	Type 2 diabetes mellitus, with renal complications	X
E11.21	Type 2 diabetes mellitus with diabetic nephropathy	X
E11.22	Type 2 diabetes mellitus with diabetic chronic kidney disease	X
E11.29	Type 2 diabetes mellitus with other diabetic kidney complication	X
E11.3	Type 2 diabetes mellitus, with ophthalmic complications	X
E11.31	Type 2 diabetes mellitus with unspecified diabetic retinopathy	X
E11.311	Type 2 diabetes mellitus with unspecified diabetic retinopathy with macular edema	X
E11.319	Type 2 diabetes mellitus with unspecified diabetic retinopathy without macular edema	X
E11.32	Type 2 diabetes mellitus with mild nonproliferative diabetic retinopathy	
E11.321	Type 2 diabetes mellitus with mild nonproliferative diabetic retinopathy with macular edema	X
E11.3211	Type 2 diabetes mellitus with mild nonproliferative diabetic retinopathy with macular edema, right eye	X
E11.3212	Type 2 diabetes mellitus with mild nonproliferative diabetic retinopathy with macular edema, left eye	X
E11.3213	Type 2 diabetes mellitus with mild nonproliferative diabetic retinopathy with macular edema, bilateral	X
E11.3219	Type 2 diabetes mellitus with mild nonproliferative diabetic retinopathy with macular edema, unspecified eye	X
E11.329	Type 2 diabetes mellitus with mild nonproliferative diabetic retinopathy without macular edema	X
E11.3291	Type 2 diabetes mellitus with mild nonproliferative diabetic retinopathy without macular edema, right eye	X
E11.3292	Type 2 diabetes mellitus with mild nonproliferative diabetic retinopathy without macular edema, left eye	X
E11.3293	Type 2 diabetes mellitus with mild nonproliferative diabetic retinopathy without macular edema, bilateral	X

S4 Table. Diabetes diagnosis codes (continued)

System: Code	ICD-10/ICD-10 CM/ICD-10 AM Description	Found
E11.3299	Type 2 diabetes mellitus with mild nonproliferative diabetic retinopathy without macular edema, unspecified eye	X
E11.33	Type 2 diabetes mellitus with moderate nonproliferative diabetic retinopathy	X
E11.331	Type 2 diabetes mellitus with moderate nonproliferative diabetic retinopathy with macular edema	X
E11.3311	Type 2 diabetes mellitus with moderate nonproliferative diabetic retinopathy with macular edema, right eye	X
E11.3312	Type 2 diabetes mellitus with moderate nonproliferative diabetic retinopathy with macular edema, left eye	X
E11.3313	Type 2 diabetes mellitus with moderate nonproliferative diabetic retinopathy with macular edema, bilateral	X
E11.3319	Type 2 diabetes mellitus with moderate nonproliferative diabetic retinopathy with macular edema, unspecified eye	X
E11.339	Type 2 diabetes mellitus with moderate nonproliferative diabetic retinopathy without macular edema	X
E11.3391	Type 2 diabetes mellitus with moderate nonproliferative diabetic retinopathy without macular edema, right eye	X
E11.3392	Type 2 diabetes mellitus with moderate nonproliferative diabetic retinopathy without macular edema, left eye	X
E11.3393	Type 2 diabetes mellitus with moderate nonproliferative diabetic retinopathy without macular edema, bilateral	X
E11.3399	Type 2 diabetes mellitus with moderate nonproliferative diabetic retinopathy without macular edema, unspecified eye	X
E11.34	Type 2 diabetes mellitus with severe nonproliferative diabetic retinopathy	
E11.341	Type 2 diabetes mellitus with severe nonproliferative diabetic retinopathy with macular edema	X
E11.3411	Type 2 diabetes mellitus with severe nonproliferative diabetic retinopathy with macular edema, right eye	X
E11.3412	Type 2 diabetes mellitus with severe nonproliferative diabetic retinopathy with macular edema, left eye	X
E11.3413	Type 2 diabetes mellitus with severe nonproliferative diabetic retinopathy with macular edema, bilateral	X
E11.3419	Type 2 diabetes mellitus with severe nonproliferative diabetic retinopathy with macular edema, unspecified eye	X
E11.349	Type 2 diabetes mellitus with severe nonproliferative diabetic retinopathy without macular edema	
E11.3491	Type 2 diabetes mellitus with severe nonproliferative diabetic retinopathy without macular edema, right eye	X
E11.3492	Type 2 diabetes mellitus with severe nonproliferative diabetic retinopathy without macular edema, left eye	X
E11.3493	Type 2 diabetes mellitus with severe nonproliferative diabetic retinopathy without macular edema, bilateral	X
E11.3499	Type 2 diabetes mellitus with severe nonproliferative diabetic retinopathy without macular edema, unspecified eye	X
E11.35	Type 2 diabetes mellitus with proliferative diabetic retinopathy	
E11.351	Type 2 diabetes mellitus with proliferative diabetic retinopathy with macular edema	X
E11.3511	Type 2 diabetes mellitus with proliferative diabetic retinopathy with macular edema, right eye	X

S4 Table. Diabetes diagnosis codes (continued)

System: Code	ICD-10/ICD-10 CM/ICD-10 AM Description	Found
E11.3512	Type 2 diabetes mellitus with proliferative diabetic retinopathy with macular edema, left eye	X
E11.3513	Type 2 diabetes mellitus with proliferative diabetic retinopathy with macular edema, bilateral	X
E11.3519	Type 2 diabetes mellitus with proliferative diabetic retinopathy with macular edema, unspecified eye	X
E11.352	Type 2 diabetes mellitus with proliferative diabetic retinopathy with traction retinal detachment involving the macula	
E11.3521	Type 2 diabetes mellitus with proliferative diabetic retinopathy with traction retinal detachment involving the macula, right eye	
E11.3522	Type 2 diabetes mellitus with proliferative diabetic retinopathy with traction retinal detachment involving the macula, left eye	
E11.3523	Type 2 diabetes mellitus with proliferative diabetic retinopathy with traction retinal detachment involving the macula, bilateral	X
E11.3529	Type 2 diabetes mellitus with proliferative diabetic retinopathy with traction retinal detachment involving the macula, undefined eye	X
E11.353	Type 2 diabetes mellitus with proliferative diabetic retinopathy with traction retinal detachment not involving the macula	
E11.3531	Type 2 diabetes mellitus with proliferative diabetic retinopathy with traction retinal detachment not involving the macula, right eye	X
E11.3532	Type 2 diabetes mellitus with proliferative diabetic retinopathy with traction retinal detachment not involving the macula, left eye	
E11.3533	Type 2 diabetes mellitus with proliferative diabetic retinopathy with traction retinal detachment not involving the macula, bilateral	
E11.3539	Type 2 diabetes mellitus with proliferative diabetic retinopathy with traction retinal detachment not involving the macula, undefined eye	
E11.354	Type 2 diabetes mellitus with proliferative diabetic retinopathy with combined traction retinal detachment and rhegmatogenous	
E11.3541	Type 2 diabetes mellitus with proliferative diabetic retinopathy with combined traction retinal detachment and rhegmatogenous, right eye	X
E11.3542	Type 2 diabetes mellitus with proliferative diabetic retinopathy with combined traction retinal detachment and rhegmatogenous, left eye	
E11.3543	Type 2 diabetes mellitus with proliferative diabetic retinopathy with combined traction retinal detachment and rhegmatogenous, bilateral	
E11.3549	Type 2 diabetes mellitus with proliferative diabetic retinopathy with combined traction retinal detachment and rhegmatogenus, unspecified eye	
E11.355	Type 2 diabetes mellitus with stable proliferative diabetic retinopathy	
E11.3551	Type 2 diabetes mellitus with stable proliferative diabetic retinopathy, right eye	X
E11.3552	Type 2 diabetes mellitus with stable proliferative diabetic retinopathy, left eye	X
E11.3553	Type 2 diabetes mellitus with stable proliferative diabetic retinopathy, bilateral	X
E11.3559	Type 2 diabetes mellitus with stable proliferative diabetic retinopathy, unspecified eye	X

S4 Table. Diabetes diagnosis codes (continued)

System:	ICD-10/ICD-10 CM/ICD-10 AM	
**Code**	**Description**	**Found**
E11.359	Type 2 diabetes mellitus with proliferative diabetic retinopathy without macular edema	X
E11.3591	Type 2 diabetes mellitus with proliferative diabetic retinopathy without macular edema, right eye	X
E11.3592	Type 2 diabetes mellitus with proliferative diabetic retinopathy without macular edema, left eye	X
E11.3593	Type 2 diabetes mellitus with proliferative diabetic retinopathy without macular edema, bilateral	X
E11.3599	Type 2 diabetes mellitus with proliferative diabetic retinopathy without macular edema, unspecified eye	X
E11.36	Type 2 diabetes mellitus with diabetic cataract	X
E11.37	Type 2 diabetes mellitus with diabetic macular edema, resolved following treatment	
E11.37X1	Type 2 diabetes mellitus with diabetic macular edema, resolved following treatment, right eye	
E11.37X2	Type 2 diabetes mellitus with diabetic macular edema, resolved following treatment, left eye	
E11.37X3	Type 2 diabetes mellitus with diabetic macular edema, resolved following treatment, bilateral	
E11.37X9	Type 2 diabetes mellitus with diabetic macular edema, resolved following treatment, unspecified eye	X
E11.39	Type 2 diabetes mellitus with other diabetic ophthalmic complication	X
E11.4	Type 2 diabetes mellitus, with neurological complications	X
E11.40	Type 2 diabetes mellitus with diabetic neuropathy, unspecified	X
E11.41	Type 2 diabetes mellitus with diabetic mononeuropathy	X
E11.42	Type 2 diabetes mellitus with diabetic polyneuropathy	X
E11.43	Type 2 diabetes mellitus with diabetic autonomic (poly)neuropathy	X
E11.44	Type 2 diabetes mellitus with diabetic amyotrophy	X
E11.49	Type 2 diabetes mellitus with other diabetic neurological complication	X
E11.5	Type 2 diabetes mellitus, with peripheral circulatory complications	X
E11.51	Type 2 diabetes mellitus with diabetic peripheral angiopathy without gangrene	X
E11.52	Type 2 diabetes mellitus with diabetic peripheral angiopathy with gangrene	X
E11.59	Type 2 diabetes mellitus with other circulatory complications	X
E11.6	Type 2 diabetes mellitus, with other specified complications	X
E11.61	Type 2 diabetes mellitus with diabetic arthropathy	
E11.610	Type 2 diabetes mellitus with diabetic neuropathic arthropathy	X
E11.618	Type 2 diabetes mellitus with other diabetic arthropathy	X
E11.62	Type 2 diabetes mellitus with skin complications	
E11.620	Type 2 diabetes mellitus with diabetic dermatitis	X
E11.621	Type 2 diabetes mellitus with foot ulcer	X
E11.622	Type 2 diabetes mellitus with other skin ulcer	X
E11.628	Type 2 diabetes mellitus with other skin complications	X
E11.63	Type 2 diabetes mellitus with oral complications	
E11.630	Type 2 diabetes mellitus with periodontal disease	X
E11.638	Type 2 diabetes mellitus with other oral complications	X
E11.64	Type 2 diabetes mellitus with hypoglycemia	
E11.641	Type 2 diabetes mellitus with hypoglycemia with coma	X
E11.649	Type 2 diabetes mellitus with hypoglycemia without coma	X
E11.65	Type 2 diabetes mellitus with hyperglycemia	X

S4 Table. Diabetes diagnosis codes (continued)

System: Code	ICD-10/ICD-10 CM/ICD-10 AM Description	Found
E11.69	Type 2 diabetes mellitus with other specified complication	X
E11.7	Type 2 diabetes mellitus, with multiple complications	X
E11.8	Type 2 diabetes mellitus, with unspecified complications	X
E11.9	Type 2 diabetes mellitus, without complications	X
E12	Malnutrition-related diabetes mellitus	
E12.0	Malnutrition-related diabetes mellitus with coma	X
E12.1	Malnutrition-related diabetes mellitus with ketoacidosis	X
E12.2	Malnutrition-related diabetes mellitus with renal complications	X
E12.3	Malnutrition-related diabetes mellitus with ophthalmic complications	X
E12.4	Malnutrition-related diabetes mellitus with neurological complications	X
E12.5	Malnutrition-related diabetes mellitus with peripheral circulatory complications	X
E12.6	Malnutrition-related diabetes mellitus with other specified complications	X
E12.7	Malnutrition-related diabetes mellitus with multiple complications	X
E12.8	Malnutrition-related diabetes mellitus with unspecified complications	X
E12.9	Malnutrition-related diabetes mellitus without complications	X
E13	Other specified diabetes mellitus	X
E13.0	Other specified diabetes mellitus with hyperosmolarity	X
E13.0	Other specified diabetes mellitus with coma	
E13.00	Other specified diabetes mellitus with hyperosmolarity without nonketotic hyperglycemic-hyperosmolar coma (nkhhc)	X
E13.01	Other specified diabetes mellitus with hyperosmolarity with coma	
E13.1	Other specified diabetes mellitus with ketoacidosis	X
E13.10	Other specified diabetes mellitus with ketoacidosis without coma	X
E13.11	Other specified diabetes mellitus with ketoacidosis with coma	
E13.2	Other specified diabetes mellitus with kidney complications	X
E13.21	Other specified diabetes mellitus with diabetic nephropathy	X
E13.22	Other specified diabetes mellitus with diabetic chronic kidney disease	X
E13.29	Other specified diabetes mellitus with other diabetic kidney complication	X
E13.3	Other specified diabetes mellitus with ophthalmic complications	X
E13.31	Other specified diabetes mellitus with unspecified diabetic retinopathy	
E13.311	Other specified diabetes mellitus with unspecified diabetic retinopathy with macular edema	X
E13.319	Other specified diabetes mellitus with unspecified diabetic retinopathy without macular edema	X
E13.32	Other specified diabetes mellitus with mild nonproliferative diabetic retinopathy	
E13.321	Other specified diabetes mellitus with mild nonproliferative diabetic retinopathy with macular edema	
E13.3211	Other specified diabetes mellitus with mild nonproliferative diabetic retinopathy with macular edema, right eye	
E13.3212	Other specified diabetes mellitus with mild nonproliferative diabetic retinopathy with macular edema, left eye	
E13.3213	Other specified diabetes mellitus with mild nonproliferative diabetic retinopathy with macular edema, bilateral	X
E13.3219	Other specified diabetes mellitus with mild nonproliferative diabetic retinopathy with macular edema, unspecified eye	
E13.329	Other specified diabetes mellitus with mild nonproliferative diabetic retinopathy without macular edema	

S4 Table. Diabetes diagnosis codes (continued)

System: Code	ICD-10/ICD-10 CM/ICD-10 AM Description	Found
E13.3291	Other specified diabetes mellitus with mild nonproliferative diabetic retinopathy without macular edema, right eye	X
E13.3292	Other specified diabetes mellitus with mild nonproliferative diabetic retinopathy without macular edema, left eye	X
E13.3293	Other specified diabetes mellitus with mild nonproliferative diabetic retinopathy without macular edema, bilateral	X
E13.3299	Other specified diabetes mellitus with mild nonproliferative diabetic retinopathy without macular edema, unspecified eye	X
E13.33	Other specified diabetes mellitus with moderate nonproliferative diabetic retinopathy	
E13.331	Other specified diabetes mellitus with moderate nonproliferative diabetic retinopathy with macular edema	
E13.3311	Other specified diabetes mellitus with moderate nonproliferative diabetic retinopathy with macular edema, right eye	X
E13.3312	Other specified diabetes mellitus with moderate nonproliferative diabetic retinopathy with macular edema, left eye	
E13.3313	Other specified diabetes mellitus with moderate nonproliferative diabetic retinopathy with macular edema, bilateral	
E13.3319	Other specified diabetes mellitus with moderate nonproliferative diabetic retinopathy with macular edema, unspecified eye	
E13.339	Other specified diabetes mellitus with moderate nonproliferative diabetic retinopathy without macular edema	
E13.3391	Other specified diabetes mellitus with moderate nonproliferative diabetic retinopathy without macular edema, right eye	X
E13.3392	Other specified diabetes mellitus with moderate nonproliferative diabetic retinopathy without macular edema, left eye	
E13.3393	Other specified diabetes mellitus with moderate nonproliferative diabetic retinopathy without macular edema, bilateral	X
E13.3399	Other specified diabetes mellitus with moderate nonproliferative diabetic retinopathy without macular edema, unspecified eye	
E13.34	Other specified diabetes mellitus with severe nonproliferative diabetic retinopathy	
E13.341	Other specified diabetes mellitus with severe nonproliferative diabetic retinopathy with macular edema	
E13.3411	Other specified diabetes mellitus with severe nonproliferative diabetic retinopathy with macular edema, right eye	
E13.3412	Other specified diabetes mellitus with severe nonproliferative diabetic retinopathy with macular edema, left eye	
E13.3413	Other specified diabetes mellitus with severe nonproliferative diabetic retinopathy with macular edema, bilateral	
E13.3419	Other specified diabetes mellitus with severe nonproliferative diabetic retinopathy with macular edema, unspecified eye	
E13.349	Other specified diabetes mellitus with severe nonproliferative diabetic retinopathy without macular edema	
E13.3491	Other specified diabetes mellitus with severe nonproliferative diabetic retinopathy without macular edema, right eye	
E13.3492	Other specified diabetes mellitus with severe nonproliferative diabetic retinopathy without macular edema, left eye	
E13.3493	Other specified diabetes mellitus with severe nonproliferative diabetic retinopathy without macular edema, bilateral	

S4 Table. Diabetes diagnosis codes (continued)

System:<br>Code	ICD-10/ICD-10 CM/ICD-10 AM<br>Description	Found
E13.3499	Other specified diabetes mellitus with severe nonproliferative diabetic retinopathy without macular edema, unspecified eye	
E13.35	Other specified diabetes mellitus with proliferative diabetic retinopathy	
E13.351	Other specified diabetes mellitus with proliferative diabetic retinopathy with macular edema	
E13.3511	Other specified diabetes mellitus with proliferative diabetic retinopathy with macular edema, right eye	
E13.3512	Other specified diabetes mellitus with proliferative diabetic retinopathy with macular edema, left eye	
E13.3513	Other specified diabetes mellitus with proliferative diabetic retinopathy with macular edema, bilateral	
E13.3519	Other specified diabetes mellitus with proliferative diabetic retinopathy with macular edema, unspecified eye	X
E13.352	Other specified diabetes mellitus with proliferative diabetic retinopathy with traction retinal detachment involving the macula	
E13.3521	Other specified diabetes mellitus with proliferative diabetic retinopathy with traction retinal detachment involving the macula, right eye	
E13.3522	Other specified diabetes mellitus with proliferative diabetic retinopathy with traction retinal detachment involving the macula, left eye	
E13.3522	Other specified diabetes mellitus with proliferative diabetic retinopathy with traction retinal detachment involving the macula, left eye	
E13.3523	Other specified diabetes mellitus with proliferative diabetic retinopathy with traction retinal detachment involving the macula, bilateral	
E13.3529	Other specified diabetes mellitus with proliferative diabetic retinopathy with traction retinal detachment involving the macula, unspecified eye	
E13.353	Other specified diabetes mellitus with proliferative diabetic retinopathy with traction retinal detachment not involving the macula	
E13.3531	Other specified diabetes mellitus with proliferative diabetic retinopathy with traction retinal detachment not involving the macula, right eye	
E13.3532	Other specified diabetes mellitus with proliferative diabetic retinopathy with traction retinal detachment not involving the macula, left eye	
E13.3533	Other specified diabetes mellitus with proliferative diabetic retinopathy with traction retinal detachment not involving the macula, bilateral	
E13.3539	Other specified diabetes mellitus with proliferative diabetic retinopathy with traction retinal detachment not involving the macula, unspecified eye	
E13.354	Other specified diabetes mellitus with proliferative diabetic retinopathy with combined traction retinal detachment and rhegmatogenous retinal detachment	
E13.3541	Other specified diabetes mellitus with proliferative diabetic retinopathy with combined traction retinal detachment and rhegmatogenous retinal detachment, right eye	
E13.3542	Other specified diabetes mellitus with proliferative diabetic retinopathy with combined traction retinal detachment and rhegmatogenous retinal detachment, left eye	X

S4 Table. Diabetes diagnosis codes (continued)

System:	ICD-10/ICD-10 CM/ICD-10 AM	
**Code**	**Description**	**Found**
E13.3543	Other specified diabetes mellitus with proliferative diabetic retinopathy with combined traction retinal detachment and rhegmatogenous retinal detachment, bilateral	
E13.3549	Other specified diabetes mellitus with proliferative diabetic retinopathy with combined traction retinal detachment and rhegmatogenous retinal detachment, unspecified eye	
E13.355	Other specified diabetes mellitus with stable proliferative diabetic retinopathy	
E13.3551	Other specified diabetes mellitus with stable proliferative diabetic retinopathy, right eye	
E13.3552	Other specified diabetes mellitus with stable proliferative diabetic retinopathy, left eye	
E13.3553	Other specified diabetes mellitus with stable proliferative diabetic retinopathy, bilateral	
E13.3559	Other specified diabetes mellitus with stable proliferative diabetic retinopathy, unspecified eye	
E13.359	Other specified diabetes mellitus with proliferative diabetic retinopathy without macular edema	
E13.3591	Other specified diabetes mellitus with proliferative diabetic retinopathy without macular edema, right eye	
E13.3592	Other specified diabetes mellitus with proliferative diabetic retinopathy without macular edema, left eye	X
E13.3593	Other specified diabetes mellitus with proliferative diabetic retinopathy without macular edema, bilateral	X
E13.3599	Other specified diabetes mellitus with proliferative diabetic retinopathy without macular edema, unspecified eye	X
E13.36	Other specified diabetes mellitus with diabetic cataract	X
E13.37	Other specified diabetes mellitus with diabetic macular edema, resolved following treatment	
E13.39	Other specified diabetes mellitus with other diabetic ophthalmic complication	X
E13.4	Other specified diabetes mellitus with neurological complications	X
E13.40	Other specified diabetes mellitus with diabetic neuropathy, unspecified	X
E13.41	Other specified diabetes mellitus with diabetic mononeuropathy	
E13.42	Other specified diabetes mellitus with diabetic polyneuropathy	X
E13.43	Other specified diabetes mellitus with diabetic autonomic (poly)neuropathy	X
E13.44	Other specified diabetes mellitus with diabetic amyotrophy	X
E13.49	Other specified diabetes mellitus with other diabetic neurological complication	X
E13.5	Other specified diabetes mellitus with circulatory complications	X
E13.51	Other specified diabetes mellitus with diabetic peripheral angiopathy without gangrene	X
E13.52	Other specified diabetes mellitus with diabetic peripheral angiopathy with gangrene	
E13.59	Other specified diabetes mellitus with other circulatory complications	X
E13.6	Other specified diabetes mellitus with other specified complications	X
E13.61	Other specified diabetes mellitus with diabetic arthropathy	
E13.610	Other specified diabetes mellitus with diabetic neuropathic arthropathy	X
E13.618	Other specified diabetes mellitus with other diabetic arthropathy	

S4 Table. Diabetes diagnosis codes (continued)

System:	ICD-10/ICD-10 CM/ICD-10 AM	
**Code**	**Description**	**Found**
E13.62	Other specified diabetes mellitus with skin complications	
E13.620	Other specified diabetes mellitus with diabetic dermatitis	
E13.621	Other specified diabetes mellitus with foot ulcer	X
E13.622	Other specified diabetes mellitus with other skin ulcer	
E13.628	Other specified diabetes mellitus with other skin complications	
E13.63	Other specified diabetes mellitus with oral complications	
E13.630	Other specified diabetes mellitus with periodontal disease	
E13.638	Other specified diabetes mellitus with other oral complications	X
E13.64	Other specified diabetes mellitus with hypoglycemia	
E13.641	Other specified diabetes mellitus with hypoglycemia with coma	
E13.649	Other specified diabetes mellitus with hypoglycemia without coma	X
E13.65	Other specified diabetes mellitus with hyperglycemia	X
E13.69	Other specified diabetes mellitus with other specified complication	X
E13.7	Other specified diabetes mellitus with multiple complications	X
E13.8	Other specified diabetes mellitus with unspecified complications	X
E13.9	Other specified diabetes mellitus without complications	X
E13.620	Other specified diabetes mellitus with diabetic dermatitis	
E13.621	Other specified diabetes mellitus with foot ulcer	X
E13.622	Other specified diabetes mellitus with other skin ulcer	
E13.628	Other specified diabetes mellitus with other skin complications	
E14	Unspecified diabetes mellitus	
E14.0	Unspecified diabetes mellitus with coma	X
E14.1	Unspecified diabetes mellitus with ketoacidosis	X
E14.2	Unspecified diabetes mellitus with renal complications	X
E14.3	Unspecified diabetes mellitus with ophthalmic complications	X
E14.4	Unspecified diabetes mellitus with neurological complications	X
E14.5	Unspecified diabetes mellitus with peripheral circulatory complications	X
E14.6	Unspecified diabetes mellitus with other specified complications	X
E14.7	Unspecified diabetes mellitus with multiple complications	X
E14.8	Unspecified diabetes mellitus with unspecified complications	X
E14.9	Unspecified diabetes mellitus without complications	X

System:	SNOMED CT	
**Code**	**Description**	**Found**
4855003	Diabetic retinopathy	
8801005	Secondary diabetes mellitus	
11530004	Unstable diabetes mellitus	X
34140002	Gastroparesis, diabetic	
38205001	Diarrhea due to diabetes mellitus	
44054006	Type 2 diabetes mellitus	X
46635009	Type 1 diabetes mellitus	X
73211009	Diabetes mellitus	X
82980005	Anemia due to diabetes mellitus	
127012008	Lipoatrophic diabetes	
127013003	Nephropathy; diabetic	
127014009	Peripheral vascular disease, diabetic	
190342007	Diabetes mellitus with nephropathy nos	X
190447002	Steroid-induced diabetes	
237599002	Insulin treated type 2 diabetes mellitus	X

S4 Table. Diabetes diagnosis codes (continued)

System:	SNOMED CT	
**Code**	**Description**	**Found**
270445003	Diabetes monitoring check done	
280137006	Left diabetic foot	
290002008	Unstable type 1 diabetes mellitus	
308505000	Diabetes monitoring call	
371087003	Foot ulcer, diabetic	
390833005	Steroid-induced diabetes	
394725008	Diabetes medication review	
405096004	Diabetes self-management behavior	
405749004	Newly diagnosed diabetes	
417317008	Care plan, diabetic	
426875007	Latent autoimmune diabetes mellitus in adult	X
445353002	Unstable type 2 diabetes mellitus	
609561005	Maturity-onset diabetes of the young	X
735985000	Diabetes self management plan	
878859007	Self management of diabetes	
530558861000132104	Atypical diabetes mellitus	
190447002	Steroid-induced diabetes	
237599002	Insulin treated type 2 diabetes mellitus	X
270445003	Diabetes monitoring check done	
280137006	Left diabetic foot	
290002008	Unstable type 1 diabetes mellitus	
308505000	Diabetes monitoring call	
371087003	Foot ulcer, diabetic	
390833005	Steroid-induced diabetes	
394725008	Diabetes medication review	
405096004	Diabetes self-management behavior	
405749004	Newly diagnosed diabetes	
417317008	Care plan, diabetic	
426875007	Latent autoimmune diabetes mellitus in adult	X
445353002	Unstable type 2 diabetes mellitus	
609561005	Maturity-onset diabetes of the young	X
735985000	Diabetes self management plan	
878859007	Self management of diabetes	
530558861000132104	Atypical diabetes mellitus	

System:	ICPC-2	
**Code**	**Description**	**Found**
T89	Diabetes insulin dependent	X
T90	Diabetes non-insulin dependent	X

System:	OHIP	
**Code**	**Description**	**Found**
248	Diabetes mellitus with ocular complications	X
250	Diabetes mellitus (including complications)	X
