# Supplementary material for: Changes in reasons for visits to primary care after the start of the COVID-19 pandemic: An international comparative study by the International Consortium of Primary Care Big Data Researchers (INTRePID)
Source: PLOS Glob Public Health. 2024 Aug 22;4(8):e0003406. doi: 10.1371/journal.pgph.0003406 (PMC11341054; doi:10.1371/journal.pgph.0003406)
Supplement: S5 Table — (PDF) [file pgph.0003406.s005.pdf]

S5 Table. Hypercholesterolemia diagnosis codes

System:	ICD-10/ICD-10 CM/ICD-10 AM	
**Code**	**Description**	**Found**
E78	Disorders of lipoprotein metabolism and other lipidemias	X
E78.0	Pure hypercholesterolaemia	X
E78.00	Pure hypercholesterolemia, unspecified	X
E78.01	Familial hypercholesterolemia	X
E78.1	Pure hyperglyceridemia	X
E78.2	Mixed hyperlipidemia	X
E78.3	Hyperchylomicronaemia	X
E78.4	Other hyperlipidemia	X
E78.41	Elevated Lipoprotein(a)	X
E78.49	Other hyperlipidemia	X
E78.5	Hyperlipidemia, unspecified	X
E78.6	Lipoprotein deficiency	X
E78.7	Disorders of bile acid and cholesterol metabolism	
E78.70	Disorder of bile acid and cholesterol metabolism, unspecified	X
E78.79	Other disorders of bile acid and cholesterol metabolism	X
E78.8	Other disorders of lipoprotein metabolism	X
E78.89	Other lipoprotein metabolism disorders	X
E78.9	Disorder of lipoprotein metabolism, unspecified	X

System:	SNOMED CT	
**Code**	**Description**	**Found**
13644009	Hypercholesterolaemia	
55822004	Severe dyslipidemia	X
77063006	Hypertriglyceridemia	
129591001	Mixed hypercholesterolemia and hypertriglyceridemia	
154740003	(Hypercholesterolaemia[pure] or [familial]) or (xanthoma - congenital)	
166818002	Lipids abnormal	
190777009	Other specified pure hypercholesterolaemia	
190778004	Pure hypercholesterolaemia NOS	
238076009	Primary hypercholesterolaemia	
238077000	Polygenic hypercholesterolemia	
238082007	Secondary hypercholesterolemia	
267432004	Pure hypercholesterolemia	
267500001	(Hypercholesterolaemia[pure] or [familial]) or (xanthoma - congenital)	
302870006	Hypertriglyceridemia	X
370992007	Dyslipidemia	X
398036000	Familial hypercholesterolemia	X
444059002	Hypercholesterolemia well controlled	
773726000	Hypercholesterolemia due to cholesterol 7alpha-hydroxylase deficiency	
67991000119104	Serum cholesterol abnormal	

System:	ICPC-2	
**Code**	**Description**	**Found**
T93	Lipid disorder	X

System:	OHIP	
**Code**	**Description**	**Found**
272	Hypercholesterolemia	X
