# Supplementary material for: Changes in reasons for visits to primary care after the start of the COVID-19 pandemic: An international comparative study by the International Consortium of Primary Care Big Data Researchers (INTRePID)
Source: PLOS Glob Public Health. 2024 Aug 22;4(8):e0003406. doi: 10.1371/journal.pgph.0003406 (PMC11341054; doi:10.1371/journal.pgph.0003406)
Supplement: S6 Table — (PDF) [file pgph.0003406.s006.pdf]

S6 Table. Hypertension diagnosis codes

System:	ICD-10/ICD-10 CM/ICD-10 AM	
**Code**	**Description**	**Found**
I10	Essential(primary) hypertension	X
I10.1	Malignant hypertension	X
I10.9	Other and unspecified primary hypertension	X
I11	Hypertensive heart disease	
I11.0	Hypertensive heart disease with (congestive) heart failure	X
I11.9	Hypertensive heart disease without (congestive) heart failure	X
I12	Hypertensive renal disease	
I12.0	Hypertensive renal disease with renal failure	X
I12.9	Hypertensive renal disease without renal failure	X
I13	Hypertensive heart and renal disease	
I13.0	Hypertensive heart and renal disease with (congestive) heart failure	X
I13.1	Hypertensive heart and renal disease with renal failure	X
I13.10	Hypertensive heart and chronic kidney disease without heart failure, with stage 1 through stage 4 chronic kidney disease	X
I13.11	Hypertensive heart and chronic kidney disease without heart failure, with stage 5 chronic kidney disease	X
I13.2	Hypertensive heart and renal disease with both (congestive) heart failure and renal failure	X
I13.9	Hypertensive heart and renal disease, unsp	X
I15	Secondary hypertension	X
I15.0	Renovascular hypertension	X
I15.1	Hypertension secondary to other renal disorders	X
I15.2	Hypertension secondary to endocrine disorders	X
I15.8	Other secondary hypertension	X
I15.9	Secondary hypertension, unspecified	X
I16.0	Hypertensive urgency	X
I16.1	Hypertensive emergency	X
I16.9	Hypertensive crisis, unspecified	X
I13.11	Hypertensive heart and chronic kidney disease without heart failure, with stage 5 chronic kidney disease	X

System:	SNOMED CT	
**Code**	**Description**	**Found**
1201005	Benign essential hypertension	
1218009	Accelerated essential hypertension	
10725009	Benign hypertension	
19769006	High-renin essential hypertension	
23130000	Paroxysmal hypertension	
28119000	Renal hypertension	
31992008	Secondary hypertension	
38341003	Hypertension	X
46481004	Low-renin essential hypertension	
48146000	Diastolic hypertension	
56218007	Systolic hypertension	
59621000	Essential hypertension	
59720008	Sustained diastolic hypertension	
65518004	Labile diastolic hypertension	
71874008	Benign essential hypertension complicating and/or reason for care during childbirth	

S6 Table. Hypertension diagnosis codes (continued)

System:	SNOMED CT	
**Code**	**Description**	**Found**
74451002	Secondary diastolic hypertension	
78975002	Malignant essential hypertension	
84094009	Rebound hypertension	
89242004	Malignant secondary hypertension	
123799005	Hypertension, renovascular	
155296003	Essential hypertension	
194758001	Benign essential hypertension	
194760004	Hypertension NOS (& [essential])	
194785008	Benign secondary hypertension	
194788005	Hypertension secondary to endocrine disorder	
194791005	Hypertension secondary to drug	
195225002	Hypertensive: [encephalopathy] or [crisis]	
266228004	Essential hypertension NOS	
371125006	Labile essential hypertension	
397748008	Hypertension with albuminuria	
427889009	Hypertension associated with transplantation	
429198000	Exertional hypertension	
429457004	Systolic essential hypertension	
697929007	Intermittent hypertension	
704667004	Hypertension concurrent and due to end stage renal disease on dialysis	
706882009	Hypertensive crisis	
712832005	Supine hypertension	
762463000	Diastolic hypertension and systolic hypertension	
766937004	Hypertension due to gain-of-function mutation in mineralocorticoid receptor	
827068008	Hypertension stage 2	
827069000	Hypertension stage 1	
871642009	Hypertension due to aortic arch obstruction	
71421000119105	Hypertension in chronic kidney disease due to type 2 diabetes mellitus	
71701000119105	Hypertension in chronic kidney disease due to type 1 diabetes mellitus	
461301000124109	Resistant hypertensive disorder	
1078301000112109	Multiple drug intolerant hypertension	

System:	ICPC-2	
**Code**	**Description**	**Found**
K86	Hypertension uncomplicated	X
K87	Hypertension complicated	X

System:	OHIP	
**Code**	**Description**	**Found**
401	Hypertension, essential	X
402	Hypertensive heart disease	X
403	Hypertensive renal disease	X
437	Encephalopathy, hypertensive	X
