# Supplementary material for: Changes in reasons for visits to primary care after the start of the COVID-19 pandemic: An international comparative study by the International Consortium of Primary Care Big Data Researchers (INTRePID)
Source: PLOS Glob Public Health. 2024 Aug 22;4(8):e0003406. doi: 10.1371/journal.pgph.0003406 (PMC11341054; doi:10.1371/journal.pgph.0003406)
Supplement: S7 Table — (PDF) [file pgph.0003406.s007.pdf]

S7 Table. Top 10 reasons for total visits to primary care in 2019

Country	Top 10 reasons for visit 2019	Mean monthly visits (SD)
**Argentina**	1. General Health Exam	17,692 (3,581)
	2. Contraception	6,027 (578)
	3. Gynaecologic Exam	5,234 (711)
	4. Routine Child Health Exam	4,381 (811)
	5. Pregnancy	3,232 (266)
	6. Overweight/Obesity	3,076 (328)
	7. Economic/Housing Problems	2,783 (610)
	8. Bronchiolitis	2,547 (1,299)
	9. Diabetes	2,334 (359)
	10. URTI	2,267 (1,006)
**Australia**	1. URTI	4,699 (1,807)
	2. Hypertension	2,715 (263)
	3. Diabetes	1,772 (141)
	4. Cough	1,300 (349)
	5. Back Pain	1,230 (86)
	6. General Health Exam	1,213 (90)
	7. Anxiety	968 (114)
	8. Asthma	951 (167)
	9. Depression	898 (67)
	10. Urinary Tract Infection	860 (48)
**Canada**	1. Anxiety	4,417 (314)
	2. Diabetes	4,326 (413)
	3. Hypertension	4,130 (437)
	4. Common Cold	2,494 (714)
	5. Musculosketal Pain	2,117 (206)
	6. Ill Defined Condition	2,098 (177)
	7. Abdominal Pain	2,053 (182)
	8. Well Baby Visit	2,047 (174)
	9. Pregnancy	1,894 (136)
	10. General Health Exam	1,726 (181)
**China**	1. Pregnancy	834 (134)
	2. URTI	511 (202)
	3. Post-partum Complaint	426 (138)
	4. Anxiety	243 (27)
	5. Sleep Disturbance	238 (25)
	6. General Health Exam	228 (249)
	7. Cough	198 (51)
	8. Hypertension	182 (32)
	9. Depression	153 (38)
	10. Hyperlipidemia	148 (24)
**Norway**	1. Hypertension	40,806 (6,776)
	2. URTI	38,821 (10,270)
	3. Depression	29,547 (4,455)
	4. Diabetes	28,400 (4,617)
	5. General Health Exam	21,435 (6,254)
	6. Fatigue	17,973 (2,765)
	7. Abdominal Pain	17,485 (2,223)
	8. Atrial Fibrillation/Flutter	17,309 (1,270)
	9. Pregnancy	16,291 (1,310)
	10. Urinary Tract Infection	16,093 (1,745)

S7 Table. Top 10 reasons for total visits to primary care in 2019 (continued)

Country	Top 10 reasons for visit 2019	Mean monthly visits (SD)
**Peru**	1. Contraception	476,035 (152,152)
	2. General Health Exam	424,311 (28,061)
	3. Iron Deficiency Anemia	229,676 (31,054)
	4. Pharyngitis	193,270 (25,380)
	5. Dental Caries	180,380 (21,083)
	6. Pregnancy	178,530 (8,225)
	7. Common Cold	128,760 (17,209)
	8. Obesity	115,029 (9,466)
	9. Cystitis	108,863 (7,192)
	10. Fever	81,874 (14,295)
**Singapore**	1. URTI	18,315 (2,010)
	2. Diabetes	14,052 (1,156)
	3. Hypertension	10,764 (1,153)
	4. General Symptoms/Signs	4,339 (416)
	5. Hyperlipidemia	4,199 (419)
	6. Gastroenteritis	3,835 (462)
	7. Skin Disorder	3,428 (234)
	8. General Health Exam	2,781 (232)
	9. Headache	2,243 (315)
	10. Follow-up Visit	1,936 (194)
**Sweden**	1. Hypertension	2,255 (522)
	2. Diabetes	1,835 (484)
	3. Acute Stress Reaction	1,192 (216)
	4. Anxiety	1,131 (190)
	5. Soft Tissue Disorder	1,047 (110)
	6. URTI	910 (284)
	7. Back Pain	902 (111)
	8. Depression	810 (127)
	9. Abdominal/Pelvic Pain	712 (112)
	10. General Health Exam	671 (86)
**United States**	1. General Health Exam	3,175 (320)
	2. Hypertension	2,551 (190)
	3. Hyperlipidemia	2,544 (173)
	4. Diabetes	1,704 (143)
	5. Overweight/Obesity	969 (88)
	6. Back Pain	921 (66)
	7. Joint Disorder	901 (93)
	8. Elevated Blood Sugar	831 (70)
	9. Depression	804 (72)
	10. Vit D Deficiency	756 (42)
