# Supplementary material for: Changes in reasons for visits to primary care after the start of the COVID-19 pandemic: An international comparative study by the International Consortium of Primary Care Big Data Researchers (INTRePID)
Source: PLOS Glob Public Health. 2024 Aug 22;4(8):e0003406. doi: 10.1371/journal.pgph.0003406 (PMC11341054; doi:10.1371/journal.pgph.0003406)
Supplement: S8 Table — (PDF) [file pgph.0003406.s008.pdf]

S8 Table. Top 10 reasons for in-person visits to primary care in 2020

Country	Top 10 reasons for in-person visits 2020	Mean monthly visits (SD)
**Argentina**	1. General Health Exam	8,139 (4,786)
	2. Contraception	6,211 (2,327)
	3. Pregnancy	3,065 (958)
	4. Diabetes	2,852 (1,078)
	5. COVID-19	3,049 (3,044)
	6. Economic Problems	2,467 (842)
	7. Hypertension	2,088 (703)
	8. Gynaecologic Exam	2,049 (1,593)
	9. Child Health Exam	1,724 (1,045)
	10. Anxiety	1,389 (589)
**Australia**	1. Hypertension	2,334 (643)
	2. URTI	1,561 (1,859)
	3. Diabetes	1,354 (373)
	4. General Health Exam	1,088 (307)
	5. Back Pain	997 (270)
	6. Osteoporosis	812 (243)
	7. Anxiety	775 (235)
	8. Gastroesophageal Reflux	764 (202)
	9. Asthma	751 (326)
	10. Urinary Tract Infection	627 (198)
**Canada**	1. Diabetes	1,891 (1,429)
	2. Well Baby Visit	1,690 (585)
	3. Hypertension	1,672 (1,439)
	4. Anxiety	1,587 (1,581)
	5. Pregnancy	1,431 (451)
	6. Ill Defined Condition	921 (691)
	7. Abdominal Pain	904 (692)
	8. Common Cold	865 (1,386)
	9. Musculoskeletal Pain	831 (668)
	10. Eczema	579 (395)
**China**	1. General Health Exam	774 (545)
	2. Pregnancy	559 (171)
	3. Post-partum Complaint	452 (198)
	4. Hypertension	316 (297)
	5. Anxiety	273 (91)
	6. Sleep Disturbance	231 (84)
	7. Diabetes	198 (198)
	8. Hyperlipidemia	180 (118)
	9. URTI	151 (104)
	10. Other Infections	149 (110)
**Norway**	1. Hypertension	40,813 (14,464)
	2. Diabetes	29,636 (10,201)
	3. Depression	25,715 (9,818)
	4. General Health Exam	23,512 (17,685)
	5. URTI	17,258 (18,659)
	6. Pregnancy	15,796 (4,915)
	7. Abdominal Pain	15,743 (5,642)
	8. Atrial Fibrillation/Flutter	15,306 (4,686)
	9. Urinary Tract Infection	14,838 (5,195)
	10. Fatigue	14,579 (6,061)

S8 Table. Top 10 reasons for in person visits to primary care in 2020 (continued)

Country	Top 10 reasons for in-person visits 2020	Mean monthly visits (SD)
**Peru**	1. Contraception	336,242 (118,537)
	2. General Health Exam	209,443 (133,551)
	3. Pregnancy	144,781 (61,490)
	4. Pharyngitis	86,003 (43,488)
	5. Urinary Tract Infection	65,843 (36,418)
	6. Common Cold	59,264 (32,352)
	7. Dental Caries	57,081 (56,441)
	8. Obesity/Overweight	55,383 (41,327)
	9. Fever	52,989 (23,007)
	10. Puerperium	49,554 (18,125)
**Singapore**	1. Diabetes	14,270 (4,889)
	2. Hypertension	10,526 (3,540)
	3. URTI	9,684 (6,944)
	4. Hyperlipidemia	4,657 (1,467)
	5. General Symptoms/Signs	3,004 (1,896)
	6. General Health Exam	2,714 (1,058)
	7. Gastroenteritis	1,880 (1,261)
	8. Headache	1,481 (737)
	9. Arthrosis	1,113 (741)
	10. Soft Tissue Disorder	777 (355)
**Sweden**	1. Hypertension	2,122 (816)
	2. Diabetes	1,729 (772)
	3. Anxiety	1,099 (400)
	4. Acute Stress Reaction	994 (361)
	5. Soft Tissue Disorder	913 (318)
	6. Back Pain	776 (265)
	7. Abdominal/Pelvic Pain	709 (227)
	8. Depression	690 (234)
	9. General Health Exam	644 (235)
	10. Pain	630 (210)
**United States**	1. General Heath Exam	2,840 (1,486)
	2. Hyperlipidemia	1,914 (962)
	3. Hypertension	1,868 (878)
	4. Diabetes	1,281 (596)
	5. Back Pain	656 (303)
	6. Joint disorder	654 (295)
	7. Obesity/Overweight	652 (312)
	8. Vit D Deficiency	629 (319)
	9. Elevated Blood Sugar	607 (301)
	10. Depression	571 (310)
