# Supplementary material for: Changes in reasons for visits to primary care after the start of the COVID-19 pandemic: An international comparative study by the International Consortium of Primary Care Big Data Researchers (INTRePID)
Source: PLOS Glob Public Health. 2024 Aug 22;4(8):e0003406. doi: 10.1371/journal.pgph.0003406 (PMC11341054; doi:10.1371/journal.pgph.0003406)
Supplement: S9 Table — (PDF) [file pgph.0003406.s009.pdf]

S9 Table. Top 10 reasons for virtual visits to primary care in 2020

Country	Top 10 reasons for virtual visits 2020	Mean monthly visits (SD)
**Australia**	1. Hypertension	831 (670)
	2. URTI	805 (591)
	3. Diabetes	745 (600)
	4. Active Listening	683 (592)
	5. Anxiety	636 (518)
	6. Back Pain	557 (457)
	7. Gastroesophageal Reflux	494 (433)
	8. Medication Review	448 (353)
	9. Mixed Anxiety/Depression	448 (368)
	10. Depression	433 (335)
**Canada**	1. Anxiety	4,747 (2,869)
	2. Hypertension	2,131 (1,246)
	3. Diabetes	2,115 (1,273)
	4. Ill Defined Condition	1,830 (1,202)
	5. Abdominal Pain	1,705 (1,127)
	6. Musculosketal Pain	1,538 (1,021)
	7. Back Pain	929 (617)
	8. Common Cold	905 (729)
	9. Atopic Dermatitis	895 (559)
	10. Chest Pain/Palpitations	841 (530)
**Norway**	1. COVID-19 Suspected	18,921 (15,465)
	2. Fear of Respiratory Disease	17,073 (15,102)
	3. URTI	10,799 (8,308)
	4. Depression	8,412 (4,645)
	5. Acute Stress Reaction	4,896 (2,662)
	6. COVID-19 confirmed	4,125 (6,714)
	7. Fatigue	3,638 (1,995)
	8. Cough	3,601 (2,233)
	9. Fear of Other Diseases	3,577 (3,812)
	10. Psychological Complaint	3,520 (1,933)
**Peru**	1. Anxiety	5,692 (5,131)
	2. Depression	2,995 (2,734)
	3. Acute Stress Reaction	2,177 (1,982)
	4. Anemia	1,248 (1,148)
	5. Emotional Distress	1,146 (1,174)
	6. Chest/Throat Pain	1,018 (905)
	7. Common Cold	985 (933)
	8. Physical/Psycological Abuse	975 (1,009)
	9. Overweight/Obesity	891 (910)
	10. Cough	867 (786)
**Sweden**	1. Anxiety	233 (125)
	2. Diabetes	202 (84)
	3. Acute Stress Reaction	195 (103)
	4. URTI	173 (189)
	5. Hypertension	133 (48)
	6. Depression	127 (60)
	7. Urinary Tract Infection	105 (42)
	8. COVID-19	103 (118)
	9. Counselling	102 (46)
	10. Pain	92 (36)

S9 Table. Top 10 reasons for virtual visits to primary care in 2020 (continued)

Country	Top 10 reasons for virtual visits 2020	Mean monthly visits (SD)
**United States**	1. Hyperlipidemia	617 (419)
	2. Hypertension	602 (421)
	3. Diabetes	432 (306)
	4. General Health Exam	409 (294)
	5. Anxiety	295 (190)
	6. Depression	252 (167)
	7. Hypothyroidism	218 (141)
	8. Back Pain	217 (137)
	9. Vit D Deficiency	185 (123)
	10. Elevated Blood Sugar	182 (125)
