# Supplementary material for: Changes in reasons for visits to primary care after the start of the COVID-19 pandemic: An international comparative study by the International Consortium of Primary Care Big Data Researchers (INTRePID)
Source: PLOS Glob Public Health. 2024 Aug 22;4(8):e0003406. doi: 10.1371/journal.pgph.0003406 (PMC11341054; doi:10.1371/journal.pgph.0003406)
Supplement: S1 Data — (PDF) [file pgph.0003406.s010.pdf]

S1 Data. Data sources

Argentina

Argentina has nominal universal health care [1] comprised of three systems: public healthcare, private coverage, and health insurance (formal workers). According to the 2022 report of the General Direction of Statistics and Censuses [2] in the city of Buenos Aires, 20% of the population is covered by public healthcare, while 80% is covered by the other two systems. However, in the most vulnerable neighbourhoods, 35% of the residents use the public healthcare system with people under 60 years of age being the most common users.

The government of the autonomous city of Buenos Aires provided data from 85 level-I public health centers: 46 health and community care centers (CESACs acronym in Spanish), and 39 neighbourhood medical centers (CMBs acronym in Spanish). CESACs are located in neighbourhoods with social and economic vulnerabilities and provide office and home visits as well as community work. CMBs, on the other hand, are located in less vulnerable neighbourhoods and do not perform territorial work.

Each center accounts for 1-8 nuclear teams. Lead by a primary care physician, the make-up of these teams vary by team but all are multidisciplinary and include other health care professionals such as pediatricians, gynecologists, dentists, nurses, health promoters, social workers, and psychologists. Each team provides healthcare to an average of 700 families. However, most people attending these clinics are children and women of childbearing age.

The data presented here represents all visits conducted by the public health centers regardless of the type of health professional seeing the patient as it was not possible to confine the visits just to primary care physicians. The volume of visits used as the denominator to calculate the rates from 2018-2021 was provided by the Argentinean government and counted for a total of 4,439,955 visits. At the beginning of the outbreak, some clinics closed completely. Some professionals provided care with their cell phones without formally documenting visits, while others performed home visits documenting them on paper. Coding for virtual visits was inconsistently applied by providers. Therefore virtual visits with the onset of the pandemic are likely underreported. As the consultation modality (virtual or in-person) was not uniformly reported, we considered virtual visits all encounters with a text description compatible with telephone visits, such as “telephone communication with a patient for surveillance of diabetes”. Only visits with diagnoses or symptoms were included in the numerator. Visits labelled to be just with a specific health care worker (ie. visit with a psychotherapist) but with no diagnosis, were excluded.

Although Argentina uses ICD-10 codes, the use of these codes by primary care healthcare providers was variable and was not readily accessible to us. Therefore, manual mapping of the free text data for the reason for visit to ICD-10 codes was performed.

Australia

The Australian Patron Repository [3] yields administrative, demographic, clinical, prescribing, and pathology data. Billing codes are linked to associated Medical Benefit Service (MBS) item numbers that align with the general practice service provided. Generally, MBS items for visits are based on categories of general practitioner attendance as determined by presentation type and time. Diagnosis codes used in Patron are coded using SNOMED mapping. Diagnosis codes align to condition, past-history, reason for prescription, and reason for visit. This analysis was confined to the reason for visit diagnosis codes. There are specific MBS items for certain procedures, such as cervical smear testing, care planning, including chronic disease and mental health, as well as immunization, and minor surgical procedures, these MBS items provide remuneration based on the procedure type. Three software providers contribute to the Patron database (Medical Director, Best Practice, and Zed-Med) hence the different terms for 'diagnosis' are harmonized/mapped within the Patron database.

Australian data was sourced from 113 general practices (129 sites, accounting for practices with centralized servers) in Victoria, Australia. Aggregation of the reason for visit codes was performed by linking a database of encounters with reasons for visit according to alphanumeric patient identifiers and time/date fields. The reason for visit data was not widely captured by all electronic medical record (EMR) software in a systematic manner (i.e. using a coding system) so for encounters where a SNOMED CT code was not attached, reason for visit coding was performed by matching free text fields from the EMR to an existing database of SNOMED CT codes. Free text terms that were not mapped during this initial step were ordered by frequency of occurrence in the free text reason for encounter field. Each text string was then searched in the SNOMED CT database and mapped to the most appropriate diagnostic code. This coding process was validated by a second coder. In the case where diagnosis codes were not mapped to SNOMED in the database, SNOMED data that aligned with the diagnosis term was manually added prior to data analysis. All encounters in the dataset continued to visit type (face-to-face, telephone, video conference) including encounters not assigned a specific reason for the visit. Visit type (face-to-face, telephone, video conference) was determined by associated Medicare Benefits Schedule (MBS) item numbers. Encounters were linked to a database of processed MBS items by alphanumeric patient identifiers and time/date fields, then aggregated by week and month. Finally, common reasons for visits and sexual and reproductive health codes were identified and aggregated using a combination of predetermined SNOMED CT codes and free text searching to identify any additional relevant encounters or exclude encounters where free text indicated it should be removed. Additionally, the following non-specific reason for visit SNOMED CT codes were removed when calculating the top 10 reasons for visits: active immunization, B12 injection, and PCR test for SARS.

Canada

In Ontario, Canada diagnosis codes are for billing purposes, each billing service code must be accompanied by a diagnosis code with the exception of a specific service code for diabetes and for complete physicals. In these situations, the appropriate diagnosis code is attributed to the service code. The Ontario Health Insurance Plan (OHIP) [4] uses an ICD8/9 hybrid billing code system. The appearance of a specific diagnosis code does not necessarily confer the presence of a disease condition as diagnosis codes can be used when working up a patient for a condition. The diagnosis code is intended to represent the reason for visit however patients often present to the family doctor for many reasons in the same visit and usually, only one diagnosis code is entered per visit. Therefore, there is a tendency to recurrently use commonly billed diagnosis codes and there is likely under coding of less consequential conditions. Data here comes from the University of Toronto Practice-Based Research Network (UTOPIAN) EMR database.

China

The data from China comes from the University of Hong Kong Shen Zhen Hospital (HKUSZH) General Practice Department [5]. At HKUSZH, most patients seen in the General Practice Department would be given ICPC-2 codes for each visit. However, if the patient was seen by specialist departments prior to being seen in the General Practice Department, the general practitioner may have copied the relevant ICD-10 codes that were used by the specialists. The ICD-10 codes were converted to the relevant ICPC-2 codes during the data-cleaning process. Doctors were encouraged to code all the relevant codes for their patients and the average number of reasons per visit per patient was 1.633 in 2019 and 1.77 in 2020. The codes do not necessarily confirm diagnosis as some are symptom-based codes and the code may depend on the physician's judgment. There were no procedure codes recorded; patients would be given a separate ICPC-2 code related to procedures, such as "preventative health" for vaccinations. The codes are used for recording purposes only and are not required for payment.

Prior to October 2020, some primary care physicians were less familiar with the coding system as such there was a code that was treated as an "others" type of code where physicians could put in free text for the reason for visit. During the data-cleaning phase, one research assistant reassigned each "others" code to an ICPC-2 code by reading the first free-text reason for visit provided. Although it was possible that there was more than one free text entry, it was not feasible to code every free text entry. If the patient had an "others" code as well as other ICPC-2 codes, they were included in the analysis. After October 2020, the coding system within the General Practice Department changed such that the "others" code was eliminated. The primary care physicians coded all the relevant ICPC-2 codes for each visit after this change. In addition, around January 2020, in anticipation of this change, the primary care physicians were taught the

ICPC-2 coding system during a quality improvement exercise which may have caused an increase in some codes during this time.

In December 2020, due to the border closures between Hong Kong and Shenzhen arising from the pandemic [6], an estimated 18,000-38,000 Hong Kong permanent residents were unable to return to Hong Kong to see their regular doctors at the public clinic and hospitals run by the Hospital Authority (HA) of Hong Kong. In response to this, the Hong Kong Government decided to set up a “Special Support Scheme for Hosptial Authority chronic disease patients living in the Guangdong Province to sustain consultation under Coronavirus disease-2019” (the “Support Scheme”) [7] under which the General Practice Department was one of the clinical services made available to eligible patients at HKUSZH. Under the Support Scheme, each eligible patient would receive RMB 2,000 per year (equivalent to US$ 300) which could be used to pay for the outpatient investigations and medications at the HKUSZH Hospital Authority clinics specially set up for such purpose. Initially, the Support Scheme was to expire on July 31, 2021, then to May 2023. Therefore, in December 2020, there was a huge rise in the number of patients but due to capacity issues and other visit quotas, such as those reserved for mental health problems, were reduced. Last but not least, in May 2021, there was a management change in the hospital such that the antenatal and postnatal clinics were changed from being under the care of General Practice Department to being under the care of the obstetrics department.

Norway

Norway has a government-run public health care system with a National Health Insurance scheme for all citizens. The treating physician will send reimbursement claims from all consultations to the Norwegian Health Economics Administration (HELFO) [8] and the claims make up the *Control and Payment of Hegand Payment of Health Reimbursement (KUHR)* Database. In addition to the appropriate billing codes, each claim must include one or several diagnosis codes according to the International Classification of Primary Care 2nd version (ICPC-2). For most visits only one diagnosis code will be used, even when there are several reasons for visits and the applied code is at the discretion of the treating physician. Data here cover the whole Norwegian population of 5.38 million (2020). Norwegian data were provided from the project ‘COVID-19 outbreak in Norway – Epidemiology, health care utilization and primary care management – CONOPRI’, supported by a grant from the Trond Mohn Foundation (Grant no. TMS2020TMT06). There has been no formal validation of diagnosis, but a study from a small sample of Norwegian general practices found good correspondence between the patient record notes and the diagnosis for 85 % of visits [9]. In Norway the ICPC-code A97 (no disease) is often used for simple contacts, not visits, when other health professionals in the practice perform procedures that are compensated, since they don't diagnose conditions. One limitation in the data is that the system often will generate this code for virtual contacts and consultations, and

has to be changed by the primary care physicians in order to give the correct reason for contact and consultations. This means that A97 will identify visits when linked with the relevant billing code, but there is no way we can infer what the reason for visit was, therefore this code has been disregarded in the analysis for the reason for visit but they remain in the denominator as a visit.

Peru

The information from Peru included all codes of encounters generated during visits to public sector primary care establishments nationwide. These establishments are dependent on the Ministry of Health and regional governments and are financed by the Comprehensive Health Insurance (SIS acronym in Spanish), and it is the largest health service delivery system in Peru with the largest primary care network. Almost 70% of the national population belongs to this system including those considered as poor population and informal workers. Primary care establishments, according to Peruvian regulations [10], are those dedicated to meeting the health needs of the individual, the family, and the community, mainly on an outpatient basis, and are divided into 4 categories (I-1, I-2, I-3 and I-4), so the information from national hospitals, institutes or clinics are not included in our study.

The public sector's primary care network comprises over 8,263 establishments nationwide, and more than half of these (52.5%) operate without a physician, instead relying on other healthcare providers such as nurses and midwives. Additionally, formal postgraduate training in Family and Community Medicine is not required for primary care practice in Peru. Therefore, there is still a small number of physicians specializing in family and community medicine. Besides primary care physicians, other healthcare providers such as dentists, nurses, midwives, and other allied health professionals provide services at these centers. Notably, the primary care facilities of the public sector have traditionally focused on prioriting maternal and child health problems due, among other factors, to their user population. In contrast, other health service provider systems like Social Security prioritize care for non-communicable diseases such as cardiovascular diseases or diabetes because their population is usually middle-aged or elderly who have or had a formal job.

In Peru, two coding systems are used to record the activities and encounters that occur during visits to healthcare establishments. The ICD-10 CM system is used to record all diseases or health conditions (from Chapter A to Y), while the CPT (Current Procedural Terminology) system is used to record all activities or procedures performed. The ICD-10 CM dataset was obtained from the National Repository of Health Information [11] (REUNIS acronym in Spanish), which is the Ministry of Health's portal that collects health statistics from various entities within the Peruvian health system. While most primary care visits were recorded using this system, some visits related to mental health, gynecology, and general medical exams (that correspond to Chapter Z of ICD-10 CM) were captured using CPT codes. The CPT dataset was provided by the Ministry of Health through the Standard Transparency Portal (PTE) and was

converted to ICD-10 CM codes based on their text descriptions. Codes that did not belong to chapter Z of the ICD-10 CM system, such as surgeries and procedures, were excluded. Because of the characteristics of the Peruvian healthcare system described above, it was not feasible to separate visits conducted by primary care physicians from those carried out by other primary care professionals in either dataset (ICD-10 CM or CPT). The total visit denominator was computed by summing all the visits associated with an ICD-10 CM code. However, since some patients could have multiple codes assigned during a single visit, this denominator might have overestimated the true number of visits. Similarly, in the CPT dataset, we were unable to distinguish between virtual and in-person visits, which might have resulted in an undercounting of the total number of virtual visits. Additionally, data from Peru was not available in 2018.

Singapore

In Singapore, ICD-10 AM diagnosis codes are used for inpatient billing and outpatient claims for government subsidies of selected chronic diseases. ICD-10 AM codes are also used widely in the private sector for claims to medical benefits administrators and insurance companies. Diagnosis codes indicate the presence of a disease condition and are usually entered after the diagnosis is made clinically. In the polyclinic setting, the number of claimable codes is limited, resulting in a loss of specificity and clinicians using commonly used diagnosis codes. This results in the under-coding of conditions outside the claimable list. Most polyclinic patients had their appointments deferred during COVID and were followed up by phone without cost (hence not captured in the data presented here). Data here comes from the National University Polyclinics (NUP) Data Mart [12], which transforms data from the finance systems for analytics use.

Sweden

Swedish health care is heavily decentralized, and health services are mainly provided by the 21 regions that are allowed to levy taxes to fund health care services. The regions provide hospital services as well as primary care services, both by public and private providers [13].

Almost all care is publicly funded through the tax system and additional patient fees. Patients' out-of-pocket fee payments are 2-3% of the total funding, it is however capped at approximately 115 Euros a year (1200 SEK), and medications are also capped at 230 Euros a year (2600 SEK).

The data from Sweden is from Uppsala, one of the regions where the treating general practice is paid by capitation (approximately 70%), as well as per visit. The claims are automatic and generated through numbers of visits, physical, video-based, and telephone but also weighted by

CNI (Care Need Index). The visits are coded using ICD-10 codes. There were no extra reimbursements for coding in general and during the period of the study and no additional programs paying for certain codes were running.

The Uppsala region has a population of 385,000 people (2020). The data provided included visits to primary care centers and was partially coded. Healthcare providers at the centers are not exclusively primary care physicians. However, primary care physicians are more likely to code their visits. To determine the reasons for the visit counts and rates we only used coded visits (approximately 25% of total visits). However, when displaying total visits and separating them into virtual and in-person encounters, we included all recorded visits even if they were uncoded, which is more representative of the volume of primary care services patients received.

United States

Data from the USA come from DARNet Institute [14] primary care clinics in California, Texas, and Colorado. Visits to primary care were identified through the physicians' or site's specialty (from National Provider Identifiers (NPI)'s, only family medicine and general internal medicine physician visits were included. Current Procedural Terminology (CPT) codes were used to indicate whether the visit was virtual or in-person. From 2018-2021, primary care visits were coded using ICD-9, ICD-10, or ICD-10 CM codes. Clinics varied in their transition time from ICD-9 to ICD-10. As such, it was necessary to map ICD-9 to ICD-10 codes and confine ICD-10 and ICD-10 CM codes to the first 2 digits of the ICD-10 coding system. Patients typically get on average 3.2 diagnoses per visit in primary care and reasons of importance are not ranked. Therefore, we used all diagnosis codes available for each patient and each visit. In our data, the average number of codes/categories per visit was 2.91. Additional procedure codes were not included in this analysis and codes for immunization, screening mammogram, and colon cancer screening were not included in the top 10 reasons for visits.

References

1. Rubinstein A, Zerbino MC, Cejas C, López A. Making Universal Health Care Effective in Argentina: A Blueprint for Reform. Health systems and reform. 2018; 4(3), 203–213. doi: 10.1080/23288604.2018.1477537
2. General Directorate of Statistics and Censuses. Percentage distribution of the population by type of medical coverage according to age group and area. Buenos Aires city. Years 2011/2022. Government of the City of Buenos Aires. [Cited 2023 Mar 1]. Available from: https://www.estadisticaciudad.gob.ar/eyc/?p=83870
3. Boyle D, Sanci L, Emery J, Gunn J, Hocking J, Manski-Nankervis JA, et al. PATRON Primary Care Research Data Repository. [Cited 2023 Mar 1]. Available from: https://researchdata.edu.au/patron-primary-care-research-repository/1361016
4. Ontario Ministry of Health. Understanding Health Care in Ontario. [Cited 2023 Mar 26]. Available from : https://www.health.gov.on.ca/en/ministry/hc_system/
5. The University of Honk Kong-Shenzhen Hospital Department of Family Medicine. 2023. [Cited 2023 Mar 26]. Available from: http://www.hku-szh.org/en/ClinicalServices/ClinicalServices/DepartmentofFamilyMedicine/
6. Crisis24. China: Authorities to tighten commercial COVID-19 restrictions Dec. 10-23 /update 42. 2020. [Cited 2023 Mar 26]. Available from : https://crisis24.garda.com/alerts/2020/12/china-authorities-to-tighten-commercial-covid-19-restrictions-dec-10-23-update-42
7. Hospital Authority. HKSAR Government Special Support Scheme for Hospital Authority Chronic Disease Patients Living in the Guangdong Province to Sustain Their Medical Consultation under Coronavirus Disease-2019. [Cited 2023 Mar 26]. Available from: https://www.ha.org.hk/visitor/ha_visitor_index.asp?Content_ID=257766&Lang=ENG
8. Tikkanen R, Osborn R, Mossialos E, Djordjevic A, Wharton GA. International Health Care System Profiles Norway. The Commonwealth Fund. 2020. [Cited 2023 Mar 26]. Available from: https://www.commonwealthfund.org/international-health-policy-center/countries/norway
9. Sporaland GL, Mouland G, Bratland B, Rygh E, Reiso H. General practitioners' use of ICPC diagnoses and their correspondence with patient record notes. Tidsskr Nor Laegeforen. 2019 Oct 14;139(15). Norwegian, English. doi: 10.4045/tidsskr.18.0440.
10. Peruvian Government. Services and categories of the first level of health care. [Cited 2023 Mar 26]. Available from: https://www.gob.pe/16728-servicios-y-categorias-del-primer-nivel-de-atencion-de-salud
11. Peruvian Ministry of Health. REUNIS Single National Repository of Health Information. [Cited 2023 Mar 26]. Available from: https://www.minsa.gob.pe/reunis/
12. National University Policlinics. About NUP. 2022. [Cited 2023 Mar 26]. Available from: https://www.nup.com.sg/Pages/About%20Us/about-us-corporate-profile.aspx

13. Tikkanen R, Mossialos E, Djordjevic A, Wharton GA. International Health Care System Profiles Sweden. The Commonwealth Fund. 2020. [Cited 2023 Mar 26]. Available from: https://www.commonwealthfund.org/international-health-policy-center/countries/sweden
14. DARNet Institute. Informing Practice: Improving Care. DARTNet Institute. [Cited 2023 Mar 26]. Available from: https://dartnet.info/default.htm
