# Supplementary material for: Changes in reasons for visits to primary care after the start of the COVID-19 pandemic: An international comparative study by the International Consortium of Primary Care Big Data Researchers (INTRePID)
Source: PLOS Glob Public Health. 2024 Aug 22;4(8):e0003406. doi: 10.1371/journal.pgph.0003406 (PMC11341054; doi:10.1371/journal.pgph.0003406)
Supplement: S2 Data — (PDF) [file pgph.0003406.s011.pdf]

S2 Data. Monthly visits by modality of care

Country	Year	Month	In-person visits	Virtual visits
**Argentina**	**2018**	January	72,979	74
		February	63,222	33
		March	88,211	55
		April	95,481	53
		May	105,021	50
		June	90,818	98
		July	86,988	129
		August	108,158	97
		September	94,664	77
		October	113,782	90
		November	93,323	113
		December	70,229	76
**Argentina**	**2019**	January	93,722	123
		February	91,602	83
		March	100,577	114
		April	110,707	67
		May	135,225	78
		June	108,425	83
		July	109,338	60
		August	112,344	130
		September	121,268	80
		October	125,798	118
		November	108,226	80
		December	84,007	48
**Argentina**	**2020**	January	112,303	108
		February	88,985	87
		March	81,940	141
		April	66,774	987
		May	69,142	1,525
		June	75,339	2,032
		July	74,603	2,100
		August	74,216	2,017
		September	84,953	2,348
		October	85,171	1,808
		November	68,053	2,183
		December	62,144	1,300
**Argentina**	**2021**	January	75,679	1,124
		February	64,020	881
		March	92,784	1,272
		April	104,660	1,378
		May	101,197	1,300
		June	103,401	1,412
		July	89,887	1,201
		August	96,693	1,033
		September	100,937	1,205
		October	85,057	973
		November	95,359	814
		December	70,866	439

S2 Data. Monthly visits by modality of care (continued)

Country	Year	Month	In-person visits	Virtual visits
**Australia**	**2018**	January	98,333	0
		February	106,238	0
		March	112,707	0
		April	111,840	0
		May	147,909	0
		June	115,057	0
		July	121,676	0
		August	126,786	0
		September	108,586	0
		October	125,150	0
		November	120,725	0
		December	102,859	0
**Australia**	**2019**	January	114,393	0
		February	120,057	0
		March	123,657	0
		April	124,873	0
		May	165,714	0
		June	131,053	0
		July	147,489	0
		August	145,830	0
		September	132,497	0
		October	148,192	0
		November	132,334	0
		December	124,737	0
**Australia**	**2020**	January	127,818	0
		February	122,473	<5
		March	125,797	18,593
		April	93,979	68,879
		May	89,792	64,693
		June	88,696	62,492
		July	75,286	84,765
		August	57,359	88,777
		September	70,039	81,823
		October	79,835	72,132
		November	89,967	59,341
		December	91,687	48,581
**Australia**	**2021**	January	88,471	42,294
		February	97,077	50,899
		March	116,899	51,354
		April	114,791	40,374
		May	125,978	46,862
		June	105,177	51,827
		July	103,390	51,494
		August	100,268	61,407
		September	89,199	60,618
		October	85,881	65,674
		November	94,153	55,001
		December	86,504	46,984

Note: Counts of less than 5 are represented as "<5" in order to maintain patient confidentiality and prevent potential identification of individuals.

S2 Data. Monthly visits by modality of care (continued)

Country	Year	Month	In-person visits	Virtual visits
**Canada**	**2018**	January	76,277	0
		February	64,501	0
		March	71,172	<5
		April	70,172	<5
		May	75,233	<5
		June	69,163	<5
		July	67,977	8
		August	67,249	<5
		September	64,500	<5
		October	77,417	<5
		November	74,966	<5
		December	61,596	<5
**Canada**	**2019**	January	75,569	<5
		February	60,450	<5
		March	70,243	<5
		April	75,045	<5
		May	76,466	7
		June	68,298	<5
		July	74,138	10
		August	66,651	<5
		September	72,227	6
		October	78,547	11
		November	74,803	10
		December	65,549	12
**Canada**	**2020**	January	79,434	15
		February	65,382	21
		March	38,495	28,587
		April	6,658	51,501
		May	8,977	50,882
		June	13,257	57,325
		July	17,954	53,013
		August	19,901	48,064
		September	22,980	53,046
		October	24,837	54,685
		November	23,686	53,416
		December	18,572	50,477
**Canada**	**2021**	January	17,236	57,231
		February	17,028	52,879
		March	23,983	65,016
		April	19,854	58,980
		May	20,536	55,926
		June	25,841	55,272
		July	27,246	45,729
		August	29,446	43,304
		September	32,724	43,613
		October	36,201	40,612
		November	41,973	41,332
		December	29,218	34,048

Note: Counts of less than 5 are represented as "<5" in order to maintain patient confidentiality and prevent potential identification of individuals.

S2 Data. Monthly visits by modality of care (continued)

Country	Year	Month	In-person visits	Virtual visits
**China**	**2018**	January	8,272	0
		February	5,743	0
		March	8,461	0
		April	7,842	0
		May	7,701	0
		June	7,173	0
		July	7,612	0
		August	7,573	0
		September	7,054	0
		October	6,577	0
		November	7,708	0
		December	7,432	0
**China**	**2019**	January	7,197	0
		February	4,863	0
		March	7,112	0
		April	6,954	0
		May	6,629	0
		June	6,422	0
		July	7,050	0
		August	6,507	0
		September	5,823	0
		October	5,566	0
		November	6,922	0
		December	7,516	0
**China**	**2020**	January	5,502	0
		February	1,834	0
		March	3,244	0
		April	3,926	0
		May	5,318	0
		June	4,774	0
		July	5,128	0
		August	6,321	0
		September	6,137	0
		October	5,954	0
		November	7,104	0
		December	8,133	0
**China**	**2021**	January	8,302	0
		February	6,369	0
		March	9,099	0
		April	7,686	0
		May	7,153	0
		June	6,128	0
		July	7,490	0
		August	7,292	0
		September	6,985	0
		October	6,275	0
		November	6,919	0
		December	7,189	0

S2 Data. Monthly visits by modality of care (continued)

Country	Year	Month	In-person visits	Virtual visits
**Norway**	**2018**	January	1,367,096	11,687
		February	1,176,205	12,039
		March	1,126,818	13,174
		April	1,235,284	16,038
		May	1,172,707	17,505
		June	1,206,555	19,340
		July	809,125	10,856
		August	1,153,165	18,299
		September	1,161,879	22,855
		October	1,344,738	30,338
		November	1,322,843	33,127
		December	1,011,667	28,814
**Norway**	**2019**	January	1,236,736	22,060
		February	1,088,771	21,979
		March	1,236,345	27,832
		April	1,108,590	28,234
		May	1,207,313	33,309
		June	1,129,090	34,988
		July	868,578	22,086
		August	1,125,910	34,194
		September	1,248,612	45,871
		October	1,346,811	54,350
		November	1,273,137	57,290
		December	1,072,804	53,272
**Norway**	**2020**	January	1,349,476	42,451
		February	1,148,835	41,037
		March	891,235	467,729
		April	651,527	427,794
		May	833,743	317,849
		June	1,020,992	263,797
		July	726,396	171,014
		August	915,847	250,434
		September	977,981	274,048
		October	1,117,283	303,496
		November	1,026,903	379,875
		December	870,107	331,045
**Norway**	**2021**	January	917,154	323,865
		February	893,268	283,508
		March	1,038,107	363,648
		April	952,941	304,242
		May	938,188	290,491
		June	1,097,687	302,134
		July	677,242	184,412
		August	968,339	267,484
		September	1,140,424	351,463
		October	1,092,944	312,276
		November	1,209,006	383,260
		December	898,586	383,119

S2 Data. Monthly visits by modality of care (continued)

Country	Year	Month	In-person visits	Virtual visits
**Singapore**	**2018**	January	110,794	0
		February	90,882	0
		March	103,725	0
		April	104,175	0
		May	105,252	0
		June	94,869	0
		July	110,859	0
		August	102,364	0
		September	96,807	0
		October	110,336	0
		November	103,284	0
		December	95,733	0
**Singapore**	**2019**	January	110,116	0
		February	90,011	0
		March	106,283	0
		April	109,090	0
		May	109,581	0
		June	96,532	0
		July	118,571	0
		August	103,372	0
		September	100,665	<5
		October	108,034	<5
		November	106,030	9
		December	101,552	7
**Singapore**	**2020**	January	104,915	10
		February	90,993	19
		March	90,259	11
		April	63,008	16
		May	60,530	10
		June	82,060	12
		July	84,650	11
		August	83,390	17
		September	87,362	18
		October	85,903	14
		November	67,840	<5
		December	67,195	26
**Singapore**	**2021**	January	79,110	38
		February	72,784	28
		March	92,367	48
		April	86,020	60
		May	73,883	94
		June	80,212	144
		July	84,998	102
		August	83,139	236
		September	82,959	381
		October	80,492	1,464
		November	82,471	472
		December	84,976	585

Note: Counts of less than 5 are represented as "<5" in order to maintain patient confidentiality and prevent potential identification of individuals.

S2 Data. Monthly visits by modality of care (continued)

Country	Year	Month	In-person visits	Virtual visits
**Peru**	**2019**	January	3,850,264	85
		February	3,819,448	85
		March	4,217,017	151
		April	4,184,448	79
		May	4,230,826	75
		June	4,447,366	74
		July	4,435,316	58
		August	4,439,760	265
		September	4,858,767	621
		October	4,782,434	708
		November	4,859,600	1,216
		December	4,377,318	2,810
**Peru**	**2020**	January	4,752,601	1,822
		February	4,708,434	3,141
		March	3,153,278	5,786
		April	1,134,291	65,088
		May	1,366,055	139,616
		June	1,688,474	183,933
		July	1,918,958	197,852
		August	2,102,672	214,509
		September	2,438,993	229,630
		October	2,860,422	223,266
		November	3,063,481	199,830
		December	2,970,181	182,792
**Peru**	**2021**	January	3,067,472	174,768
		February	2,885,350	208,813
		March	3,485,795	226,332
		April	3,380,210	224,998
		May	3,295,354	219,465
		June	3,499,928	204,767
		July	3,720,252	211,432
		August	4,136,246	214,762
		September	4,306,617	220,766
		October	4,408,104	214,291
		November	4,606,850	217,651
		December	4,185,634	194,993

S2 Data. Monthly visits by modality of care (continued)

Country	Year	Month	In-person visits	Virtual visits
**Sweden**	**2018**	January	99,348	46,379
		February	91,678	41,902
		March	99,498	44,363
		April	95,351	40,824
		May	104,700	42,922
		June	86,278	36,178
		July	67,364	29,559
		August	90,251	37,421
		September	94,754	39,066
		October	110,610	46,056
		November	105,892	42,500
		December	78,241	34,826
**Sweden**	**2019**	January	103,409	45,340
		February	95,182	41,443
		March	103,115	43,155
		April	99,403	40,433
		May	104,168	41,408
		June	85,217	34,514
		July	71,516	31,886
		August	88,096	37,675
		September	104,189	41,602
		October	115,757	45,626
		November	106,248	42,396
		December	86,823	36,382
**Sweden**	**2020**	January	105,320	44,012
		February	99,392	41,503
		March	85,425	57,002
		April	61,777	57,940
		May	65,705	52,594
		June	72,188	53,137
		July	55,306	41,232
		August	69,441	45,386
		September	86,530	53,234
		October	86,275	54,776
		November	80,216	55,621
		December	67,140	51,217
**Sweden**	**2021**	January	69,568	49,891
		February	72,211	50,765
		March	87,799	60,983
		April	77,724	53,759
		May	81,221	54,301
		June	84,243	53,956
		July	56,978	40,628
		August	71,389	47,289
		September	90,221	55,260
		October	89,145	56,681
		November	91,085	58,549
		December	75,939	53,480

S2 Data. Monthly visits by modality of care (continued)

Country	Year	Month	In-person visits	Virtual visits
**United States**	**2018**	January	22,099	0
		February	19,134	0
		March	19,984	0
		April	19,754	0
		May	20,420	0
		June	18,710	0
		July	18,862	0
		August	19,907	0
		September	17,242	0
		October	20,920	0
		November	18,432	0
		December	16,548	0
**United States**	**2019**	January	19,748	0
		February	17,720	0
		March	17,682	0
		April	18,281	0
		May	18,157	0
		June	16,212	0
		July	17,757	0
		August	17,869	0
		September	17,300	0
		October	20,251	0
		November	16,846	0
		December	17,220	0
**United States**	**2020**	January	19,954	<5
		February	17,774	0
		March	13,855	1,272
		April	6,032	6,569
		May	8,829	4,753
		June	12,712	4,223
		July	13,069	4,122
		August	12,781	3,475
		September	14,491	3,054
		October	14,828	3,191
		November	13,041	3,354
		December	12,123	3,810
**United States**	**2021**	January	5,901	870
		February	5,444	817
		March	6,805	890
		April	15,273	2,563
		May	14,403	2,043
		June	15,739	1,686
		July	14,623	1,527
		August	15,575	1,830
		September	15,243	1,842
		October	15,645	1,746
		November	15,108	1,678
		December	15,224	2,034

Note: Counts of less than 5 are represented as "<5" in order to maintain patient confidentiality and prevent potential identification of individuals.
