# Supplementary material for: Changes in reasons for visits to primary care after the start of the COVID-19 pandemic: An international comparative study by the International Consortium of Primary Care Big Data Researchers (INTRePID)
Source: PLOS Glob Public Health. 2024 Aug 22;4(8):e0003406. doi: 10.1371/journal.pgph.0003406 (PMC11341054; doi:10.1371/journal.pgph.0003406)
Supplement: S3 Data — (PDF) [file pgph.0003406.s012.pdf]

S3 Data. Monthly visits for common conditions

Country	Year	Month	Anxiety/Depression	Cough, Cold, or Acute Respiratory Infection	Preventative Care	Diabetes	Hypercholesterolemia	Hypertension
**Argentina**	**2018**	January	1,731	1,115	23,693	1,455	379	1,506
		February	1,337	1,177	22,442	1,187	303	1,214
		March	1,739	2,988	30,799	1,609	430	1,657
		April	1,918	2,411	32,966	1,637	445	1,624
		May	1,954	3,882	32,034	1,933	567	1,759
		June	1,864	4,483	26,039	1,746	523	1,648
		July	1,910	3,488	26,349	1,880	498	1,789
		August	2,117	5,209	31,938	2,060	567	2,052
		September	1,808	4,697	25,767	1,740	481	1,719
		October	2,372	3,502	31,350	2,071	577	2,012
		November	2,005	2,193	25,121	2,061	553	1,785
		December	1,606	1,280	19,865	1,789	484	1,640
**Argentina**	**2019**	January	2,128	1,204	27,047	2,068	566	2,150
		February	2,019	1,506	28,231	1,894	480	1,913
		March	1,873	2,571	31,895	1,992	505	2,033
		April	2,060	3,273	33,173	1,967	581	1,945
		May	2,421	4,553	34,479	2,410	674	2,404
		June	2,018	4,915	24,573	2,149	606	1,954
		July	2,102	4,522	24,841	2,336	684	2,170
		August	2,224	3,357	26,722	2,553	788	2,402
		September	2,161	3,692	27,715	2,822	806	2,683
		October	2,347	2,695	28,659	3,006	802	2,780
		November	2,087	2,141	24,842	2,643	770	2,441
		December	1,525	1,526	18,628	2,170	647	1,934
**Argentina**	**2020**	January	1,998	1,392	26,509	3,003	862	2,765
		February	1,541	1,348	20,927	2,192	625	2,012
		March	1,298	2,052	16,108	2,162	450	1,687
		April	1,128	522	4,357	2,260	264	1,538
		May	1,504	417	4,593	2,086	288	1,506
		June	1,700	446	5,478	2,498	369	1,813
		July	1,785	372	5,871	2,531	407	1,738

S3 Data. Monthly visits for common conditions (continued)

Country	Year	Month	Anxiety/Depression	Cough, Cold, or Acute Respiratory Infection	Preventative Care	Diabetes	Hypercholesterolemia	Hypertension
**Argentina**	**2020**	August	1,953	367	6,970	2,821	550	1,940
		September	1,926	437	9,282	3,112	551	2,040
		October	1,904	359	11,202	3,103	648	2,004
		November	1,474	379	10,407	2,868	626	1,989
		December	1,523	298	10,398	2,736	586	1,935
**Argentina**	**2021**	January	1,459	336	12,532	3,015	672	2,099
		February	1,290	341	11,277	2,398	593	1,655
		March	2,017	1,347	17,630	3,562	823	2,401
		April	2,090	955	17,433	3,632	784	2,230
		May	1,963	930	16,145	3,714	784	2,193
		June	2,293	1,065	17,975	4,052	863	2,382
		July	2,086	1,422	16,627	3,213	724	2,059
		August	2,173	2,815	18,152	3,193	806	2,087
		September	2,103	3,527	19,547	3,428	870	2,119
		October	1,730	3,172	16,397	2,756	760	1,856
		November	1,798	2,537	19,002	3,402	912	2,302
		December	1,526	795	13,925	2,925	745	1,766
**Australia**	**2018**	January	2,290	2,107	1,098	1,542	532	1,826
		February	2,671	2,660	1,196	1,852	703	2,313
		March	2,806	3,476	1,350	1,811	734	2,291
		April	2,598	3,619	1,207	1,839	655	2,294
		May	3,044	6,268	1,490	2,016	861	2,741
		June	2,521	5,939	1,182	1,651	700	2,322
		July	2,791	6,302	1,343	1,913	694	2,565
		August	2,808	6,318	1,613	1,823	748	2,783
		September	2,413	5,560	1,274	1,681	608	2,305
		October	2,900	4,623	1,376	2,028	722	2,682
		November	2,691	4,326	1,416	2,023	667	2,567
		December	2,331	3,201	1,156	1,788	637	2,374
**Australia**	**2019**	January	2,739	2,553	1,337	2,001	653	2,266
		February	2,794	3,521	1,489	2,112	701	2,455
		March	2,960	4,235	1,518	2,107	846	2,727

S3 Data. Monthly visits for common conditions (continued)

Country	Year	Month	Anxiety/Depression	Cough, Cold, or Acute Respiratory Infection	Preventative Care	Diabetes	Hypercholesterolemia	Hypertension
**Australia**	**2019**	April	2,661	4,968	1,436	1,912	696	2,452
		May	3,051	7,966	1,631	2,189	837	2,772
		June	2,631	8,189	1,465	1,772	670	2,449
		July	3,083	8,406	1,613	2,075	746	2,958
		August	3,355	9,015	1,664	1,945	796	3,050
		September	2,922	7,645	1,634	1,930	771	2,748
		October	3,592	6,816	1,769	2,328	771	3,117
		November	3,096	5,207	1,562	1,962	757	2,827
		December	2,908	4,567	1,517	1,961	751	2,755
**Australia**	**2020**	January	3,059	3,750	1,598	1,983	817	2,691
		February	2,988	4,376	1,549	2,050	797	2,643
		March	3,268	8,802	1,457	2,195	838	3,102
		April	3,186	2,742	999	2,012	740	2,415
		May	3,409	2,096	1,210	2,188	864	2,709
		June	3,609	3,686	1,500	2,318	1,096	2,966
		July	3,893	3,675	1,390	2,266	1,096	3,064
		August	3,993	1,677	1,188	2,368	1,073	3,090
		September	4,082	1,049	1,481	2,489	1,232	3,198
		October	3,781	1,426	1,620	2,424	1,189	3,115
		November	3,484	1,994	1,730	2,373	1,086	2,871
		December	3,100	1,900	1,712	2,262	1,036	2,955
**Australia**	**2021**	January	3,028	1,583	1,650	2,077	1,019	2,557
		February	3,342	3,083	1,741	2,323	1,062	2,771
		March	3,901	5,232	1,777	2,622	1,294	3,272
		April	3,310	4,542	1,711	2,268	1,079	2,913
		May	3,394	5,751	1,706	2,323	1,189	3,000
		June	3,560	3,537	1,506	2,365	1,154	3,012
		July	3,650	3,831	1,503	2,413	1,205	3,086
		August	3,827	3,658	1,557	2,390	1,166	3,113
		September	3,545	1,678	1,492	2,295	1,158	2,926
		October	3,700	1,436	1,388	2,225	1,184	2,893
		November	3,430	2,333	1,532	2,291	1,053	2,920
		December	2,837	2,274	1,256	2,127	938	2,556

S3 Data. Monthly visits for common conditions (continued)

Country	Year	Month	Anxiety/Depression	Cough, Cold, or Acute Respiratory Infection	Preventative Care	Diabetes	Hypercholesterolemia	Hypertension
**Canada**	**2018**	January	5,548	5,679	3,826	3,986	670	4,078
		February	4,813	4,514	3,098	3,543	605	3,373
		March	5,169	4,130	3,520	4,169	618	4,119
		April	5,171	3,462	3,493	4,100	702	4,185
		May	5,455	3,210	3,872	4,521	752	4,409
		June	5,340	2,544	3,590	4,154	621	3,866
		July	5,016	2,318	3,700	3,891	604	3,935
		August	5,055	1,955	3,965	3,774	558	3,709
		September	4,797	2,580	3,572	4,015	572	3,683
		October	5,820	3,763	4,310	4,633	704	4,517
		November	5,686	3,933	4,226	4,505	732	4,645
		December	4,531	4,286	3,077	3,501	564	3,550
**Canada**	**2019**	January	5,626	4,648	4,071	4,164	639	3,995
		February	4,743	3,017	3,327	3,594	539	3,371
		March	5,395	3,573	3,636	4,466	669	4,089
		April	5,558	3,798	3,871	4,594	673	4,692
		May	5,751	3,472	3,914	4,573	717	4,568
		June	5,148	2,908	3,528	4,180	628	3,933
		July	5,493	2,559	4,032	4,390	686	4,239
		August	4,935	2,101	3,684	3,775	587	3,692
		September	5,368	2,841	3,781	4,807	669	4,240
		October	6,026	3,661	4,168	4,904	762	4,793
		November	5,705	3,829	4,098	4,601	796	4,585
		December	5,053	4,978	3,161	3,884	597	3,787
**Canada**	**2020**	January	5,870	5,451	3,965	4,646	673	4,575
		February	5,193	3,599	3,388	3,876	581	3,821
		March	6,795	5,325	2,359	4,079	657	4,121
		April	7,246	3,115	1,848	2,705	377	3,318
		May	6,589	1,771	1,970	2,938	409	2,891
		June	7,187	1,413	2,139	3,642	596	3,201
		July	6,882	1,329	2,249	3,552	634	3,019
		August	6,591	1,307	2,333	3,164	651	2,852
		September	7,443	1,718	2,452	3,864	928	3,490

S3 Data. Monthly visits for common conditions (continued)

Country	Year	Month	Anxiety/Depression	Cough, Cold, or Acute Respiratory Infection	Preventative Care	Diabetes	Hypercholesterolemia	Hypertension
**Canada**	**2020**	October	7,593	2,142	2,519	4,228	940	3,850
		November	7,709	1,859	2,456	3,979	863	3,787
		December	7,216	1,761	1,969	3,406	784	3,317
**Canada**	**2021**	January	7,835	1,662	2,179	3,703	790	3,596
		February	7,400	1,437	1,967	3,598	736	3,267
		March	9,126	1,840	2,428	4,842	958	4,181
		April	8,257	1,655	2,164	3,938	825	3,546
		May	7,875	1,665	2,147	3,684	789	3,349
		June	8,236	1,491	2,467	4,109	947	3,624
		July	6,955	1,468	2,394	3,631	851	3,025
		August	6,698	1,636	2,671	3,609	819	3,080
		September	7,483	1,973	2,818	4,145	910	3,507
		October	7,687	2,453	3,022	4,218	971	3,803
		November	8,088	2,864	3,245	4,543	1,121	4,150
		December	6,120	2,417	2,288	3,214	824	3,144
**China**	**2018**	January	116	245	249	107	80	223
		February	83	202	159	59	37	120
		March	133	236	282	91	70	204
		April	116	206	258	94	53	142
		May	131	176	271	80	66	169
		June	114	141	109	80	50	155
		July	119	138	110	77	48	165
		August	113	127	140	70	61	140
		September	133	98	99	83	56	161
		October	133	125	109	86	45	157
		November	132	169	139	67	65	210
		December	137	183	136	85	66	204
**China**	**2019**	January	423	281	37	130	144	263
		February	276	173	34	80	92	139
		March	473	300	38	116	154	223
		April	410	236	64	116	155	212
		May	436	173	93	103	121	196

S3 Data. Monthly visits for common conditions (continued)

Country	Year	Month	Anxiety/Depression	Cough, Cold, or Acute Respiratory Infection	Preventative Care	Diabetes	Hypercholesterolemia	Hypertension
**China**	**2019**	June	423	152	173	112	171	202
		July	494	188	258	133	160	209
		August	406	179	257	119	137	216
		September	335	150	230	108	138	197
		October	368	136	239	94	156	202
		November	394	205	445	107	172	218
		December	429	222	919	136	180	275
**China**	**2020**	January	386	164	728	105	141	232
		February	139	48	5	50	27	73
		March	280	51	8	69	61	114
		April	471	74	544	95	128	195
		May	412	32	543	129	145	190
		June	537	42	641	133	178	244
		July	357	27	660	145	182	260
		August	323	36	870	125	171	213
		September	560	67	1,281	161	235	313
		October	356	62	954	154	244	307
		November	426	122	1,152	285	363	507
		December	431	136	1,235	759	106	1,340
**China**	**2021**	January	175	90	977	651	161	1,299
		February	121	59	583	521	99	931
		March	135	52	996	1,020	151	1,325
		April	122	72	290	830	153	1,510
		May	120	95	258	827	189	1,391
		June	92	47	398	680	154	1,200
		July	95	28	684	538	196	1,744
		August	106	24	273	371	129	1,332
		September	81	26	389	383	146	1,598
		October	64	21	368	422	156	1,280
		November	123	29	491	481	183	1,395
		December	110	52	315	425	246	1,540

S3 Data. Monthly visits for common conditions (continued)

Country	Year	Month	Anxiety/Depression	Cough, Cold, or Acute Respiratory Infection	Preventative Care	Diabetes	Hypercholesterolemia	Hypertension
**Norway**	**2018**	January	61,679	83,602	21,045	31,762	7,142	46,234
		February	52,209	72,208	18,878	29,401	6,242	40,205
		March	51,088	56,941	19,530	32,296	6,359	42,260
		April	58,631	46,586	23,337	33,504	7,437	47,928
		May	53,866	44,986	23,239	32,153	6,928	45,040
		June	53,422	38,703	25,054	36,256	7,379	48,861
		July	32,540	26,182	11,479	16,368	2,753	22,247
		August	52,478	31,786	19,833	31,385	5,695	40,505
		September	52,851	48,890	24,510	34,122	6,639	46,354
		October	63,923	52,877	36,363	33,146	7,878	51,593
		November	64,902	60,828	30,673	33,937	7,484	51,096
		December	48,022	55,091	16,811	31,687	5,760	42,245
**Norway**	**2019**	January	58,847	71,694	18,129	29,079	5,978	43,390
		February	50,134	64,820	15,834	27,878	5,448	38,708
		March	59,415	56,625	21,913	34,994	6,680	47,204
		April	53,187	44,792	19,161	29,923	5,701	42,096
		May	55,727	54,506	22,856	33,252	6,747	45,783
		June	52,353	42,479	21,801	36,311	6,590	46,796
		July	35,203	31,203	12,915	18,221	2,918	23,817
		August	52,519	31,941	17,651	31,798	5,449	38,179
		September	58,518	55,106	22,654	37,089	7,018	47,017
		October	65,931	56,853	36,136	34,197	7,781	50,213
		November	64,705	61,830	29,644	33,787	7,510	48,058
		December	52,520	62,141	18,525	33,808	6,123	41,506
**Norway**	**2020**	January	68,769	72,541	22,216	33,137	6,932	46,224
		February	56,698	65,515	19,790	30,635	6,129	40,247
		March	58,845	76,496	17,084	32,078	5,393	38,306
		April	56,031	24,376	10,749	30,291	4,391	35,793
		May	58,632	19,060	16,183	35,028	6,174	44,465
		June	59,858	21,178	24,585	38,923	7,306	49,274
		July	39,328	16,743	11,526	19,137	3,075	23,467
		August	53,324	24,221	17,851	31,810	5,032	35,646
		September	58,051	28,852	23,834	35,844	6,764	43,694

S3 Data. Monthly visits for common conditions (continued)

Country	Year	Month	Anxiety/Depression	Cough, Cold, or Acute Respiratory Infection	Preventative Care	Diabetes	Hypercholesterolemia	Hypertension
**Norway**	**2020**	October	67,191	27,177	58,878	37,334	8,214	51,281
		November	69,569	29,048	36,184	37,808	8,029	48,711
		December	59,890	21,246	20,826	37,277	6,619	43,211
**Norway**	**2021**	January	63,481	21,208	21,097	31,497	6,164	40,750
		February	60,061	19,165	21,724	32,741	6,402	40,002
		March	67,985	20,895	25,071	39,795	7,376	47,302
		April	60,908	15,093	25,010	35,046	7,049	43,199
		May	57,963	15,847	25,953	35,441	6,853	43,380
		June	61,930	19,441	31,148	43,018	7,980	50,774
		July	34,897	15,060	12,107	17,745	2,742	20,813
		August	53,839	23,474	19,367	33,105	5,333	35,909
		September	61,982	56,630	29,207	40,798	7,534	46,912
		October	58,958	74,927	47,813	32,662	7,106	42,605
		November	65,222	101,528	48,144	37,773	8,242	47,793
		December	52,049	56,907	21,430	35,563	6,302	39,251
**Peru**	**2019**	January	48,436	125,424	377,070	52,057	45,519	63,229
		February	48,613	115,005	368,409	49,657	47,940	60,884
		March	54,593	149,458	421,220	53,971	52,835	67,222
		April	51,841	154,249	440,355	52,148	51,195	65,829
		May	53,858	172,407	436,351	52,892	50,837	67,562
		June	52,778	181,459	428,579	51,009	54,357	68,246
		July	56,063	175,341	423,373	51,154	52,538	70,159
		August	59,006	157,626	413,104	53,250	54,630	72,086
		September	63,429	171,203	466,593	58,520	61,605	76,987
		October	66,399	164,098	446,105	57,276	57,610	75,423
		November	71,973	157,350	446,341	58,633	57,073	75,871
		December	63,582	148,379	424,231	50,208	46,196	68,014
**Peru**	**2020**	January	67,238	134,333	429,300	63,893	59,948	79,141
		February	71,421	115,113	424,454	62,276	66,124	77,908
		March	42,013	117,513	269,540	40,893	35,497	60,113
		April	35,792	37,210	53,983	24,430	4,876	48,689
		May	56,416	42,001	70,121	33,310	6,473	61,867
		June	69,905	53,358	94,683	42,126	10,200	73,370

S3 Data. Monthly visits for common conditions (continued)

Country	Year	Month	Anxiety/Depression	Cough, Cold, or Acute Respiratory Infection	Preventative Care	Diabetes	Hypercholesterolemia	Hypertension
**Peru**	**2020**	July	76,749	73,623	105,612	48,591	13,814	81,838
		August	83,248	85,607	109,977	53,435	17,284	86,670
		September	91,855	72,984	140,989	61,623	25,679	96,763
		October	100,487	72,117	189,121	70,182	35,251	103,228
		November	105,783	74,335	202,527	69,608	40,019	103,098
		December	103,750	68,844	214,699	65,460	36,069	95,493
**Peru**	**2021**	January	101,823	88,216	250,373	68,958	35,152	100,143
		February	100,384	83,111	257,759	62,137	27,861	95,186
		March	117,220	109,229	346,968	72,783	36,231	108,230
		April	117,343	126,588	344,926	68,525	34,957	102,912
		May	112,837	109,325	354,645	68,653	35,504	108,344
		June	124,992	105,219	413,441	73,711	40,201	110,463
		July	132,424	120,046	437,439	79,980	47,185	119,357
		August	141,834	129,704	513,927	84,209	57,642	122,599
		September	147,015	120,446	584,354	88,326	61,433	124,228
		October	142,617	127,079	641,661	87,227	60,504	117,462
		November	146,917	143,353	666,080	91,801	62,835	120,667
		December	132,530	152,136	611,516	80,183	51,153	111,343
**Singapore**	**2018**	January	291	25,586	2,858	14,893	3,660	11,550
		February	224	20,412	2,385	12,193	3,115	9,552
		March	249	18,131	2,748	14,612	3,937	11,901
		April	307	19,332	2,738	14,026	3,863	11,562
		May	260	20,255	2,761	14,363	3,722	11,403
		June	261	16,414	2,549	13,282	3,365	10,733
		July	315	21,316	2,808	14,979	4,118	11,595
		August	287	17,325	2,823	14,410	3,786	11,436
		September	280	16,770	2,598	13,165	3,759	10,858
		October	352	19,381	2,909	15,241	4,082	12,041
		November	308	20,141	2,792	13,521	3,722	11,106
		December	244	17,240	2,590	13,467	3,710	10,740
**Singapore**	**2019**	January	363	21,360	2,842	16,011	3,827	12,068
		February	295	16,420	2,372	12,233	3,092	9,137
		March	375	17,144	2,782	14,860	3,990	12,041

S3 Data. Monthly visits for common conditions (continued)

Country	Year	Month	Anxiety/Depression	Cough, Cold, or Acute Respiratory Infection	Preventative Care	Diabetes	Hypercholesterolemia	Hypertension
**Singapore**	**2019**	April	309	18,218	2,806	15,669	4,129	12,996
		May	309	19,542	2,810	14,635	4,428	11,524
		June	268	16,080	2,667	13,050	4,148	10,239
		July	356	21,579	3,135	15,141	4,594	10,971
		August	304	16,353	2,866	13,507	4,392	9,940
		September	304	15,727	2,852	13,167	4,315	9,889
		October	335	19,447	3,168	14,341	4,506	10,330
		November	365	19,400	3,150	13,554	4,451	9,922
		December	272	18,508	3,091	13,330	4,514	10,113
**Singapore**	**2020**	January	329	22,810	2,907	14,057	4,248	9,778
		February	259	19,605	2,372	13,052	3,959	9,397
		March	310	17,197	2,767	13,564	3,899	9,606
		April	219	7,246	2,226	12,711	3,204	7,955
		May	244	2,867	2,409	11,406	3,636	8,488
		June	380	4,987	3,075	15,272	4,717	10,862
		July	394	4,871	3,089	14,337	4,961	10,659
		August	370	4,617	2,902	13,401	4,275	10,038
		September	419	6,902	3,086	13,956	4,278	9,925
		October	437	5,941	2,638	15,004	4,473	10,851
		November	350	4,652	1,876	11,414	4,519	9,374
		December	344	4,831	1,226	9,801	5,067	8,866
**Singapore**	**2021**	January	432	7,109	742	13,506	6,146	11,269
		February	349	6,742	543	12,314	5,589	10,228
		March	487	8,720	611	14,115	6,645	12,260
		April	459	8,434	711	13,333	5,970	11,047
		May	397	8,992	523	12,001	5,092	9,585
		June	452	5,084	619	14,311	5,733	10,823
		July	519	6,802	481	13,558	5,999	11,855
		August	487	7,064	518	12,729	6,053	11,626
		September	494	8,277	578	12,810	5,985	12,179
		October	448	8,411	501	12,390	5,713	10,974
		November	450	7,710	475	12,391	5,941	11,208
		December	496	6,209	451	13,266	6,284	11,774

S3 Data. Monthly visits for common conditions (continued)

Country	Year	Month	Anxiety/Depression	Cough, Cold, or Acute Respiratory Infection	Preventative Care	Diabetes	Hypercholesterolemia	Hypertension
**Sweden**	**2018**	January	2,052	1,810	755	1,218	68	2,303
		February	1,819	1,828	684	1,402	73	2,124
		March	2,010	1,771	727	1,710	76	2,471
		April	1,951	1,405	671	1,680	82	2,232
		May	2,059	1,215	726	1,989	96	2,463
		June	1,833	798	638	1,497	55	1,801
		July	1,290	689	445	551	19	898
		August	1,928	682	698	1,248	53	1,562
		September	1,906	1,111	728	1,700	54	1,970
		October	2,415	1,360	802	1,932	78	2,564
		November	2,310	1,552	788	1,758	72	2,534
		December	1,840	1,363	503	1,368	66	1,867
**Sweden**	**2019**	January	2,591	1,904	736	1,620	88	2,520
		February	2,048	1,765	664	1,650	64	2,323
		March	2,328	1,615	758	2,178	78	2,585
		April	2,217	1,301	653	2,048	93	2,388
		May	2,316	1,195	652	2,316	84	2,550
		June	1,826	990	607	1,777	59	1,791
		July	1,581	832	469	699	28	1,082
		August	2,045	887	620	1,599	41	1,610
		September	2,345	1,234	631	2,379	63	2,286
		October	2,835	1,631	773	2,543	80	2,954
		November	2,659	1,605	769	2,144	94	2,745
		December	2,182	1,566	633	1,779	84	2,359
**Sweden**	**2020**	January	2,715	1,645	679	2,007	90	2,802
		February	2,348	1,896	635	2,123	73	2,609
		March	2,452	1,710	554	1,956	86	2,452
		April	2,199	1,561	432	1,593	54	1,661
		May	2,292	1,088	486	1,657	52	1,736
		June	2,183	758	564	1,624	53	1,693
		July	1,481	373	411	559	30	1,013
		August	2,033	335	592	1,450	43	1,564
		September	2,318	609	669	2,561	55	2,423

S3 Data. Monthly visits for common conditions (continued)

Country	Year	Month	Anxiety/Depression	Cough, Cold, or Acute Respiratory Infection	Preventative Care	Diabetes	Hypercholesterolemia	Hypertension
**Sweden**	**2020**	October	2,683	648	571	2,543	75	2,620
		November	2,651	819	507	2,093	77	2,416
		December	2,354	699	447	1,627	60	1,943
**Sweden**	**2021**	January	2,738	573	488	1,739	64	1,973
		February	2,699	534	563	1,993	66	2,312
		March	3,306	595	732	2,673	83	2,842
		April	2,974	490	596	2,386	83	2,465
		May	3,053	510	576	2,354	83	2,408
		June	2,927	552	536	2,036	69	2,202
		July	1,761	368	368	588	24	1,024
		August	2,441	380	525	1,353	52	1,553
		September	3,101	623	604	2,624	86	2,360
		October	3,116	1,036	614	2,534	79	2,614
		November	3,317	1,443	579	2,300	99	2,871
		December	2,898	1,496	517	1,800	72	2,371
**United States**	**2018**	January	2,138	1,816	3,050	2,379	3,232	3,216
		February	1,887	1,334	2,898	2,056	3,045	3,046
		March	1,888	1,098	3,113	2,162	3,138	3,153
		April	2,006	979	3,186	2,266	2,977	3,119
		May	2,052	820	3,149	2,268	3,210	3,298
		June	1,854	506	3,117	1,999	2,891	3,047
		July	1,903	366	3,150	2,093	2,751	3,024
		August	1,913	428	3,265	2,078	2,945	3,039
		September	1,603	540	2,737	1,888	2,586	2,621
		October	1,943	686	3,529	2,326	3,105	3,300
		November	1,734	761	3,009	1,929	2,810	2,992
		December	1,459	871	2,715	1,643	2,274	2,550
**United States**	**2019**	January	1,951	1,107	3,252	2,000	2,729	2,908
		February	1,635	1,046	2,935	1,775	2,346	2,545
		March	1,806	909	2,846	1,679	2,515	2,606
		April	1,921	640	3,322	1,840	2,707	2,770
		May	1,844	574	3,457	1,744	2,504	2,559

S3 Data. Monthly visits for common conditions (continued)

Country	Year	Month	Anxiety/Depression	Cough, Cold, or Acute Respiratory Infection	Preventative Care	Diabetes	Hypercholesterolemia	Hypertension
**United States**	**2019**	June	1,598	425	2,965	1,544	2,363	2,283
		July	1,702	325	3,358	1,697	2,468	2,505
		August	1,780	330	3,462	1,833	2,592	2,570
		September	1,683	371	3,319	1,727	2,464	2,517
		October	2,113	488	4,100	2,089	2,931	2,974
		November	1,661	459	3,289	1,802	2,536	2,587
		December	1,576	621	3,185	1,655	2,375	2,455
**United States**	**2020**	January	2,136	811	3,837	2,277	2,878	2,748
		February	2,013	627	3,457	1,923	2,579	2,599
		March	1,542	644	2,201	1,584	1,844	1,951
		April	1,467	395	1,028	1,352	1,428	1,684
		May	1,461	192	1,916	1,279	1,705	1,812
		June	1,880	170	3,339	1,852	2,549	2,517
		July	1,836	195	3,391	1,781	2,609	2,483
		August	1,687	159	3,522	1,576	2,532	2,415
		September	1,758	168	3,736	1,661	2,560	2,444
		October	1,748	222	3,812	1,527	2,590	2,426
		November	1,576	295	3,339	1,455	2,266	2,211
		December	1,706	298	3,213	1,447	2,306	2,382
**United States**	**2021**	January	208	65	240	78	153	121
		February	182	42	228	68	167	102
		March	270	58	351	115	250	156
		April	2,110	183	3,509	1,842	2,672	2,725
		May	1,863	189	3,427	1,803	2,438	2,536
		June	1,976	202	4,025	1,903	2,740	2,651
		July	1,806	185	3,672	1,672	2,582	2,537
		August	1,872	253	4,101	1,833	2,688	2,628
		September	1,880	268	4,139	1,723	2,719	2,704
		October	1,955	301	4,309	1,785	2,948	2,830
		November	1,945	248	4,129	1,811	2,923	2,821
		December	1,913	345	3,863	1,702	2,860	2,845
